# Remodeling male coercion and the evolution of sexual autonomy by mate choice

**DOI:** 10.1093/evolut/qpad074

**Published:** 2023-06-01

**Authors:** Samuel S Snow, Richard O Prum

**Affiliations:** Institute for Advanced Study in Toulouse, University of Toulouse 1 Capitole, Toulouse, Occitanie, France; Department of Ecology and Evolutionary Biology, Yale University, New Haven, CT, USA; Department of Ecology and Evolutionary Biology, Yale University, New Haven, CT, USA

**Keywords:** sexual conflict, sexual coercion, sexual selection, mate choice, multimodal mate preference, population-genetic model

## Abstract

Models of sexual conflict over mating, including conflict over indirect benefits of mate choice, have generally presumed that female resistance to male coercion must involve direct confrontation, which can lead to sexually antagonistic coevolutionary arms-races. We built a quantitative model examining the largely ignored possibility that females may evolve new, additional mate preferences for new male traits that undermine male capacity to coerce. Thus, females may “remodel” the coercive capacity of the male phenotype in order to enhance their own sexual autonomy—a novel alternative mechanism by which females may avoid arms-races. We demonstrate that evolutionary “remodeling” is possible, in spite of costs to males, because females that prefer males with protective, autonomy-enhancing traits (traits correlated with lower coercion effectiveness) are likelier to gain indirect benefits of having attractive mates. Our analysis reveals new possibilities for the evolution of systems of sexual conflict over indirect benefits, showing that autonomy-enhancing male traits can act as a “public good,” benefiting all females regardless of mating preferences, leading to oscillatory dynamics; and that preferences for more protective male traits will often be favored relative to preferences for less protective traits, potentially leading to an evolutionary “snowball” of expanding sexual autonomy.

## Introduction

Sexual conflict over mating, wherein the evolutionary interests of males and females are misaligned in mating or fertilization, is typically characterized by the evolution of female resistance to male sexual coercion, often leading to striking sexually antagonistic coevolutionary arms-races (Arnqvist & Rowe, 2002, 2005; Gavrilets & Hayashi, 2006; Gavrilets et al., 2001; Lessells, 2006; Morrow & Arnqvist, 2003; Parker, 2006; Rowe & Arnqvist, 2002; Rowe et al., 2003). Recent work has further established that female resistance can evolve in response to sexual conflict over indirect benefits (fitness benefits of mate choice accruing to females via their offspring in terms of their attractiveness and/or viability; Brennan & Prum, 2012; Snow et al., 2019). Thus, female resistance can reinforce existing female mating preferences, rather than leading simply to the avoidance of physical harm (Brennan & Prum, 2012; Snow et al., 2019). Waterfowl provide a well-known example of resistance to retain the indirect genetic benefits of mating with their preferred social partners: in response to the evolution of elaborate male penis morphologies that facilitate the forced fertilization of females, females of some species of waterfowl have coevolved convoluted genital tracts that physically hinder unwanted fertilization (Brennan & Prum, 2012; Brennan et al., 2007, 2010; Snow et al., 2019).

Most previous models characterizing evolutionary outcomes of sexual conflict over mating have presumed that evolved female responses to male coercion require direct female interaction with the coercive male morphology/behavior; females can either resist more intensely or become less sensitive to the coercive attacks (e.g., Arnqvist & Rowe, 2005; Gavrilets & Hayashi, 2006; Rowe et al., 2005, but see Rosenthal & Servedio, 1999). Either response can produce selection for further exaggeration of coercive traits in males (Arnqvist & Rowe, 2005; Gavrilets & Hayashi, 2006; Gavrilets et al., 2001; Härdling & Smith, 2005; Lessells, 2006; Parker, 2006; Snow et al., 2019). As an alternative to direct confrontation and the classic arms-race scenario, in this article we explore the largely ignored possibility that females may evolve new, additional mating preferences that undermine male capacity to coerce and lead to reduced coercion, and increased freedom of mate choice.

In pursuit of mathematical simplicity, most previous models of sexual conflict over mating do not distinguish between male coercion and display, or female choice or resistance (e.g., Arnqvist & Rowe, 2005; Brooks & Griffith, 2010; Cordero & Eberhard, 2003; Eberhard, 2005; Holland & Rice, 1998; Kokko, 2005; reviewed in Brennan & Prum, 2012). However, examination of sexual conflict over indirect benefits reveals that these potentially concurrent processes can be meaningfully distinguished when sexual coercion is defined as any (male) action biasing mating in a way that subverts an existing (female) mating preference for a display (Brennan & Prum, 2012; Snow et al., 2019). Previous work has shown that coercion resistance traits in females will be selectively favored as long as females can attain some indirect benefits of mating with their preferred males based on display, and coercion causes them to mate with preferred males less often than otherwise (Snow et al., 2019). These insights open the door to inquiry into more complex systems that include features of simultaneous preference for male display and male coercion, as well as reimagining the diverse ways that females resist sexual coercion.

Prum (2015, 2017) has suggested a novel alternative mechanism by which females may mitigate sexual conflict over indirect benefits without direct confrontation by evolving new mate preferences that actively “remodel” male coercive capacities. Rather than evolving resistance traits that directly interfere with male coercion and potentially inciting an arms race, females may evolve an additional mate preference for a new male trait that has the benefit of enhancing female freedom of mate choice (referred to as “sexual autonomy”; Brennan & Prum, 2012; Prum, 2017; Snow et al., 2019). We will call a novel female mating preference for a male display trait with influence on coercion efficacy a “remodeling preference,” and the corresponding display trait an “autonomy-enhancing trait.”

Imagine a population in which fertilization determination mechanisms are in equilibrium between female mate choice based on male display and male sexual coercion. We hypothesize that a new female mating preference for a novel male trait incidentally correlated with lower effectiveness of sexual coercion can evolve because it disrupts this equilibrium in favor of female mate choice, reducing the efficacy of sexual coercion, and expanding female sexual autonomy.

This proposal is distinct from females altering their preferences for a display (e.g., Kokko, 2005). Male coercion specifically functions to subvert females’ ability to realize an existing mating preference, and therefore females must evolve independent resistance (Snow et al., 2019), or in this case, additional preferences unaffected by the existing male coercion mechanism, in order to reduce the effectiveness of coercion and expand sexual autonomy. Similarly, remodeling preferences are distinct from classic sexual selection, wherein females can be said to actively shape male phenotypes, because the classic preference is not tied to male coercive capacity and therefore the efficacy of another preference.

**Figure 1.**
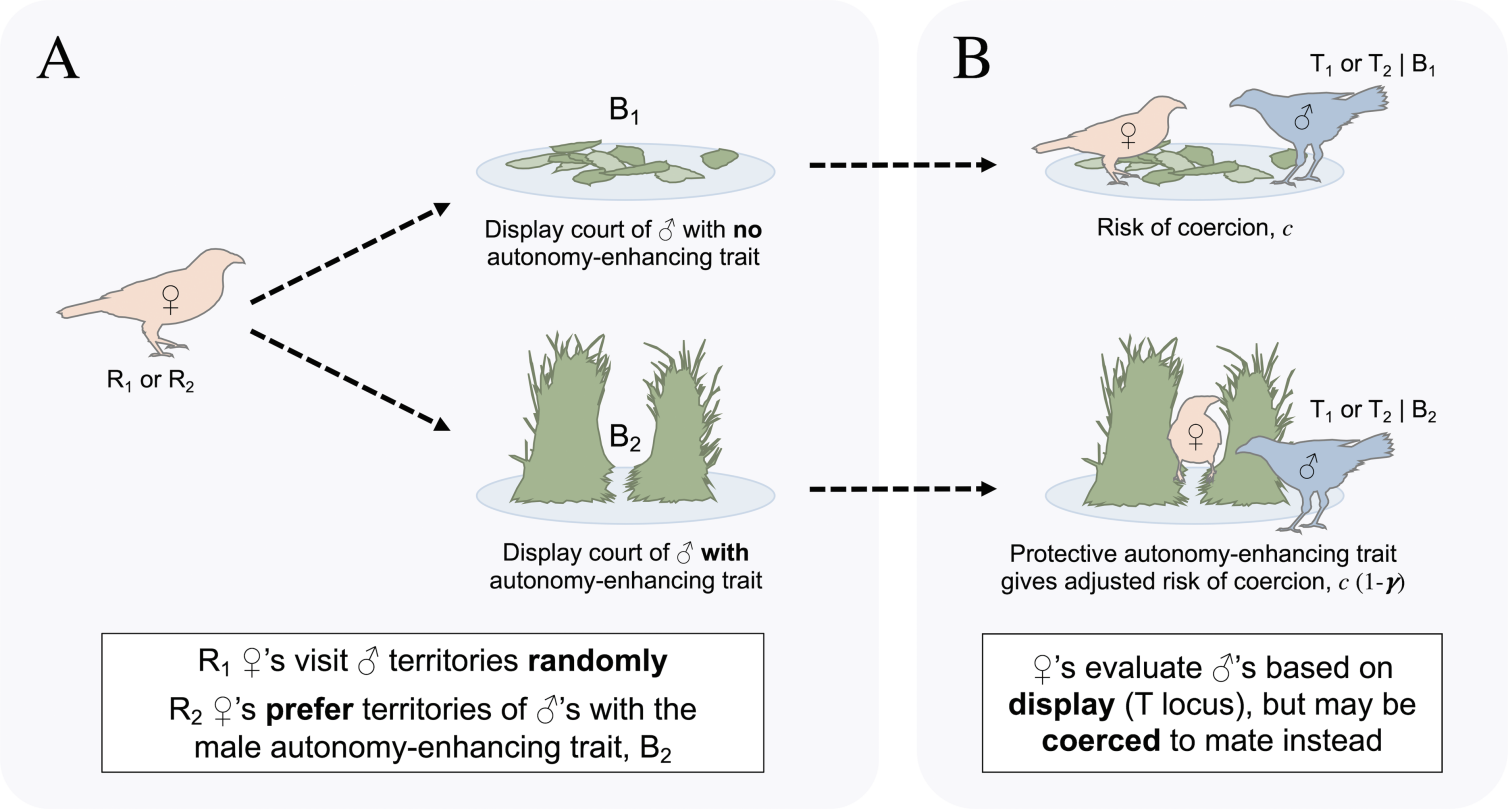
Schematic of the process of the distribution of matings captured by the model, using the example of bowerbirds (Ptilonorhynchidae). (A) First, females choose to visit male territories according to their remodeling preference (coded at the R locus) for the corresponding autonomy-enhancing trait in males (coded at the B locus; represented by the protective architecture of the courtship display “avenue bower” built by the male). (B) Once on a territory, a female can accept or reject a male based his display attractiveness (coded at the T locus), or she may be coerced into mating without the opportunity to reject (with probability *c*). Females that associate with B_2_ males are physically protected from the male’s own coercive attempts by the bower structure [giving an adjusted risk of coercion *c*(1−*γ*)].

Male “autonomy-enhancing traits” that are subject to these novel preferences may be particular morphologies such as reduced weapons, behavioral cues such as displays that can be seen from a safe distance or display postures or orientations that reduce the likelihood of coercion, or aspects of extended phenotype that interfere with a males’ own ability to sexually coerce. For example, male bowerbirds (Ptilonorhynchidae) build courtship display bowers with a distinctive architectural design—such as an “avenue bower” that allows a female to perch between two walls of sticks—that protects females from the males’ own potential forced copulation attempts (Figure 1A; Borgia, 1995; Borgia & Presgraves, 1998; Patricelli et al., 2002; Prum, 2015, 2017). Females that prefer to visit males with autonomy-enhancing traits will have an advantage because they are less likely to be coerced, and thus more likely than other females to gain the indirect benefits of mating with attractive males. By this mechanism, females may be able to evolutionarily remodel male coercive capacity in spite of coercion success and potential costs to males, and mitigate sexual conflict over indirect benefits without evolving direct resistance that would possibly incite a sexually antagonistic co-evolutionary arms race (Brooks & Griffith, 2010; Kokko, 2005).

The plausibility of the remodeling mechanism is difficult to intuit because the hypothesis requires complex interactions among the effects of several traits—an attractive male display, female preference for that display, male coercion, a female preference for an additional “remodeled” autonomy-enhancing trait, and the autonomy-enhancing trait in males—which implies multiple higher-order effects propagated through genetic correlations arising from indirect selection. Specifically, we want to know if a male autonomy-enhancing trait can evolve by female choice because it enhances female sexual autonomy—that is, it increases the proportion of matings free from male coercion. We ask whether indirect selection on female preference promoting the evolution of autonomy-enhancing traits via genetic correlations with male attractiveness could be enough to overcome the costs incurred by males in terms of potential lower viability and lost coercive mating opportunities. We built a quantitative proof-of-concept theoretical model to examine this new possibility for the evolutionary trajectory of systems in sexual conflict over indirect benefits.

## The model

We built a three-locus population genetic model. For simplicity, we assumed a polygynous, haploid system with two alleles per locus based on classic sexual selection models (Barton & Turelli, 1991; Kirkpatrick, 1982), expanded to incorporate three loci. All loci are autosomal, but with sex-limited expression. Males express two trait loci: T for a display trait, and B for an autonomy-enhancing trait. Females express “remodeling” preference for the male autonomy-enhancing trait at locus R. Males with the T_2_ allele produce an attractive display ornament while T_1_ males do not; B_2_ males produce the autonomy-enhancing trait while B_1_ males do not. Females with the R_2_ allele have a biased behavioral preference for visiting males with the autonomy-enhancing trait (B_2_ males), whereas R_1_ females visit males randomly with respect to males’ alleles at the B locus (See Table 1 for a summary of terms).

**Table 1.** Summary of terms used in the three-locus population genetic model.

T_2_/T_1_	Allele for male attractive display trait/allele for unattractive display trait; lowercase denotes the respective allele frequencies
B_2_/B_1_	Allele for the male autonomy-enhancing trait/allele for the lack of the autonomy-enhancing trait
R_2_/R_1_	Allele for female preference for visiting males with the autonomy-enhancing trait (B_2_ males)/Allele for visiting males randomly with respect to males’ alleles at the B locus
*s*_*t*_	Viability cost to T_2_ males of producing an attractive display trait
*s*_*b*_	Viability cost to B_2_ males of producing the autonomy-enhancing trait
*s*_*r*_	Viability cost to R_2_ females of preferring males with the autonomy-enhancing trait (B_2_ males)
*u*	Mutation bias parameter: percent of the frequency of the T_2_ allele that mutates to the T_1_ allele each generation after mating and recombination
*a*_*t*_+1	Factor by which all females prefer to mate with T_2_ males
*a*_*b*_+1	Factor by which R_2_ females prefer to visit males with the autonomy-enhancing trait (B_2_ males)
*c*	Likelihood that a female gets coerced into mating without the opportunity to accept or reject a male based on his display upon visiting a male that lacks the autonomy-enhancing trait
*γ*	Effectiveness of the protection from coercion conferred by the autonomy-enhancing trait in B_2_ males

For tractability, female preference for display and male coercion are treated as follows: (a) all females in the population have a preference for mating with attractive T_2_ males by a factor of (*a*_*t*_ + 1) relative to T_1_ males and (b) when visited by a female, all males have the capacity to attempt coercing females into mating with them without affording females the opportunity to choose based on their display. Our aim is to examine the plausibility of the evolution of female preferences for autonomy-enhancing traits in males via selection to mitigate sexual conflict over indirect benefits; having female preference for display and male coercion fixed allows us to set up a scenario of sexual conflict over indirect benefits (Snow et al., 2019; see *Analyses*) and investigate the dynamics of interest while using the simplest model framework (i.e., tracking the fewest loci) possible.

Further, we assume biased mutation at the male display (T) locus, such that a percentage of the frequency of the attractive T_2_ allele, *u*, mutates to T_1_ every generation after mating and recombination (Pomiankowski et al., 1991). This assumption is commonly used in sexual selection models as a straightforward mechanism to maintain diversity at key loci (which would otherwise be unrealistically depleted by selection in our simple model; Houle, 1992; Rowe & Houle, 1996), allowing for the maintenance of the indirect benefits of mate choice associated with, for example, Fisherian ornaments (Pomiankowski & Iwasa, 1993; Pomiankowski et al., 1991) or viability mediated by an ornament, in the case of good-genes models (Iwasa & Pomiankowski, 1994, 1999; Iwasa et al., 1991). Others have assumed a constant frequency to approximate a quantitative trait of constant variance in order to achieve a similar effect of allowing sexual selection to be ongoing in a simple model framework (Bank et al., 2012; Dhole et al., 2018; Snow et al., 2019; Tazzyman et al., 2014). Although the assumption of biased (as opposed to bidirectional) mutation is not strictly necessary for the maintenance of variation in the simple biallelic framework employed here (Veller et al., 2020), we use mutation bias to promote conceptual continuity with other quantitative sexual selection frameworks. For the simulation results we present in the main text, we employ a large mutation rate (0.1) that corresponds more closely to an assumption of constant variance while still allowing for exact recursions. We also explored a version with a more traditional small mutation rate, one order of magnitude smaller than selection on the display trait, which yielded qualitatively similar results (compare Supplementary Figures S1 and S2). The key consideration is that any selection on female preference is on the same order of magnitude as the mutation rate (or one order smaller, depending on the structure of the model) in order for the possibility of mutation–selection balance to maintain costly preference (Iwasa & Pomiankowski, 1994; Iwasa et al., 1991; Pomiankowski et al., 1991). In the present case, because indirect benefits of mating with attractive males are mediated by the autonomy-enhancing trait, (similar to how viability “good genes” are mediated by a display ornament) *s*_*r*_ must be roughly one order smaller than mutation.

### Ecological and behavioral assumptions

We assume that the attractive display (T_2_) and autonomy-enhancing trait (B_2_) each come with a viability cost such that a T_2_ male’s survival until mating season is reduced by a factor of (1−*s*_*t*_), and a B_2_ male’s survival is reduced by a factor of (1−*s*_*b*_), relative to T_1_ or B_1_ males, respectively. We also assume a small cost of the female remodeling preference such that R_2_ females have a (1−*s*_*r*_) chance of surviving to mate relative to R_1_ females.

After viability selection, mating occurs. All surviving females are ultimately fertilized by one male each, but males may mate multiply and vary in relative success. Male mating success is determined by a combination of the attractiveness of their autonomy-enhancing trait, the attractiveness of their display, *and* their ability to coerce females. Females seek out mates by visiting males at their territories (Figure 1A; this model could also be interpreted as females choosing to associate with particular males). Females without a preference for the male autonomy-enhancing trait (R_1_ females) visit territories randomly. Females with a remodeling preference (R_2_ females) bias their territory visits toward males with the autonomy-enhancing trait (B_2_ males) by a factor of (*a*_*b*_ + 1) relative to males that lack it.

Once on a male’s territory, the female can choose to accept or reject the male based on his display (his T allele). Females will choose to accept a T_1_ male with a probability of 1/(*a*_*t*_ + 2), and a T_2_ male with a probability of (*a*_*t*_ + 1)/(*a*_*t*_ + 2). Thus, we assume the baseline probability of accepting or rejecting a given male for a female without preference for display (*a*_*t*_ = 0) is ½. Alternatively, the visiting female may be coerced into mating without being afforded the opportunity to possibly reject him (Figure 1B). Females that visit the territories of males lacking the autonomy-enhancing trait (B_1_ males) have a probability *c* of getting coerced to mate. The autonomy-enhancing trait, B_2_, lowers a male’s ability to coerce, and thus offers protection and increased autonomy to females. Females that visit B_2_ males have an adjusted probability of being coercively mated of c*(1 − *γ*), where *γ* is the effectiveness of the protection conferred by the male trait. (See Table 2 for a matrix of the relative frequencies of mating between the various genotypes.) After matings are distributed, there is standard free recombination of alleles among gametes and finally biased mutation at the T locus (from T_2_ to T_1_ alleles at rate *u*) yielding genotype frequencies for the next generation (see Supplementary Appendix S1 for development of model equations and Supplementary File S1 for full model code).

**Table 2.** Frequencies of matings between each of the eight genotypes. Although the model follows all genotypes independently, to aid in interpretation, each row gathers together all male genotype frequencies having the same alleles at the T and B loci; since these traits are sex-limited, these groups of genotypes function identically in mating. Each term $x_{ij}^{\prime }.$ is the sum of the frequencies of males with genotype T_*i*_B_*j*_ (T_*i*_B_*j*_R_1_ and T_*i*_B_*j*_R_2_). Similarly, each column gathers all female genotype frequencies having the allele R_1_ or R_2_, yielding $y_{\cdot \cdot 1}^{\prime }$ and $y_{\cdot \cdot 2}^{\prime }$. The prime notation is a reminder that the genotype frequencies have already been adjusted by viability selection. The “*z*” terms are normalizing factors, where $z_{b}=x_{11\cdot }^{\prime }+x_{21\cdot }^{\prime }+x_{12\cdot }^{\prime }\left(a_{b}+1\right)+x_{22\cdot }^{\prime }\left(a_{b}+1\right)$, and *z*_1_ and *z*_2_ are the sums of the numerators in their columns divided by $y_{\cdot \cdot 1}^{\prime }$ and $y_{\cdot \cdot 2}^{\prime }$ respectively.

Males	Females	
	… R_1_	… R_2_
T_1_B_1_ …	$\frac{y_{\cdot \cdot 1}^{\prime }\left(x_{11\cdot }^{\prime }\left(c+\left(1-c\right)\left(\frac{1}{a_{t}+2}\right)\right)\right)}{z_{1}}$	$\frac{y_{\cdot \cdot 2}^{\prime }\left(\frac{x_{11\cdot }^{\prime }}{z_{b}}\left(c+\left(1-c\right)\left(\frac{1}{a_{t}+2}\right)\right)\right)}{z_{2}}$
T_2_B_1_ …	$\frac{y_{\cdot \cdot 1}^{\prime }\left(x_{21\cdot }^{\prime }\left(c+\left(1-c\right)\left(\frac{a_{t}+1}{a_{t}+2}\right)\right)\right)}{z_{1}}$	$\frac{y_{\cdot \cdot 2}^{\prime }\left(\frac{x_{21\cdot }^{\prime }}{z_{b}}\left(c+\left(1-c\right)\left(\frac{a_{t}+1}{a_{t}+2}\right)\right)\right)}{z_{2}}$
T_1_B_2_ …	$\frac{y_{\cdot \cdot 1}^{\prime }\left(x_{12\cdot }^{\prime }\left(c\left(1-\gamma \right)+\left(1-c\left(1-\gamma \right)\right)\left(\frac{1}{a_{t}+2}\right)\right)\right)}{z_{1}}$	$\frac{y_{\cdot \cdot 2}^{\prime }\left(\frac{x_{12\cdot }^{\prime }\left(a_{b}+1\right)}{z_{b}}\left(c\left(1-\gamma \right)+\left(1-c\left(1-\gamma \right)\right)\left(\frac{1}{a_{t}+2}\right)\right)\right)}{z_{2}}$
T_2_B_2_ …	$\frac{y_{\cdot \cdot 1}^{\prime }\left(x_{22\cdot }^{\prime }\left(c\left(1-\gamma \right)+\left(1-c\left(1-\gamma \right)\right)\left(\frac{a_{t}+1}{a_{t}+2}\right)\right)\right)}{z_{1}}$	$\frac{y_{\cdot \cdot 2}^{\prime }\left(\frac{x_{22\cdot }^{\prime }\left(a_{b}+1\right)}{z_{b}}\left(c\left(1-\gamma \right)+\left(1-c\left(1-\gamma \right)\right)\left(\frac{a_{t}+1}{a_{t}+2}\right)\right)\right)}{z_{2}}$

We hypothesize that R_2_ females, which prefer the autonomy-enhancing male trait, B_2_, will be selectively favored because B_2_ will become genetically correlated with the indirect benefits of mating with attractive T_2_ males. Therefore, if B_2_ persists in the population, we expect to see correlations arise between R_2_ and B_2_, between T_2_ and B_2_, and between R_2_ and T_2_. Further, we will explore the effect of *a*_*t*_, *a*_*b*_, *s*_*r*_, *c*, and *γ* on whether the B_2_ allele is able to persist.

### Analyses

We analyzed behavior of the model and equilibria using numerical simulation of the recursion equations, as the complexity of the model precludes standard eigenvalue analysis (Supplementary File S1). For our numerical simulations, we began with a population at an internal equilibrium for the frequency of the T_2_ allele (calculated with the R_2_ and B_2_ alleles fixed at zero; See Supplementary Appendix S2 and Supplementary File S2):


$$\begin{array}{l} {\hat{t}}_{2}=\frac{1-u+\frac{2(1+c+a_{t}c)u}{s_{t}+cs_{t}+a_{t}(c+s_{t}-1)}}{1+u}. \end{array}$$

This means that there is a stable balance between the combined effect of sexual selection on the male display trait and the effect of male sexual coercion (the latter of which weakens the effect of sexual selection), mutation, and viability selection on the display trait. Although both display and coercion bias females’ mating, it is important to note that these processes are distinct and function in a noninterchangeable way: the term sexual coercion is meaningful because this behavior functions in a system where females have an existing mating preference for a display. Since coercion specifically circumvents females’ preference for display, it does not simply add to the effect of display (as an additional ornament might; for example, Pomiankowski & Iwasa, 1993), and since it dampens—but does not negate—the benefits females attain though mate choice, it has the property of producing sexual conflict over indirect benefits (Snow et al., 2019). The “broad-sense” conception of sexual coercion (Brennan & Prum, 2012) invoked here includes obvious physical restraint and harassment, but also other diverse mechanisms such as male–male competition that might interfere with female mate choice (Wong & Candolin, 2005). Note that employing coercion and display thusly means that this paper does not explore parameter conditions for which coercion (*c*) is so strong relative to female preference (*a*_*t*_) that sexual selection is insufficient to maintain the male display trait in mutation-selection balance given the costs (*s*_*t*_). We can get a sense of the overall effect that coercion has on the system by deriving an expression for the adjusted realized magnitude of the parameter for female preference strength, *a*_*t*_, under the conditions for the nonzero initial equilibrium frequency for the T_2_ allele (see Supplementary Appendix S2 and Supplementary File S2). Essentially, we can ask what adjusted value of *a*_*t*_ would produce the equivalent equilibrium value of t_2_ if coercion were absent:


$$\begin{array}{l} {a_{t}}_{adj}=\frac{a_{t}(1-c)}{c(a_{t}+1)+1} \end{array}$$

We can see that adjusted *a*_*t*_ under coercion will always be smaller than the actual *a*_*t*_. This of course does not indicate that coercion causes females to have weaker preferences, but rather shows that coercion causes females’ preferences to be realized less effectively. As *c* approaches 1, ${a_{t}}_{adj}$ approaches zero, demonstrating that when coercion is perfect, females have no opportunity to exercise their preferences. The expression for ${a_{t}}_{adj}$ holds as along as the conditions for the stable initial nonzero T_2_ equilibrium are met (Supplementary Appendix S2).

Previous work has shown that sexual coercion concurrent with females retaining some indirect benefits of mating with attractive males represents a potentially unstable condition sufficient to produce selection for direct female resistance (Snow et al., 2019). Here we used this balance between sexual selection and coercion to examine the plausibility of the evolution of remodeling preferences as an alternative to direct confrontation with the male coercive strategy. Accordingly, into this equilibrium condition, we then introduced the R_2_ and B_2_ alleles at very low frequencies.

To establish whether, and under what conditions, selection for increased indirect benefits of autonomous mate choice could drive the evolution of female remodeling preferences and the corresponding male traits, we used the numerical simulations to explore the effect of the various parameters on whether these traits became established in the population. To gain further insight, we graphically inspected the evolutionary trajectories of the traits and genetic correlations in sample numerical simulations representative of outcomes where either the autonomy-enhancing male trait persisted or did not. Moreover, we undertook a weak selection approximation of the model equations to examine the leading-order mechanisms and forces involved in more detail. Finally, we looked at whether more protective male autonomy-enhancing traits would be favored relative to less protective ones by examining a version of the model that controls for the effects of arbitrary, Fisherian sexual selection on the autonomy-enhancing trait.

We performed all analyses and numerical simulations using Wolfram *Mathematica* (Wolfram, 2016). We ran numerical simulations until all genotype frequencies and genetic correlations were constant to seven decimal places, and then, to avoid incorrect conclusions arising from slow convergence on an unstable point, we applied a small random perturbation and allowed the simulation to continue until again constant to seven decimal places. For a full summary of parameter ranges investigated, see Supplementary Figures S1 and S2.

## Results

### Remodeling preferences and traits can evolve

**Figure 2.**
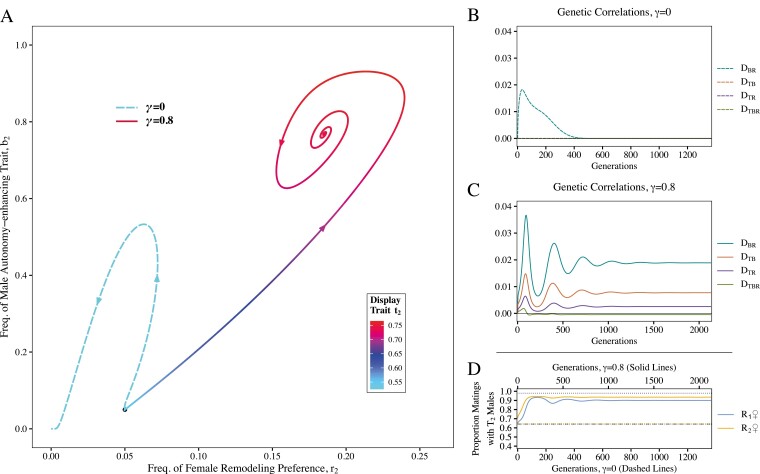
Remodeling preferences and traits evolve to enhance female sexual autonomy, as shown in the case where the male autonomy-enhancing trait confers protection to females against male coercion (solid lines; *γ* = 0.8), in contrast to the case where the autonomy-enhancing trait is not protective (dashed lines; *γ* = 0). (A) Allele frequencies through time for *γ* = 0 and *γ* = 0.8. The black dot is the initial frequency (*b*_2_ = *r*_2_ = 0.05); arrows show the trajectory of the allele frequencies through time. The lines’ colors correspond to the frequency of the male attractive display trait (*t*_2_; initial freq. = 0.525). (B) Two- and three-way genetic correlations (linkage disequilibria) among the three loci though time for the nonprotective case. (C) Genetic correlations among the three loci though time for the protective case. Dashed lines for D_TB_, D_TR_, and D_TBR_ are essentially zero throughout and therefore overlap. (D) Proportion of matings in the population with attractive T_2_ males through time. The lower black dotted line is the initial proportion and the upper black dotted line is the proportion of matings with T_2_ males that females would attain if there were no coercion (i.e., full sexual autonomy). The orange lines are the proportion of T_2_ males attained by R_2_ females preferring the autonomy-enhancing trait; the blue lines are the proportion for R_1_ females that mate randomly with respect to the autonomy-enhancing trait. Dashed lines for the *γ* = 0 case overlap with each other and the bottom black dotted line—the autonomy-enhancing trait is not protective and thus there is no increase in autonomy or advantage to having the R_2_ allele in this scenario. Other parameters are the same in both cases: *a*_*t*_ = 12, *a*_*b*_ = 12, *c* = 0.5, *s*_*t*_ = 0.1, *s*_*b*_ = 0.1, *u* = 0.1, *s*_*r*_ = 0.01. See Supplementary Figure S3 for allele-frequency-by-time representations of the data shown in (A).

The remodeling preference and trait—R_2_ and B_2_ alleles—fail to become established in the baseline case where the autonomy-enhancing trait does not protect females from coercion (*γ* = 0; Dashed lines in Figure 2). Because we assumed a small viability cost to females with remodeling preference, arbitrary Fisherian sexual selection is insufficient to allow B_2_ and R_2_ to persist at equilibrium in the absence of additional sources of selection, regardless of initial allele frequencies (Kirkpatrick & Barton, 1997; Pomiankowski et al., 1991).

In contrast, when protection conferred by the autonomy-enhancing trait is at a requisite level (e.g., *γ* = 0.8; solid lines in Figure 2), the male trait increases in frequency and persists in the population at equilibrium, due to its evolved correlation with females’ sexual autonomy (Figure 2A, solid line). Unlike in the *γ* = 0 case, females are protected from coercion and can mate with males having an attractive display (T_2_) at a frequency closer to what they would attain without coercion (Figure 2D). We can see that as the autonomy-enhancing trait proliferates, the frequency of the T_2_ allele concurrently increases as sexual selection on display is strengthened (shift in color of solid line from blue to red; Figure 2A). How closely the final frequency of the T_2_ allele approaches the expectation under full autonomy depends on the combined influence of the protection of the autonomy-enhancing trait and the final frequency of the B_2_ allele in reducing the overall sexual selection-dampening effect of coercion (Equation 1; Supplementary Appendix S2).

The evolution of the B_2_ allele involves genetic correlations among the loci (Figure 2B and C). Whether the male trait effectively enhances female autonomy or not, it initially evolves due to sexual selection via females carrying the R_2_ allele, producing correlation between the autonomy-enhancing trait and female preference for it (D_BR_; Figure 2B and C). In the case that the male autonomy-enhancing trait confers protection (*γ* = 0.8; solid lines), the autonomy-enhancing trait becomes correlated with the T_2_ allele because females who visit the territories of B_2_ males are more likely to mate with attractive males (D_TB_; Figure 2C). Additionally, this produces positive correlation between the display trait and the female remodeling preference (D_TR_; Figure 2C), which produces additional positive indirect selection on the R_2_ allele, which boosts sexual selection on B_2_, and so on. This feedback loop allows the female preference for the autonomy-enhancing trait to overcome its viability cost, *s*_*r*_, and allows B_2_ to increase in frequency and persist at equilibrium (*b*_2_ > 0), as opposed to being lost (*b*_2_ = 0).

In cases where the male autonomy-enhancing trait persists, as the population approaches equilibrium, we often observe oscillations when there is a viability cost (*s*_*r*_ > 0) to remodeling preference (Figure 2A). This result reveals an interesting aspect of the process of remodeling: male autonomy-enhancing traits will benefit females who don’t explicitly prefer them. As B_2_ proliferates, fewer matings are coerced since females that visit the territories of B_2_ males are protected (Figure 2D). Females with a preference for the autonomy-enhancing trait are at an advantage because they are able to more reliably mate with attractive T_2_ males (Figure 2D, solid orange line). However, once the autonomy-enhancing trait is common enough, R_1_ females without remodeling preference are likely to mate at a B_2_ male’s territory simply by chance (Figure 2D, solid blue line). Thus, R_1_ females can reap the benefits of enhanced autonomy of mate choice without paying the costs of seeking it out, so R_1_ females become favored until B_2_ decreases again (yielding a spiraling pattern; Figure 2A). In all our numerical simulations where they occur (Supplementary Figure S1) these oscillations invariably converge to a stable equilibrium as in Figure 2A.

### Weak selection approximation

We examined the leading terms of a weak selection approximation of the model equations to gain further insight into the mechanisms underlying the evolution of male autonomy-enhancing traits and female preferences for them (Full calculation available in Supplementary File S4). For the approximation, we assumed weak selection (small *s*_*t*_, *s*_*b*_, and *s*_*r*_), weak mutation (small *u*), weak female preferences (small *a*_*t*_ and *a*_*b*_), and weak male coercion (*c*), while allowing the key parameter of effectiveness of the male autonomy-enhancing trait in diminishing the likelihood of coercion (*γ*) to remain at any strength. We approximated expressions for Δ*r*_2_, Δ*b*_2_, and Δ*t*_2_ to the first order with respect to the parameters assumed to be weak and under the assumption of quasi-linkage equilibrium for the genetic correlations, $\hat{D}$, among the T_2_, B_2_, and R_2_ alleles:


$$\begin{array}{l} \Delta r_{2\_approx}\approx \frac{1}{2}({\hat{D}}_{BR}\beta +{\hat{D}}_{TR}\theta -s_{r}r_{1}r_{2}) \end{array}$$

$$\begin{array}{l} \Delta b_{2\_approx}\approx \frac{1}{2}(\beta b_{1}b_{2}+{\hat{D}}_{TB}\theta -{\hat{D}}_{BR}s_{r}) \end{array}$$

$$\begin{array}{l} \Delta t_{2\_approx}\approx \frac{1}{2}(\theta t_{1}t_{2}+{\hat{D}}_{TB}\beta -{\hat{D}}_{TR}s_{r}-2ut_{2}) \end{array}$$

where $\theta =(a_{t}-s_{t})$ and $\beta =(a_{b}r_{2}-(s_{b}+c\gamma ))$.

The terms *θ* and *β* are, to the first order, the coefficients of direct selection at the T and B loci, respectively. The *θ* term encapsulates the opposing forces of sexual selection on the display trait (*a*_*t*_) and viability selection (*s*_*t*_). The *β* term shows that the evolution of the male autonomy-enhancing trait hinges on the existence and strength of female preference for it (*a*_*b*_*r*_*2*_) and that this is countered by viability selection (*s*_*b*_), the magnitude of coercion *c*, and interestingly, the effectiveness of autonomy-enhancing trait protection, *γ*. If *γ* is nonzero, having the autonomy-enhancing trait (B_2_ allele) hinders a male’s ability to coerce mates while B_1_ males remain free to coerce, and the more effective coercion is (the larger *c* is), the more B_2_ males stand to lose. Thus, the male autonomy-enhancing trait will only be favored with strong and prevalent enough female preference for it, so it is important to examine the factors contributing to the evolution of the R_2_ allele.

Looking at the expression for Δr_2_approx_, we can see that R_2_ can have positive evolution only via indirect selection mediated by its correlations with B_2_ and T_2_ (${\hat{D}}_{BR}$ and ${\hat{D}}_{TR}$, respectively). We determined the values of all genetic correlations at QLE to the third order under the assumptions of weak selection, preferences, and coercion as follows:


$$\begin{array}{ll} & {\hat{D}}_{BR}\approx \frac{1}{2}a_{b}b_{1}b_{2}r_{1}r_{2}(1+\frac{1}{2}(a_{b}\left(r_{2}-2b_{2}\left(r_{2}+1\right)\right)\\ & -3\left(\left(1-2b_{2}\right)\left(s_{b}+c\gamma \right)+\left(1-2r_{2}\right)s_{r}\right))\\ & +\frac{1}{4}(a_{b}^{2}\left(b_{2}^{2}\left(7\left(r_{2}+1\right)r_{2}+4\right)-b_{2}r_{2}\left(7r_{2}+3\right)+r_{2}^{2}\right)\\ & -2a_{b}(\;\;(\;\left(3-14b_{2}^{}\left(1-b_{2}\right)\right)r_{2}+\left(8b_{2}-5\right)b_{2})\left(s_{b}+c\gamma \right)\\ & +s_{r}(b_{2}(10r_{2}^{2}-3)+r_{2}\left(3-5r_{2}\right)\;\;\;))\\ & -c\gamma (\left(1-2b_{2}\right)\left(6a_{t}\left(1-2t_{2}\right)-6c-10\left(1-2r_{2}\right)s_{r}\right)\\ & -3\left(1-2b_{2}\left(4-5b_{2}\right)\right)c\gamma )+2s_{b}(6\left(1-5b_{2}\left(1-b_{2}\right)\right)c\gamma \\ & +5\left(1-2b_{2}\right)\left(1-2r_{2}\right)s_{r})\\ & +3\left(1-2b_{2}\left(4-5b_{2}\right)\right)s_{b}^{2}+3\left(1-2r_{2}\left(4-5r_{2}\right)\right)s_{r}^{2})\;\;) \end{array}$$

$$\begin{array}{ll} & {\hat{D}}_{TB}\approx a_{t}c\gamma t_{1}t_{2}b_{1}b_{2}(1-\frac{1}{2}(3\left(\left(1-2b_{2}\right)\left(s_{b}-a_{b}r_{2}\right)+s_{t}\left(1-2t_{2}\right)\right)\\ & -2a_{t}\left(1-3t_{2}\right)+c\left(\left(1-6b_{2}\right)\gamma +4\right)+4u)\;\;) \end{array}$$

$$\begin{array}{l} {\hat{D}}_{TR}\approx 2a_{t}a_{b}c\gamma t_{1}t_{2}r_{1}r_{2}b_{1}b_{2} \end{array}$$

$$\begin{array}{l} {\hat{D}}_{TBR}\approx \frac{2}{3}a_{t}a_{b}c\gamma t_{1}t_{2}r_{1}r_{2}b_{1}b_{2}(1-2b_{2}) \end{array}$$

**Figure 3.**
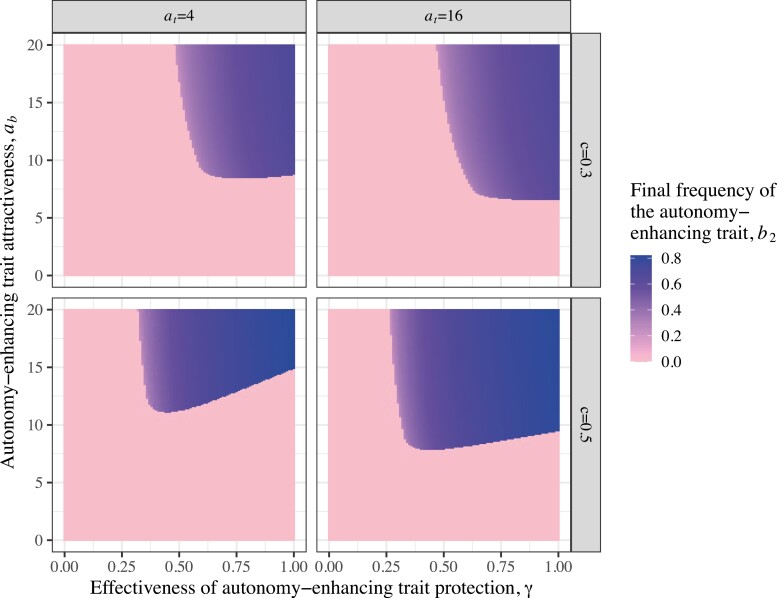
Results of numerical simulations of the model for the persistence of the male autonomy-enhancing trait (B_2_) through parameter space. Color represents the final frequency of the B_2_ allele at equilibrium. Parameter sets shown here are: *a*_*t*_ = {4,16}, *c* = {0.3,0.5}, *s*_*t*_ = 0.1, *s*_*b*_ = 0.1, *u* = 0.1, *s*_*r*_ = 0.01, *a*_*b*_ = 0–20 at increments of 0.1, and *γ* = 0–1 at increments of 0.01.

The correlation between female remodeling preference and the male display trait, ${\hat{D}}_{TR}$, is mediated by (and increasing in) *a*_*t*_, *a*_*b*_, *c*, *γ*, as well as the variation at the B locus, demonstrating how the protectiveness of the autonomy-enhancing trait is key in producing an association between female remodeling preference and the attractive male display trait, which in turn can bolster the evolution of R_2_ in females. In our model, this is the force that allows female remodeling preference to persist at equilibrium if it is costly (*s*_*r*_ > 0; Figures 2, 3, and 5).

**Figure 4.**
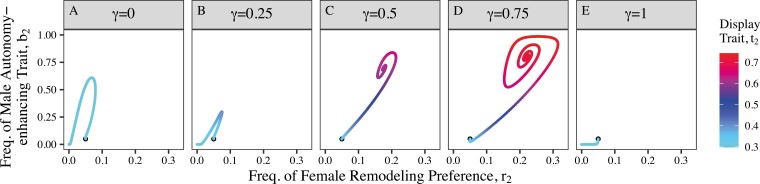
For certain parameter sets, (such as shown here: *a*_*b*_ = 15, *a*_*t*_ = 8, *c* = 0.6) the effectiveness conferred by the male autonomy-enhancing trait, (*γ*; having values at increments of 0.25 across panels A–E) can potentially be either too weak (A and B) or too strong (E) to permit the evolution of stable, non-zero frequencies of the female remodeling preference and the male autonomy-enhancing trait. The black dots are the initial frequency (*b*_2_ = *r*_2_ = 0.05). The lines’ colors show the frequency of the male attractive display trait (*t*_2_; initial freq. = 0.299). Other parameters are: *s*_*t*_ = 0.1, *s*_*b*_ = 0.1, *u* = 0.1, *s*_*r*_ = 0.01. See Supplementary Figure S5 for additional allele-frequency- and genetic-correlation-by-time data.

The expression for ${\hat{D}}_{BR}$ is more complex: if *γ* = 0, ${\hat{D}}_{BR}$ arises solely due to Fisherian sexual selection, and is increasing in *a*_*b*_. Indeed, Fisherian sexual selection comprises the leading (first-order) term for ${\hat{D}}_{BR}$ in which *γ* and *c* do not appear. For nonzero values of *γ,* graphical inspection suggests various ways that protectiveness of the autonomy-enhancing trait can (weakly) influence the association between R_2_ and B_2_. For example, for a given level of coercion *c*, lower values of *a*_*b*_ result in a monotonically decreasing relationship between ${\hat{D}}_{BR}$ and *γ*, whereas intermediate values yield a hump-shaped relationship, and finally larger values cause the relationship between ${\hat{D}}_{BR}$ and *γ* to be monotonically increasing (Supplementary Figure S4A). At low *a*_*b*_, increasing *γ* causes B_2_ males to lose mating opportunities relative to B_1_ males (as seen in the *β* term), but it additionally causes them to lose relatively more R_2_ females to their coercive competitors than they would otherwise, which disrupts the would-be correlation between R_2_ and B_2_. At higher *a*_*b*_, increasing *γ* instead augments ${\hat{D}}_{BR}$ because remodeling preference is strong enough that R_2_ females can take advantage of the indirect benefits of mating with B_2_ males and also of reliably mating with attractive T_2_ males, as indicated by the presence of *a*_*t*_ and t_2_ in the third-order term of the expression. By the same logic, increasing *c* for a given *a*_*b*_ affects the system such that it causes larger *γ* to possibly disrupt rather than augment ${\hat{D}}_{BR}$ (Supplementary Figure S4B). In all, female preference for the male autonomy-enhancing trait (and therefore the autonomy-enhancing trait itself) will persist if preference is sufficiently strong and *γ* is optimally large (the magnitude of $D_{BR}$ and $D_{TR}$ large enough), such that these forces overcome the negative effect of *γ* on the coefficient of direct selection on B_2_ and any direct negative viability selection (*s*_*r*_) on R_2_.

We can also see, as suggested above, that the evolution of female preference for the male autonomy-enhancing trait is indirectly affected by the correlation between the male display trait and autonomy-enhancing trait (${\hat{D}}_{TB}$) insofar as this term bolsters the initial evolution of B_2_ via indirect selection, to which *c* and *γ* both contribute positively.

Note that the expressions for the linkages, apart from ${\hat{D}}_{BR}$, only appear at second or third order because they arise from compounded interactions among coercion, female preference, and protectiveness. In reality, we expect many of these parameters to possibly be large. Thus, we must be careful in our interpretations of the first-order allele frequency recursion approximations. They should be taken as a simplified idea of the main dynamics, though they are missing higher-order features that come into play when selection is strong.

### Effect of key parameters on the evolution of the autonomy-enhancing trait

Naturally, stronger female preferences for attractive male display (*a*_*t*_) and for the autonomy-enhancing trait itself (*a*_*b*_) generally promote the evolution of the autonomy-enhancing trait (evolutionary equilibria where *b*_2_ > 0; Figure 3). The effectiveness of the autonomy-enhancing trait’s protection from coercion (*γ*) allows the evolutionary persistence of B_2_ as long as it is large enough, but also, for intermediate values of *a*_*b*_, not too large (Figures 3 and 4). For a given level of *a*_*b*_, the *γ* parameter must be large enough that sufficient genetic correlation builds up between the female remodeling preference and male attractive display trait (D_TR_; Figures 2C and 4), allowing the co-evolutionary feedback loop to overcome the costs, including viability costs to R_2_ females and B_2_ males, and loss of male sexual success through the reduced efficiency of sexual coercion. It is possible, however, for *γ* to be so large that although R_2_ females reliably attain the benefits of mating with attractive T_2_ males (D_TR_ is strong enough to overcome the costs of remodeling preference), males with the autonomy-enhancing trait do not receive enough mating benefits via the attraction of R_2_ females to outweigh the loss of coercive mating opportunities and the cost, *s*_*b*_, so B_2_ fails to increase and persist (Figure 4E). Because stronger female remodeling preferences give B_2_ males more of an advantage relative to B_1_ males, this effect is overcome in instances with higher values of *a*_*b*_ (Figure 3). In cases where the autonomy-enhancing trait persists at equilibrium, higher *γ* also leads to stronger oscillations as described above because R_1_ females are even more likely to have the autonomy to successfully choose an attractive T_2_ male when they encounter a protective B_2_ male by chance (Figure 4D). 

**Figure 5.**
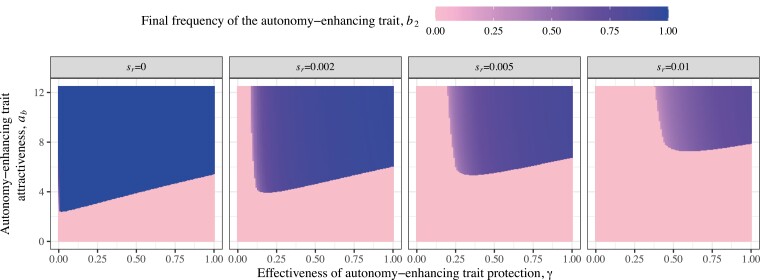
The effect of viability cost (*s*_*r*_) to female remodeling preference on the persistence of the male autonomy-enhancing trait B_2_ at equilibrium through parameter space for *a*_*b*_ and *γ*. In this example, *a*_*t*_ = 12, *c* = 0.4, *s*_*t*_ = 0.1, *s*_*b*_ = 0.1, and *u* = 0.1.

The viability cost of female remodeling preference (*s*_*r*_) also has an important effect on the evolution of the autonomy-enhancing trait. When *s*_*r*_ = 0, the minimum threshold for *γ* disappears because there is no longer a cost to overcome, and indeed even an ineffective autonomy-enhancing trait (*γ* = 0) and remodeling preference can evolve to persist at equilibrium simply due to arbitrary Fisherian sexual selection alone (Figure 5; Kirkpatrick, 1982; Pomiankowski et al., 1991; Prum, 2010). When *s*_*r*_ = 0, and *γ* > 0 (but not too large), B_2_ can evolve to fixation because there is no more “freeloading” effect: R_2_ females are able to outcompete R_1_ females even when B_2_ is at high frequency. Overall, higher *s*_*r*_ means higher threshold values of *γ* and *a*_*b*_ are required to build up enough indirect selection to support the stable evolution of female remodeling preference and therefore the persistence of the autonomy-enhancing trait (Figure 5).

Finally, somewhat counter-intuitively, a higher likelihood of successful coercion (larger *c*) shifts the minimum *γ* threshold lower for the same strength of remodeling preference (*a*_*b*_), and moreover allows for higher frequency of the male autonomy-enhancing trait at equilibrium where it evolves to persist (Figure 3). This is because higher likelihoods of successful coercion mean bigger rewards to females in terms of sexual autonomy for preferring the protective male trait—as we saw in the weak selection analysis, D_TR_ is increasing in *c*. At the same time, higher *c* results in higher *a*_*b*_ necessary to allow for the autonomy-enhancing trait to evolve because B_2_ males have more to lose in terms of coercive mating opportunities for a given level of protectiveness (*γ*). However, as indicated by the *β* expression in the weak selection approximation, *c* and *γ* both contribute (to leading order) to direct selection against B_2_ in the same way. Interestingly, this manifests such that lower *c* allows for higher possible magnitudes of *γ* for a given strength of female remodeling preference (Figure 3).

### Selection for more protective autonomy-enhancing traits

**Figure 6.**
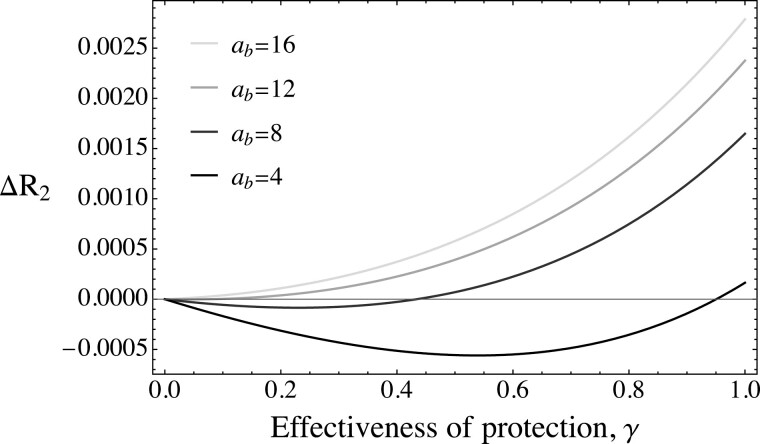
Examples of the change in frequency of the R_2_ allele across values for the attractiveness of the trait at the B locus (*a*_*b*_) and the effectiveness of the protection (*γ*) conferred by the male autonomy-enhancing trait, B_2_, for a version of the model where R_2_ females have a preference for protective autonomy-enhancing traits (B_2_ males), while R_1_ females have a preference of equal strength for nonprotective traits, and all else being equal (*r*_2_ = *b*_2_ = 0.5; *s*_*r*_ = *s*_*b*_ = 0; other parameters are: *a*_*t*_ = 5; *c* = 0.6, *s*_*t*_ = 0.1, *u* = 0.1). ΔR_2_ shown represents the change in the allele frequency in generation two (the first generation in which the R_2_ allele can evolve by indirect selection following the initial buildup of genetic correlations with the T and B loci).

**Figure 7.**
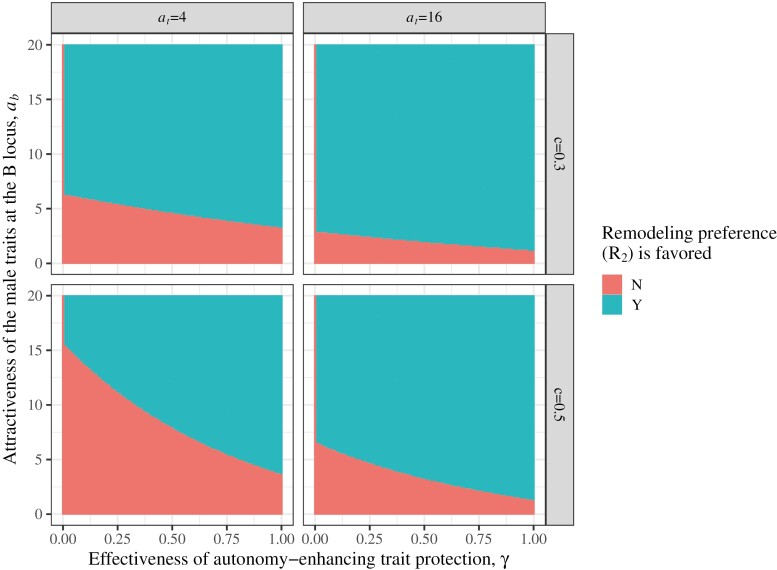
Analysis of the “invasion” of a female preference for protective autonomy-enhancing traits (the R_2_ allele) over a preference of equal strength for nonprotective traits, all else being equal (*r*_2_ = *b*_2_ = 0.5; *s*_*r*_ = *s*_*b*_ = 0), across values for the attractiveness of the trait at the B locus (*a*_*b*_), and the effectiveness of the protection (*γ*) conferred by the male autonomy-enhancing trait. Blue domains represent parameter sets where the R_2_ allele is initially favored (the R_2_ allele increases in generation two following the initial buildup of genetic correlations with the T and B loci). Red domains are where the R_2_ allele is not favored by selection. Parameter sets shown in this figure are: *a*_*t*_ = {4,16}, *c* = {0.3,0.5}, *a*_*b*_ = 0 to 20 at increments of 0.1, γ = 0 to 1 at increments of 0.01, *u* = 0.1, and *s*_*t*_ = 0.1.

Because of the additional indirect selection arising from increases in sexual autonomy, invading preferences for more effective autonomy-enhancing traits with higher *γ* will be subject to stronger positive selection than preferences of similar strength for less-protective autonomy-enhancing traits as long as the overall strength of female preference is great enough. We can demonstrate this by examining a version of the model in which R_2_ females prefer B_2_ males by a factor of (*a*_*b*_ + 1), but instead of randomly visiting territories, R_1_ females have an equally strong preference (also parameterized as *a*_*b*_ + 1) for non-autonomy-enhancing B_1_ males (see Supplementary Appendix S3 and Supplementary File S3). We set *s*_*r*_ and *s*_*b*_ to zero to compare invasion scenarios wherein the only difference is the value for *γ*. We start the population at *r*_2_ = 0.5 and *b*_2_ = 0.5 so that the effect of sexual selection will be equally strong on both the B_1_ and B_2_ alleles. We can then explore the influence of adding an autonomy-enhancing effect (*γ*) to B_2_ by asking whether the remodeling preference R_2_ increases as a result. Graphical and simulation analysis of the model recursion equations reveals that for a given value of *a*_*b*_, there is a threshold value of *γ* past which the remodeling preference will be favored, and the R_2_ allele will increase more with larger *γ* (Figure 6). For larger values of *a*_*b*_, the protectiveness threshold becomes lower until R_2_ is favored for all *γ* > 0 (Figures 6 and 7). Essentially, R_2_ will be favored as long as females have enough specificity of their preferences to allow the fitness advantage of mating more reliably with T_2_ males to overcome effect of the loss of coercive mating opportunities to B_2_ males. Therefore, given strong enough female preference, preferences for autonomy-enhancing traits with stronger *γ* will invade more readily than arbitrary, Fisherian preferences or preferences for comparatively less protective autonomy-enhancing traits. In addition, similar to the main model, lower likelihood of successful coercion (*c*) promotes the invasion of preferences for comparatively more protective autonomy-enhancing traits by shifting the threshold preference strength lower, especially for lower values of *γ* (Figure 7). Given these results and observations from the main model that, for a given level of *a*_*b*_, (a) higher coercion allows for more weakly protective traits to evolve and (b) lower coercion permits the evolution of more strongly protective traits (Figure 3), it is plausible that once conditions allow for autonomy-enhancing traits to persist in the population, incremental advances in coercion protection efficiency (resulting in lower effective coercion) may evolve into a “sexual autonomy snowball” that gradually dismantles sexual coercion.

## Discussion

We have demonstrated that a male “autonomy-enhancing” trait can plausibly evolve by female mate choice because it enhances female sexual autonomy, establishing a new evolutionary possibility for systems involving sexual conflict over the indirect benefits of mate choice. Rather than possibly engaging in an arms race by evolving direct resistance to male coercion, females may actively remodel male coercive capacity to expand their own sexual autonomy. This highlights the importance of allowing female reproductive traits greater complexity and biological realism than has been considered in some past theory on sexual conflict. As hypothesized, females that prefer to mate with males having the autonomy-enhancing trait can be at an advantage because they are protected from male coercion and thus more likely to mate with an attractive male (Figure 2D). The evolution of genetic correlation between the autonomy-enhancing trait, attractive display trait, and female “remodeling” preference for the autonomy-enhancing trait (Figure 2C) produces a co-evolutionary feedback loop that causes indirect selection on female remodeling preference. As long as the loss of coercive mating opportunities as a result of producing the protective autonomy-enhancing trait is not too large, this process enables the male autonomy-enhancing trait and the corresponding female preference to persist in the population because the force of sexual selection can overcome the costs to females and males in terms of viability (Figure 4). Our numerical simulations demonstrate that when faced with male coercion, females evolve preferences for male autonomy-enhancing traits that persist in the population through a significant portion of parameter space (Figure 3), increasing females’ ability to freely choose mates at equilibrium (Figures 2A and D and 4). We additionally showed that greater protectiveness conferred by the autonomy-enhancing trait (larger *γ*) often produces greater indirect selection on female remodeling preference, and thus autonomy-enhancing traits conferring more protection can often invade more readily than less protective traits, or nonprotective, arbitrarily attractive ones (Figures 6 and 7). Coupled with the observation that, for a given level of female remodeling preference, decreasing effective coercion allows for more strongly protective male traits persisting at equilibrium (Figure 3 and weak selection approximation), this suggests that the initial evolution of remodeling preferences and autonomy-enhancing traits can potentially lead to a sexual autonomy “snowball” effect of increasing protection. This dynamic may be offset, or more complex, under different cost structures, such as the cost of male autonomy-enhancing traits scaling with their protectiveness.

In the case where female preference for the autonomy-enhancing trait is costly to females (*s*_*r*_ > 0), we revealed oscillatory dynamics unanticipated by previous verbal formulations (Prum, 2015, 2017). The protection from coercion conferred by male autonomy-enhancing traits acts as a sexually dimorphic “public good,” allowing females in the population to potentially attain the benefits of expanded sexual autonomy without paying the costs of seeking it out. Intriguingly, this suggests that when investigating the remodeling process in nature, researchers could expect to see potentially large variation in female preference for autonomy-enhancing traits. In our model, the possibility of freeloading produced by viability costs to female remodeling preference also prevents the male autonomy-enhancing trait from going to fixation since whenever it approaches high frequency, the advantage to females for preferring the autonomy-enhancing trait diminishes in favor of females who do not pay any costs (e.g., Figure 2A). Further, although higher protectiveness allows for higher equilibrium frequency of the male autonomy-enhancing trait, it also causes stronger oscillations (Figure 4). Thus, observed variation in male strategies is also potentially consistent with an ongoing remodeling process in nature. However, we assumed a fixed cost to female remodeling preference; a different assumption such as costs that depend on the frequency of the preferred male trait (e.g., Kokko et al., 2015) would likely dampen these dynamics. Of course, whether there are differential viability costs to females preferring protective autonomy-enhancing traits as opposed to other attractive aspects of male phenotype is an empirical question.

It is likely that the remodeling process is more complex and possibly less constrained than suggested by the necessarily simple model presented here. For tractability, we assumed that all males would attempt coercion if given the opportunity. In a more complex model in which coercion were allowed to evolve, in cases when the selective environment shifts toward favoring the evolution of the autonomy-enhancing trait, we expect that the coercion strategy would become disfavored (Snow et al., 2019). This dynamic could promote the evolution and persistence of the protective autonomy-enhancing trait beyond the parameter space we observed under our relatively more restrictive assumptions (Figures 2–4). Interestingly, given the counter-intuitive result that higher likelihood of successful coercion can frequently produce greater selection for remodeling (since there are higher rewards for females that prefer the autonomy-enhancing trait; Figure 3), it is also possible that even more complex or cyclical dynamics would arise as coercion evolved to be rarer.

However, we also assumed a fixed female preference for the attractive male display trait allele. A more complicated model with an evolving locus for female preference for display (in addition to female remodeling preference) could possibly involve initially weaker indirect benefits of sexual autonomy via remodeling if the starting condition featured the female preference allele at an intermediate frequency. This is because sexual selection on the display trait would be weaker, so it would be less beneficial for females to visit males with autonomy-enhancing traits.

Evolving preference for display *and* an additional locus allowing for evolving coercion (heritable variation in coercive ability among males) would introduce the possibility of indirect benefits of coercion in terms of the coercive ability of sons (Holland & Rice, 1998; Kokko, 2005), even in the absence male autonomy-enhancing traits. This could hinder or promote the evolution of remodeling preferences under certain conditions, but it is challenging to predict, particularly if coercive males must pay a viability cost relative to noncoercive males. Regardless, the model presented here captures the dynamics of remodeling within the domain in which it is possible, i.e., in situations where coercion is not so strong that it overwhelms any benefits of display.

Finally, we assumed differential viability costs to males producing the autonomy-enhancing trait, but one can imagine alternative scenarios. For example, if male coercive behavior is aided by a costly morphological weapon, “remodeled” males with smaller or less functional weapons will be less able to coerce but may not pay differential costs or may even have higher viability. This “deweaponization” scenario (Prum, 2017) would likely promote the evolution of the autonomy-enhancing trait beyond what we have presented. Additionally, as male coercion becomes constrained by remodeling and females gain sexual autonomy, this may open the door to advances in other areas of sexual conflict along “axes” not treated by our model, such as parental investment (Prum, 2017).

Although our results suggest that the remodeling process is a plausible alternative to direct resistance, and that it may be self-reinforcing once initiated, our model also demonstrates and clarifies several reasons why the evolutionary dismantling of sexual coercion via remodeling is not inevitable or ubiquitous. First, there must be phenotypic variation available in males that incidentally provides protection to females, and under costly remodeling preferences the threshold level of protectiveness (*γ*) can be quite high. Furthermore, females must be sufficiently choosy for remodeled males to attain a sufficient mating advantage to overcome the costs of having a protective phenotype in terms of lost coercive opportunities. The initiation of a remodeling process and its specific form will also be highly dependent on the ecological, developmental, physiological, and behavioral details of a given system. Remodeling can occur when males can subvert female preference and coerce females without giving them the opportunity to evaluate a display, while females can exercise agency by evolving new preferences that act in a manner independent of coercion’s influence. Thus, remodeling is likely to be more constrained in situations where females are able to evaluate males’ display to some extent from afar. Conversely, in the case of remodeling preferences that rely on evaluation of autonomy-enhancing traits from a safe distance, females must have a perceptual system that can operate on such a scale. For example, in waterfowl, females could hypothetically evolve a remodeling alternative to direct genital resistance by developing a way to detect males with reduced/de-weaponized genitals before choosing to associate with them. However, this would hinge on factors such as the distribution of food resources allowing for females to realistically avoid getting close to coercive males.

Expansion of sexual autonomy via remodeling as an alternative to direct resistance not only has been implicated previously in bowerbirds (Borgia, 1995; Prum, 2015, 2017), but also in the evolution of reduced body size and reduced canine tooth dimorphism in early hominids (Prum, 2017). Sexual coercion and infanticide are the social norm in many modern Old World monkeys and apes (Muller & Wrangham, 2009). Rather than possibly engaging in arms-race dynamics by evolving direct defenses or simply acquiescing to all mating attempts, early female hominins could have used their available behavioral agency to associate preferentially with less violent mates of similar size and with smaller canines. One way they could have accomplished this is through the formation of coalitions with other females or male “friends” (Hare et al., 2012; Palombit, 2009), or through choosing which groups to join during social group fission or individual dispersal (Palombit, 2014). Although our model demonstrates that it can be difficult to disentangle the effects of remodeling from typical sexual selection, comparative data are consistent with selection for traits associated with remodeled hominin coercive capacity, including the reduced body size dimorphism, canine size, and aggression, with increased prosociality that we see in modern humans and recent ancestors (Gordon, 2006; Hare, 2017; Hare & Tomasello, 2005; Hare et al., 2012; Lieberman, 2011; Prum, 2017; Wrangham et al., 2006). Our model results also suggest that perhaps a small percentage of females succeeding in selecting for remodeled male traits in this way could have been enough to provide benefits to all females and promote further advances in sexual autonomy.

Remodeling of sexual coercion may be an unappreciated factor shaping the evolution of many courtship displays, particularly in polygynous systems where a female must approach a male on his territory in order to experience a courtship display performance directed specifically at her, or where the evaluation of the display or ornament is similarly spatial- or time-scale dependent. For example, review of the lek display behaviors of male manakins (Pipridae; Bostwick, 2000; Prum, 1990) shows that multiple displays in numerous genera include elements with the male oriented backwards, or even approaching the female in backwards orientation. Display repertoires of the *Pipra*, *Ceratopipra*, and *Machaeropterus* clade include a homologous backward slide element where the male assumes a bowed, lowered-head posture, and approaches the female backwards on the display perch with tiny, rapid steps. Species in the *Masius* and *Corapipo* clade perform various displays on mossy fallen logs or buttress roots; these assorted bowing, bill-pointing, and wings-shiver displays and postures are inevitably oriented backwards toward the female. Species in the genus *Chiroxiphia* perform a cooperative “cartwheel” display with two or more males successively fluttering in a circle above a perch, and sliding along the perch after landing. The display is performed with the male flights oriented *away* from the perched female. In all of these cases, the displaying males’ orientation and directions of movement suggest the absence of coercive opportunity. Similarly, in birds of paradise, male *Paradisaea rudolphi* perform elaborate courtship displays upside-down, and *Seleucidis melanoleuca* and *Ptiloris victoriae* perch on broken trunks, to be approached by the female from below (Frith & Beehler, 1998). Both orientations obviate the possibility of forced copulation. Thus, it is likely that remodeling male coercive potential through female choice has had a broad impact on the specific form of courtship displays in polygynous species.

This is the first quantitative exploration of the remodeling mechanism; now that we have begun to shed light on this novel mechanism by which females may expand their sexual autonomy as a result of sexual conflict over indirect benefits, we hope that our model results can help identify previously unappreciated examples in nature. In particular, beyond polygynous species, our results reveal how commonly observed multi-stage and/or multimodal mate preferences (e.g., Candolin, 2003; Uy & Safran, 2013) may function as part of a remodeling process, with implications for female sexual autonomy and the evolutionary trajectory of male ornaments and behaviors.

## Supplementary Material

qpad074_suppl_Supplementary_Material

## Data Availability

Computer code is provided in the electronic supporting information. Data archival location: Dryad Digital Repository (datadryad.org) (https://doi.org/10.5061/dryad.t1g1jwt6s).
