# Supplementary material for: Remodeling male coercion and the evolution of sexual autonomy by mate choice
Source: Evolution. 2023 Jun 1;77(7):1564–77. doi: 10.1093/evolut/qpad074 (PMC10309969; doi:10.1093/evolut/qpad074)

SUPPORTING INFORMATION FOR "REMODELING MALE COERCION AND THE EVOLUTION OF SEXUAL AUTONOMY BY MATE CHOICE"

APPENDICES S1, S2, & S3

S.S. SNOW & R.O. PRUM

APPENDIX S1: DEVELOPMENT OF THE MAIN MODEL RECURSION EQUATIONS

Here we develop the recursion equations of the main model presented in the text. For detailed, reproducible code, including specifications for parameter values and numerical simulations, see the Mathematica notebook in Supporting Information File S1.

Our model includes the influences of five traits: female preference for male display, male display, male propensity/ability to sexually coerce, female remodeling preference, and the male autonomy-enhancing trait. As described in the main text, we assume that both female preference for male display and the male coercion trait are fixed in the population, functionally yielding a three-locus system.

We employ autosomal, haploid genetics with loci having sex-linked expression. The three diallelic loci (T for male display, B for male autonomy-enhancing trait, and R for female remodeling preference; see table 1 for definitions) give eight possible genotypes, $T_1B_1R_1$, $T_2B_1R_1$, $T_1B_2R_1$, $T_2B_2R_1$, $T_1B_1R_2$, $T_2B_1R_2$, $T_1B_2R_2$, and $T_2B_2R_2$, which we will index here as G_1 through G_8.

Viability selection

In males:

Males carrying the T_2 allele express a costly display trait such that they have a probability of (1-s_t) of surviving until the mating season relative to a T_1 male. Males carrying the B_2 allele similarly pay a viability cost for producing the autonomy-enhancing trait such that they have a (1-s_b) chance of survival relative to a male with the B_1 allele. Accordingly, adapting the notation of Dhole *et al*. (2018), the frequency of a genotype G_i in males after viability selection is given by

$$g'_{i_m} = \frac{(1-\theta_i s_t)(1-\zeta_i s_b)g_{i_m}}{\sum_i (1-\theta_i s_t)(1-\zeta_i s_b)g_{i_m}}$$

where g_{i_m} is the frequency of genotype G_i in males before viability selection. If the allele at the T locus for G_i is T_2 (i is even), then $\theta_i = 1$, and $\theta_i = 0$ otherwise. If the allele at the B locus is B_2 (i *mod* 4 is 3 or 0), $\zeta_i = 1$, and $\zeta_i = 0$ otherwise. The prime notation is a reminder that the new frequency has been adjusted by viability selection; this will carry through the rest of the section.

In females:

Females carrying the R_2 allele for female remodeling preference pay a small differential viability cost such that R_2 females have a (1-s_r) chance of surviving to mate relative to R_1 females. The frequency of a genotype G_j in females after viability selection is given by

$$g'_{j_f} = \frac{(1 - \psi_j s_r) g_{j_f}}{\sum_j (1 - \psi_j s_r) g_{j_f}}$$

where g_{j_f} is the frequency of genotype G_j in females before viability selection. If the allele at the R locus is R_1 ($j < 5$), $\psi_j = 0$, and $\psi_j = 1$ otherwise.

Sexual selection

After viability selection, mating occurs according to the scheme laid out in figure 1 and in the frequencies summarized in table 2 in the main text. Females with the remodeling preference (the R_2 allele) prefer to visit the territories of males with the autonomy-enhancing trait (the B_2 allele) by a factor of (1+a_b). Once on a territory, the male may coerce them into mating without giving the female the opportunity to evaluate the male's display with a probability c. Without coercion, females prefer to mate with attractive T_2 males by a factor of (1+a_t). (Without coercion, if a_t=0, females are equally likely to accept or reject the male, i.e., the probability of accepting the male is 1/2). The male autonomy-enhancing trait, B_2, confers protection from coercion given by the parameter γ such that females that visit territories of B_2 males have an adjusted likelihood of coercion $c(1-\gamma)$.

We assume that females continue visiting territories until they ultimately choose a male or are coerced. The ultimate frequency of the various pairings between male and female genotypes can be derived as follows:

If the probabilities that a female with a particular genotype mates with a male of genotype i on her initial visit are given by the vector $p_1, p_2, \ldots, p_n$, let

$$q = 1 - \sum_i^n p_i$$

where q is the probability that a female goes unmated after the first round of visits. The ultimate frequency of a particular pairing P_i after all visits are done will be given by the geometric series

$$P_i = p_i + p_i q + p_i q^2 + \cdots$$

which simplifies to

$$P_i = \frac{p_i}{1 - q}.$$

Since all P_i are adjusted by the same normalization factor, then we know that the ultimate frequency ratio will be equal to the probability ratio for the 1-visit expressions for any number of visits, with the normalization factor being the sum of the 1-visit probabilities in the case of infinite visits.

Thus, the final frequencies of the genotype pairings will be the initial probabilities that a particular genotype of female mates with each type of male on her first visit, normalized by the sum of the initial probabilities for that female genotype.

Formally, the frequency of matings between male genotype *i* and female genotype *j* is given by:

$$M_{ij} = g'_{j_f} \frac{g'_{i_m} \frac{(\sigma_{ij} a_b + 1)}{z_b} \left(\begin{array}{c} c(1-\varepsilon_i \gamma) + \\ (1 - c(1-\varepsilon_i \gamma)) \left(\frac{\omega_i a_t + 1}{a_t + 2} \right) \end{array} \right)}{z}$$

where

$$z = \sum\nolimits_i g'_{i_m} \frac{(\sigma_{ij} a_b + 1)}{z_b} \left(\begin{array}{c} c(1-\varepsilon_i \gamma) + \\ (1 - c(1-\varepsilon_i \gamma)) \left(\frac{\omega_i a_t + 1}{a_t + 2} \right) \end{array} \right)$$

$$z_b = b'_{1_m} + (\rho_j a_b + 1) b'_{2_m}$$

and where b'_{1_m} and b'_{2_m} are the frequencies of the two B alleles within males after viability selection, such that $b'_{1_m} = g'_{1_m} + g'_{2_m} + g'_{5_m} + g'_{6_m}$ and $b'_{2_m} = g'_{3_m} + g'_{4_m} + g'_{7_m} + g'_{8_m}$.

If the male allele at the B locus for genotype G_i is B_1 (*i*=1, 2, 5 or 6), $\varepsilon_i = 0$. If the male allele at the B locus for G_i is B_2 (*i*=3, 4, 7 or 8), $\varepsilon_i = 1$.

If the male allele at the T locus for G_i is T_2 (*i* is even), then $\omega_i = 1$, and $\omega_i = 0$ otherwise.

If the female allele at the R locus for G_j is R_2 ($j > 4$), $\rho_j = 1$, and $\rho_j = 0$ otherwise.

Finally, if the male allele at the B locus for G_i is B_2 (*i*=3, 4, 7 or 8) ***and*** the female allele at the R locus for G_j is R_2 ($j > 4$), then $\sigma_{ij} = 1$, and $\sigma_{ij} = 0$ otherwise.

Note that the z_b terms ultimately cancel out, but we have left them in place here to preserve the interpretation of the numerator of the M_{ij} expression as the probability that a female of genotype G_j mates with a male of genotype G_i on her first territory visit.

For reference, the "*x*" terms used in the summary mating matrix in table 2 in the main text correspond to the male genotype frequencies after viability selection as follows:

$$x'_{11\cdot} = g'_{1_m} + g'_{5_m}$$

$$x'_{21\cdot} = g'_{2_m} + g'_{6_m}$$

$$x'_{12\cdot} = g'_{3_m} + g'_{7_m}$$

$$x'_{22\cdot} = g'_{4_m} + g'_{8_m}.$$

Similarly, the "*y*" terms representing the frequencies of the two R alleles within females after viability selection used in table 2 correspond to the female genotype frequencies after selection such that:

$$y'_{\cdot\cdot 1} = g'_{1_f} + g'_{2_f} + g'_{3_f} + g'_{4_f}$$

$$y'_{\cdot\cdot 2} = g'_{5_f} + g'_{6_f} + g'_{7_f} + g'_{8_f}.$$

Recombination and mutation

After viability selection and mating, there is standard free recombination (r=1/2), yielding the zygote genotypes. Finally, we use biased mutation in our model as a simple mechanism for the purpose of maintaining diversity at the male display locus, T. Every generation, a portion *u* of the genotypes having the T_2 allele mutates into the corresponding genotype having the T_1 allele. Again adapting the notation of Dhole *et al.* (2018), the frequency of a genotype after mutation is therefore given by

$$g_{i_u} = g_i + \theta_i g_j u - (1 - \theta_i) g_i u$$

where $\theta_i = 0$ if the allele at the T locus is T_2 (i is even), and $\theta_i = 1$ otherwise, and $j = i + 1$.

With the expressions for genotype frequencies after viability selection, mating, recombination and mutation each generation in hand, we can generate the recursion equations for the genotype frequencies used in our numerical simulations and other analyses (e.g., $\Delta g_i = g_{i_{\tau+1}} - g_{i_\tau}$, where $g_{i_{\tau+1}}$ is the frequency of genotype G_i after one round of selection, mating, etc.). For analysis and visualization, we transformed these eight recursion equations into the equivalent but more easily interpretable seven equations for the allele frequencies at the three loci and first- and second-order linkage disequilibria (Bennett 1952).

APPENDIX S2: DERIVATION OF INITIAL FREQUENCY FOR THE T_2 ALLELE FOR SIMULATIONS, AND RELATED PARAMETER CONSTRAINTS

We extended the Kirkpatrick (1982) two-locus sexual selection model to include sexually coercive male behavior (*c*) and mutation bias at the male display trait locus (T) and mimicking the female visitation scheme we use in the main model. We analytically solve the model for the case where the female preference allele, P_2, is equal to one. This yields an expression for a stable equilibrium for T_2 between zero and one that represents a balance of female preference, male

display, male coercive behavior, viability selection, and biased mutation. We use this to calculate the starting frequency for T_2 in the full model. The equilibrium expression also dictates the minimum value for the a_t parameter such that these conditions still hold for our simulations. Finally, we can use the equilibrium expression to calculate the initial adjusted realized magnitude of a_t for a given level of coercion.

Reproducible code for this model and analysis can be found in the Mathematica notebook in Supporting Information File S2.

The model includes two diallelic loci (P for female preference, and T for male display trait), yielding four possible genotypes: T_1P_1, T_1P_2, T_2P_1, and T_2P_2 which we will index here as G_1 through G_4.

Viability Selection

Just as in the main model, males carrying the T_2 allele express a costly display trait that decreases their relative survival to reproduction by a factor of $(1-s_t)$. Thus, the frequency of a genotype G_i in males after viability selection is given by

$$g'_{i_m} = \frac{(1-\theta_i s_t) g_{i_m}}{\sum_i (1-\theta_i s_t) g_{i_m}}$$

where g_{i_m} is the frequency of genotype G_i in males before viability selection. If the allele at the T locus for G_i is T_2 ($i > 2$), then $\theta_i = 1$, and $\theta_i = 0$ otherwise.

Sexual Selection

Without coercion, P_1 female mate randomly with respect to the male T allele, and P_2 females prefer to mate with attractive T_2 males by a factor of $(1+a_t)$ (Note that this formulation differs slightly from the preference rule in Kirkpatrick 1982; our parameterization yields random mating at a_t=0). Just as in the main model, we add the effect of male coercive behavior such that a male may coerce a female that visits them into mating without giving the female the opportunity to evaluate the male's display with a probability c. The frequency of matings between male genotype i and female genotype j is given by:

$$M_{ij} = g'_{j_f} \frac{g'_{i_m}\left(c + (1-c)\left(\frac{\omega_{ij} a_t + 1}{\phi_j a_t + 2}\right)\right)}{z}$$

where

$$z = \sum_i g'_{i_m}\left(c + (1-c)\left(\frac{\omega_{ij} a_t + 1}{\phi_j a_t + 2}\right)\right).$$

If the allele at the female P locus for G_j is P_2 (j = 2 or 4), then $\phi_j = 1$, and $\phi_j = 0$ otherwise. If the allele at the male T locus for G_i is T_2 (i>2) ***and*** the allele at the female P locus for G_j is P_2 (j = 2 or 4), then $\omega_{ij} = 1$, and $\omega_{ij} = 0$ otherwise.

Recombination and mutation

After viability selection and assortative mating, free recombination follows zygote formation to yield new genotype frequencies, to which biased mutation is applied in exactly the manner described in section S1.1 for the main model.

Identification and stability of the intermediate equilibrium for the T_2 allele

From our expressions for per-generation change in genotype frequencies, we can generate recursion equations for allele frequencies t_2 and p_2 and for the linkage disequilibrium D_{TP} (see the Mathematica notebook in Supporting Information File S2 for the full recursion equations).

Assuming female preference for display is fixed ($p_2 = 1$ and $D_{TP} = 0$), the recursion equation for the frequency of the T_2 allele becomes

$$\Delta t_2 = \frac{t_2\begin{pmatrix} a_t(-(u+1)t_2(c+s_t-1)+cu+c-us_t+u+s_t-1) \\ -(c+1)\big(u(s_t t_2+s_t-2)+s_t(t_2-1)\big) \end{pmatrix}}{2t_2(a_t(c+s_t-1)+cs_t+s_t)-2(a_t c+c+1)}.$$

Setting $\Delta t_2 = 0$ and solving yields two equilibrium solutions, one at zero and one non-zero solution. The non-zero equilibrium expression for t_2 (Eq. 1 in the main text) is

$$\hat{t}_2 = \frac{1-u+\dfrac{2(1+c+a_t c)u}{s_t+cs_t+a_t(c+s_t-1)}}{1+u}.$$

By differentiating the expression for Δt_2 with respect to t_2 and then setting $t_2 = \hat{t}_2$ and rearranging, it can be shown that the equilibrium with an intermediate frequency at $\hat{t}_2$ will be stable (the sign of the expression will be negative) given that $0 < s_t < 1$, and $0 < u < 1$, as long as

$$a_t > \frac{1+u}{(u-1)(s_t-1)} - 1$$

and

$$0 < c < \frac{a_t - 2u - a_t u + (1+a_t)(u-1)s_t}{a_t + 2u + a_t u + s_t - us_t}$$

Thus, in our numerical simulations (presented in detail in the Mathematica notebook in Supporting Information File S1, and the results of which we report in the main text), we always chose sets of parameters such that the values of a_t and c were consistent with (large enough and small enough, respectively) the stability of $t_2 = \hat{t}_2$ in the absence of remodeling. This ensures a scenario at the beginning of our simulations wherein there is an initial balance between coercion and display (and selection and mutation) with females still realizing their mate preference to some extent despite the effect of coercion.

Initial adjusted realized magnitude of female preference

We can visualize the overall effect of coercion by asking what adjusted value of a_t would produce the equivalent equilibrium value of $\hat{t}_2$ if coercion were absent.

Evaluating $\hat{t}_2$ at c=0 and $a_t = a_{t_{adj}}$ yields:

$$\hat{t}_2|_{\{c=0,\, a_t=a_{t_{adj}}\}} = \frac{1-u+\dfrac{2u}{a_{t_{adj}}(s_t-1)+s_t}}{1+u}$$

We can then solve the equation

$$\hat{t}_2 = \hat{t}_2|_{\{c=0,\, a_t=a_{t_{adj}}\}}$$

to get the expression

$$a_{t_{adj}} = \frac{a_t(1-c)}{c(a_t+1)+1}$$

This is the same as the expression in Eq. 2 in the main text. The expression for $a_{t_{adj}}$ holds as along as the conditions outlined above for the stable initial non-zero T_2 equilibrium are met. See the Mathematica notebook in Supporting Information File S2 for the full calculations.

<u>APPENDIX S3: SPECIFICATION OF THE MODEL EXAMINING SELECTION FOR MORE PROTECTIVE AUTONOMY-ENHANCING TRAITS</u>

This version of the model, referenced in the subsection "*Selection for more protective autonomy-enhancing traits*" in the main text is nearly identical to the main model presented in the paper and above in Appendix S1 (the schemes and parameterizations for viability selection, recombination, and mutation are the same) but it employs an altered mating matrix. We use this as a complementary approach to parse the effects of "Fisherian" sexual selection and the remodeling process and to explore the force of selection on protective autonomy-enhancing traits as compared to other, less protective, or simply arbitrarily attractive traits for which females have

similarly strong preferences. Therefore, in this case, R_2 females prefer B_2 males by a factor of $(1+a_b)$, but, rather than visit territories randomly, R_1 females have an equally strong preference for (non-protective) B_1 males, such that their territory visits are biased towards B_1 males also by the same factor $(1+a_b)$.

All females still prefer T_2 males by a factor of $(1+a_t)$. Finally, male coercive behavior and protection from coercion work as in the main model, with all males having the ability to coerce (with a likelihood of success c) and only B_2 males having the possibility of producing a trait that protects against coercion with an effectiveness γ. Apart from the mating biased toward B_1 males associated with R_1 females, mating takes place by exactly the same process as described above and in Figure 1.

Thus, using the same indices for genotypes in males and females as above, the frequency of matings between male genotype i and female genotype j is given by:

$$M_{ij} = g'_{j_f} \frac{g'_{i_m} \frac{(\sigma_{ij} a_b + 1)(\xi_{ij} a_b + 1)}{z_b} \begin{pmatrix} c(1-\varepsilon_i \gamma) + \\ (1 - c(1-\varepsilon_i \gamma)) \left(\frac{\omega_i a_t + 1}{a_t + 2}\right) \end{pmatrix}}{z}$$

where

$$z = \sum_i g'_{i_m} \frac{(\sigma_{ij} a_b + 1)(\xi_{ij} a_b + 1)}{z_b} \begin{pmatrix} c(1-\varepsilon_i \gamma) + \\ (1 - c(1-\varepsilon_i \gamma)) \left(\frac{\omega_i a_t + 1}{a_t + 2}\right) \end{pmatrix}$$

$$z_b = \left((1-\rho_j) a_b + 1\right) b'_{1_m} + \left(\rho_j a_b + 1\right) b'_{2_m}$$

and where b'_{1_m} and b'_{2_m} are the frequencies of the two B alleles within males after viability selection, such that $b'_{1_m} = g'_{1_m} + g'_{2_m} + g'_{5_m} + g'_{6_m}$ and $b'_{2_m} = g'_{3_m} + g'_{4_m} + g'_{7_m} + g'_{8_m}$.

If the male allele at the B locus for genotype G_i is B_1 (i=1, 2, 5 or 6), $\varepsilon_i = 0$. If the male allele at the B locus for G_i is B_2 (i=3, 4, 7 or 8), $\varepsilon_i = 1$.

If the male allele at the T locus for G_i is T_2 (i is even), then $\omega_i = 1$, and $\omega_i = 0$ otherwise.

If the female allele at the R locus for G_j is R_2 ($j > 4$), $\rho_j = 1$, and $\rho_j = 0$ otherwise. In this version of the model, this shifts the mating bias from B_2 to B_1 males rather than simply turning the bias on and off.

Finally, if the male allele at the B locus for G_i is B_2 (i=3, 4, 7 or 8) ***and*** the female allele at the R locus for G_j is R_2 ($j > 4$), then $\sigma_{ij} = 1$, and $\sigma_{ij} = 0$ otherwise.

Similarly, we now have another term such if the male allele at the B locus for G_i is B_1 (i=1, 2, 5 or 6) ***and*** the female allele at the R locus for G_j is R_1 ($j < 4$), then $\xi_{ij} = 1$, and $\xi_{ij} = 0$ otherwise.

Note that again the z_b terms ultimately cancel out, but we have left them in place to preserve the interpretation of the numerator of the M_{ij} expression as the probability that a female of genotype G_j mates with a male of genotype G_i on her first territory visit.

For analysis and visualization, after recombination and mutation we again transformed the eight recursion equations into the equivalent seven equations for the allele frequencies at the three loci and first- and second-order linkage disequilibria (Bennett 1952).

To compare scenarios where the only difference in traits is the value of γ, we set s_r and s_b to zero. We started the population with r_2 and b_2 at 0.5 to control for frequency-dependent effects on the strength of sexual selection and started t_2 at the initial equilibrium frequency described in Appendix S2. We then calculated the magnitude of ΔR_2 in the second generation following the buildup of genetic correlations to assess for which values of γ (and other parameters) the female remodeling preference would be favored.

Reproducible code for this model and analysis can be found in the Mathematica notebook in Supporting Information File S3.

Literature Cited

Dhole S, Stern CA, Servedio MR. 2018 Direct detection of male quality can facilitate the evolution of female choosiness and indicators of good genes: Evolution across a continuum of indicator mechanisms. *Evolution* **72**(4), 770-784. (doi:10.1111/evo.13466).

Bennett JH. 1952 On the theory of random mating. *Annals of eugenics* **18**(4), 311-317. (doi: 10.1111/j.1469-1809.1952.tb02522.x).

Supporting Information for "Remodeling Male Coercion and the Evolution of Sexual Autonomy by Mate Choice"

Supplemental Figures S1-S5

S.S. Snow & R.O. Prum

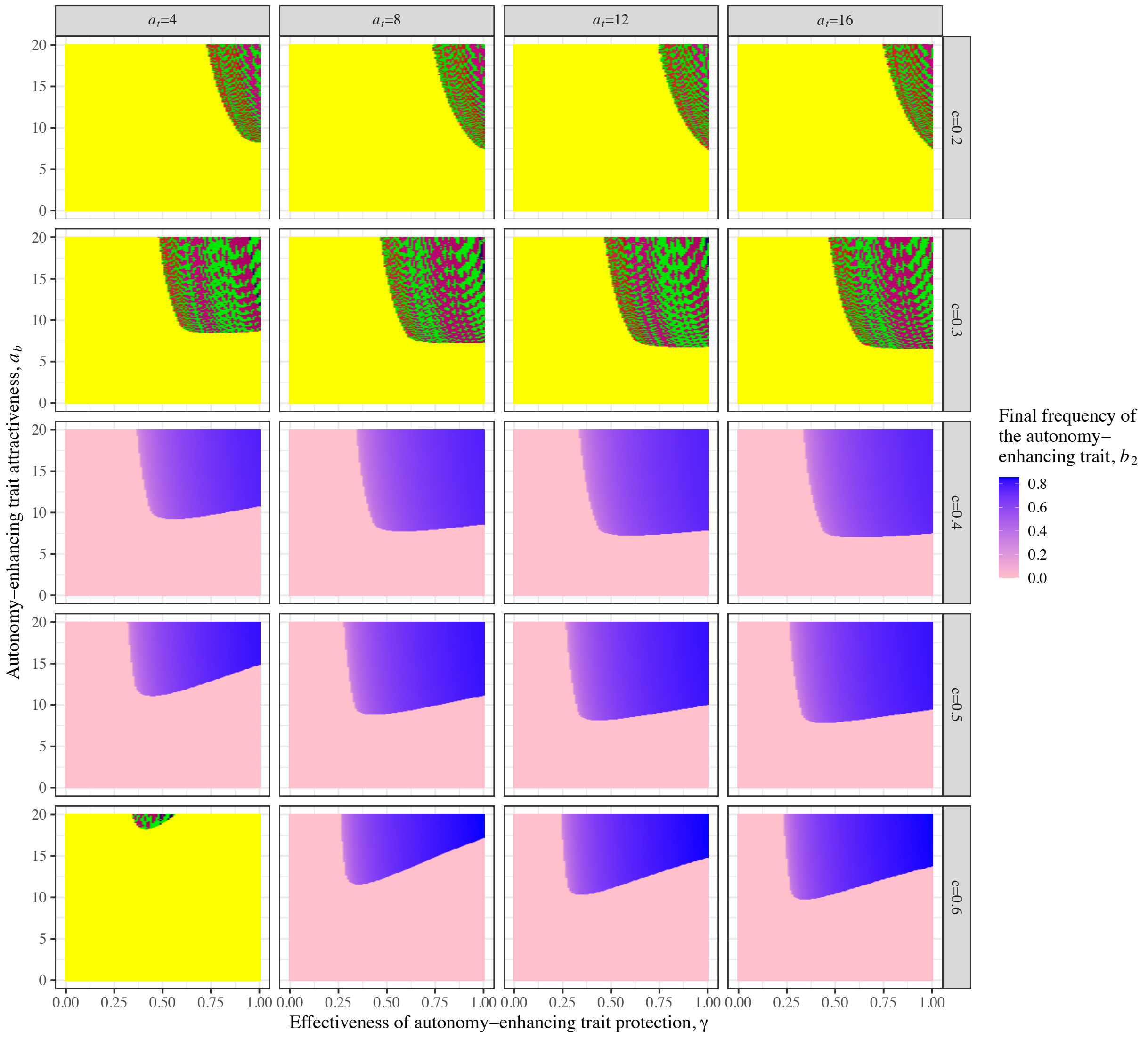

Figure S1. Results of numerical simulations of the model for the persistence of the male autonomy-enhancing trait (B_2) through the full parameter space explored for this study. Color represents the final frequency of the B_2 allele at equilibrium. Parameter sets are: a_t={4, 8, 12, 16}, c={0.2, 0.3, 0.4, 0.5, 0.6}, s_t=0.1, s_b=0.1, u=0.1, s_r=0.01, a_b=0 to 20 at increments of 0.1, and γ=0 to 1 at increments of 0.01.

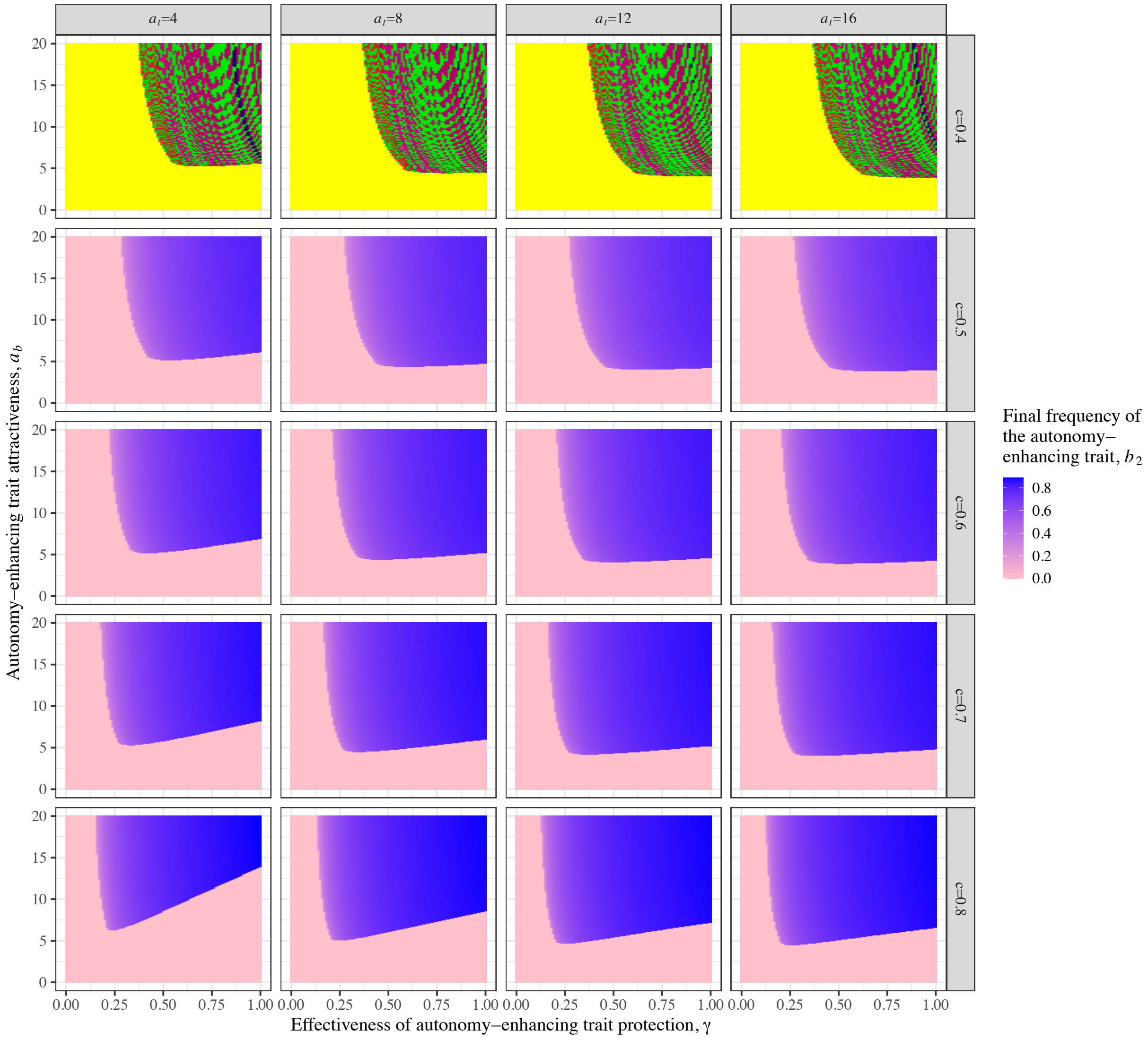

Figure S2. Results of low-mutation (u=0.01) version of numerical simulations of the model for the persistence of the male autonomy-enhancing trait (B_2). Color represents the final frequency of the B_2 allele at equilibrium. Parameter sets are: a_t={4, 8, 12, 16}, c={0.4, 0.5, 0.6, 0.7, 0.8}, s_t=0.1, s_b=0.1, s_r=0.001, a_b=0 to 20 at increments of 0.1, and γ=0 to 1 at increments of 0.01. Note that the lower mutation rate alters the window for possible parameter ranges for our simulations (see Appendix S2), so the range of values for c explored differ somewhat from Figure S1.

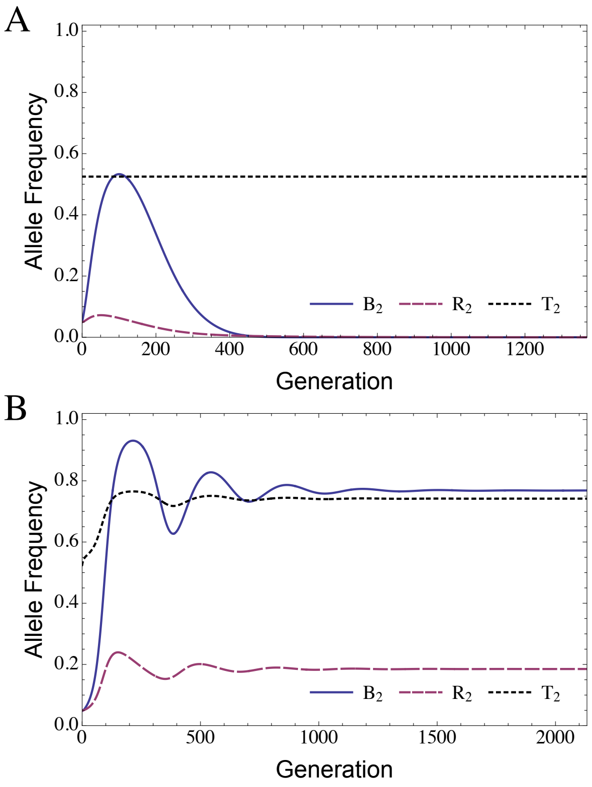

Figure S3. Allele-frequency-by-generation-time representations of the numerical simulation data presented in Figure 2A. (A) Results for the γ=0 scenario. (B) Results for the γ=0.8 scenario.

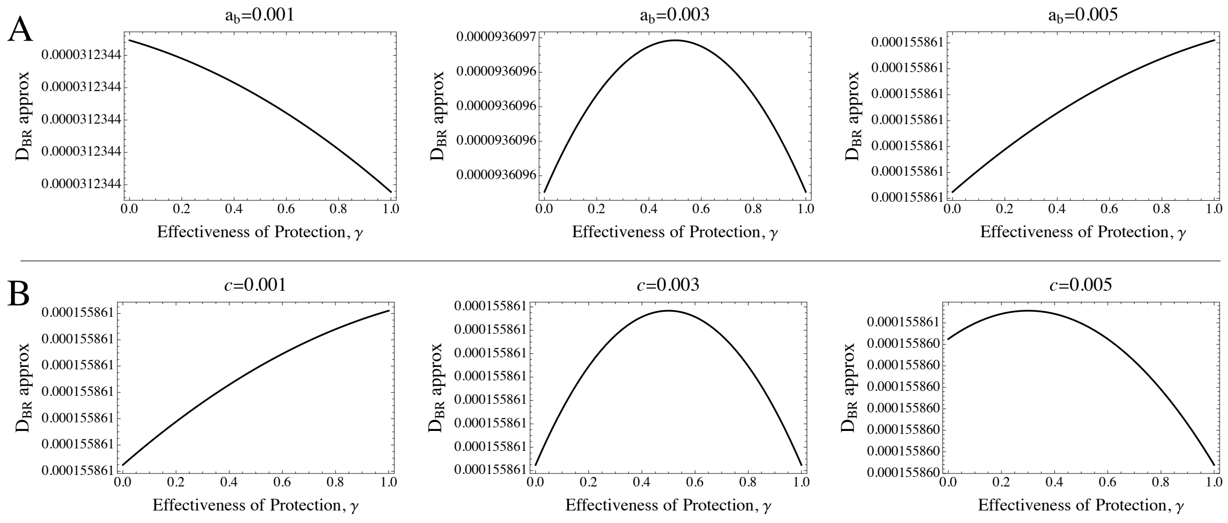

Figure S4. Relationship between the approximate magnitude of the genetic correlation between the B_2 and R_2 alleles (D_{BR}) at quasi-linkage equilibrium and the magnitude of the effectiveness of the protection associated with the male autonomy-enhancing trait (γ) across values of the attractiveness of the male autonomy-enhancing trait (a_b; panel A), and across values of the likelihood of successful coercion (c, panel B). Approximation derived from our weak selection approximation analysis (File S4). Other parameter values for panel A are: c=0.001, a_t=0.002, s_t=0.001, s_b=0.001, u=0.0001, s_r=0.00001, r_2=b_2=0.5. Parameter values for panel B are the same except a_b is held constant at 0.005. In each instance, $t_2=\hat{t}_2$, as dictated by the combination of the other parameters (See Appendix S2 and File S4).

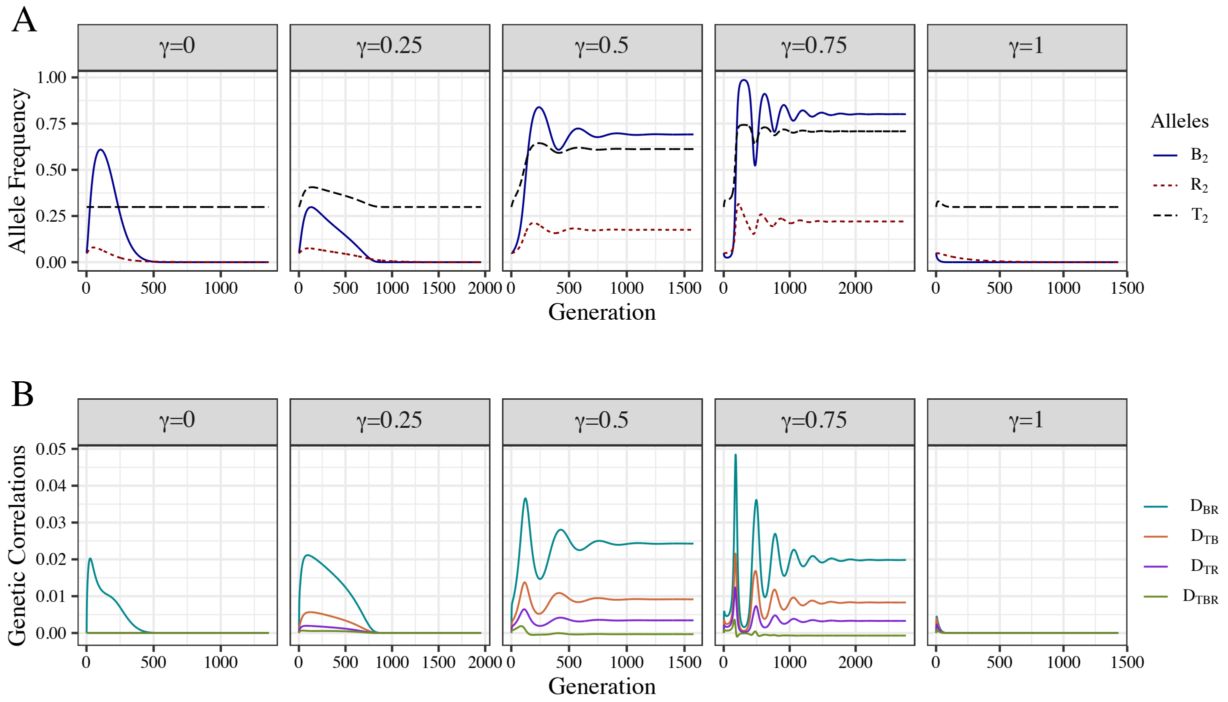

Figure S5. Supplemental data associated with the numerical simulation results presented in Figure 4. (A) Allele-frequency-by-generation-time representations data presented in Figure 4. (B) Corresponding genetic-correlation-by-generation-time results for the same numerical simulations. Note that the x-axis limits for generation times have variable scales.

Supporting Information File S1

Mathematica code for: Remodeling and the Evolution of Sexual Autonomy by Mate Choice

Samuel S. Snow[1,2*] and Richard O. Prum[2]
*Corresponding author: samuel.s.snow@gmail.com

[1]Université Toulouse 1 Capitole and Institute for Advanced Studies in Toulouse (IAST), Toulouse, France

[2]Department of Ecology and Evolutionary Biology, Yale University, New Haven, Connecticut, 06511

This code builds the model equations and performs a numerical projection of the three–locus population–genetic model presented in the main text.

See Table 1 for definitions of alleles and key parameters.

*Note on parametrization: in the main text and Appendices S1–S3, we refer to the mutation parameter as "*u*" to avoid confusion with the subscript m used to denote male genotype frequencies. In the code below, the mutation parameter is denoted "m" and has been left as such to avoid the introduction of errors.

```
ClearAll[st, sb, at, c, γ, m, init, T1B1R1, T2B1R1, T1B2R1, T2B2R1, T1B1R2, T2B1R2,
  T1B2R2, T2B2R2, genotypes, r, msel, fsel, Output, u, ab, z1, z2, zb, T2, B2,
  R2, DTB, DTR, DBR, DTBR, Mating, s, mgenotypes, T21, B21, R21, DTB1, DTR1,
  DBR1, DTBR1, ΔT2, ΔB2, ΔR2, ΔDTB, ΔDTR, ΔDBR, ΔDTBR, perturb, sr, materec,
  RecTable, MaskProb, ZygoteGenotypes, prob, zyg1, zyg2, zygoteProb, loop];

(*three-locus popgen simulation of
  Remodeling without direct costs to coercive mating*)

(*this model uses a probabilistic formulation for how
 females visit and mate at male territories. Females may either
 visit territories randomly or choose territories based on
```

```
 the kind of remodeling trait (bower) they see. Once there,
the male may either coerce them into mating there without the
 opportunity to evaluate their display, or the female may choose
 the mate with that male (given that he has not already coerced her)
 by some factor related to his attractivenes.*)

(*There is a locus for male display trait T2/T1,
    remodeling trait (protective bower) B2/B1,
    and female preference for the type of Remodeling trait (bower),
    R2/R1. We make two underlying assumptions in this model: 1)
   that all males will coerce if they have the opportunity, and 2)
 that all females have a preference for attractive T2 males*)

(*parameters:
 (1-st) is the fraction by which males having the T2 allele survive relative
  to Males having the T1 allele. (1-sb) is the fraction by which males
  having the B2 allele survive relative to Males having the B1 allele.
   (1-sr) is the fraction by which females having the
  R2 allele survive relative to females having the R1 allele.
   (a+1) is the factor by which attractive T2 allele males
  have a relative advantage in mating success over T1 males.
   (ab+1) is the factor by which
  females with the R2 allele prefer to visit B2 bowers.
   c is a number between 0 and 1 representing
  the rate of successful forced fertilization of a female,
(or the likelihood of coercive attack) by a male
  without a protective bower (B2).
   γ is a number between 0 and 1 representing the effectiveness of
  protective bowers at reducing a male's ability to coerce. If γ=0,
then there is no effect, and if γ=1, then B2 males are unable to coerce,
and females are free to choose the male based
 on his attractiveness once they are at his territory.

  Lastly, we make an assumption of biased mutation at the T locus. This
 is meant as a proxy for a quantitative, multialleleic trait or
 some other process by which variation is maintained at the T locus,
so that sexual selection is ongoing and we can explore the effects of sexual
  conflict over indirect effects in a simple population-genetic framework*)

(*the equilibrium conditions for a non-zero T2 in the absence of B and R
  are: 0<st<1&&0<m<1&&at>-1+(1+m)/((-1+m) (-1+st))&&0<c<(at-2 m-at m-st-at st+m st+at m st)/(at+2 m+at m+st-m st). See
      Appendix S2 and Supporting Information File S2 for derivation*)

(*use these conditions to determine appropriate parameter values*)
```

$$\text{Manipulate}\left[\left\{-1+\frac{1+m}{(-1+m)\ (-1+st)}\ ,\ \frac{at-2\,m-at\,m+(1+at)\ (-1+m)\ st}{at+2\,m+at\,m+st-m\,st}\right\},\right.$$

```
{{m, 0.1}, 0, 1}, {{st, 0.1}, 0, 1}, {{at, 4}, -1 + (1 + m)/((-1 + m) (-1 + st)), 30}];
```

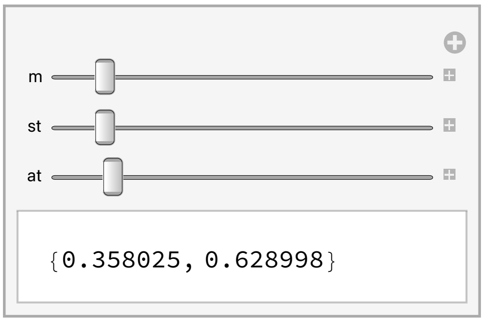

;

```
(*set parameters*)

at = 12;
ab = 12;
c = 0.5;
γ = 0.8;
st = 0.1;
sb = 0.1;
sr = 0.01;

(*recombination and mutation rate*)
rtb = 1 / 2;
rbr = 1 / 2;
m = 0.1;

(*The initial frequency for T2 is the equilibrium value for when
 B2 and R2 equal zero. This is where female preference for T2,
mutation bias, and male coercion are in balance. See Appendix
 S2 and Supporting Information File S2 for derivation*)

Teq = (1 - m + (2 (1+c+at c) m)/(st+c st+at (-1+c+st)))/(1 + m);

(*In support of understanding the effect of the presence of R2/B2 on female
 sexual autonomy: we want to know the proportion of T2 males females
   get at equilibrium in the initial state as compared to when females
   hypothetically have full autonomy (when c=0). See Supporting Information
   File S2 for derivation. Throughout the model runs we calculate the
   proportion of matings with T2 males for R1 and R2 females to give
   insight into the process and to get a metric of autonomy at the end.*)
```

```
t2propeq = 1 - (2 (1 + c + at c) m)/((-1 + m) (st + c st + at (-1 + c + st)));

(*same proportion but evaluated at c=0*)

t2propaut = 1 - (2 m)/((-1 + m) (at (-1 + st) + st));



(*inital genotype frequecies. We can look at how the
 introduction of the B2 abnd R2 alleles perturbs the system. *)

T2 = Teq;
B2 = 0.05;
R2 = 0.05;
DTB = 0;
DTR = 0;
DBR = 0;
DTBR = 0;

init = Solve[{T1B1R1 + T2B1R1 + T1B2R1 + T2B2R1 + T1B1R2 + T2B1R2 + T1B2R2 + T2B2R2 == 1,
    T2 == T2B1R1 + T2B2R1 + T2B1R2 + T2B2R2, B2 == T1B2R1 + T2B2R1 + T1B2R2 + T2B2R2,
    R2 == T1B1R2 + T2B1R2 + T1B2R2 + T2B2R2, DTB == (T2B2R1 + T2B2R2) - T2 * B2,
    DTR == (T2B1R2 + T2B2R2) - T2 * R2, DBR == (T1B2R2 + T2B2R2) - B2 * R2,
    DTBR == T2B2R2 - T2 * ((T1B2R2 + T2B2R2) - B2 * R2) -
      B2 * ((T2B1R2 + T2B2R2) - T2 * R2) - R2 * ((T2B2R1 + T2B2R2) - T2 * B2) - T2 * B2 * R2},
   {T1B1R1, T2B1R1, T1B2R1, T2B2R1, T1B1R2, T2B1R2, T1B2R2, T2B2R2}];

T1B1R1 = T1B1R1 /. init;
T2B1R1 = T2B1R1 /. init;
T1B2R1 = T1B2R1 /. init;
T2B2R1 = T2B2R1 /. init;
T1B1R2 = T1B1R2 /. init;
T2B1R2 = T2B1R2  /. init;
T1B2R2 = T1B2R2 /. init;
T2B2R2 = T2B2R2 /. init;


(*genotypes vector with initial allele frequencies and linkages*)
genotypes = Flatten[{T1B1R1, T2B1R1, T1B2R1, T2B2R1,
    T1B1R2, T2B1R2, T1B2R2, T2B2R2, T2, B2, R2, DTB, DTR, DBR, DTBR}];

(*set up the table of recombination probabilities that will yield the
 distribution of zygote genotypes resulting from each mated pairing*)
```

```
Nloci = 3;
Geno = 2^Nloci;
masklim = 2^(Nloci - 1);
recrate[1] = rtb;
recrate[2] = rbr;

(*Maskprob gives the probability for a particular pattern of
 recombination (a mask),based on given values of recombination
 rates between different loci ("recrate" vector above)*)

MaskProb = Function[{mask, Nloci},
   For[prob = 1; z = 1, z < Nloci, z++,
    If[BitGet[mask, z] == BitGet[mask, z - 1],
     prob *= (1 - recrate[z]),
     prob *= recrate[z]]]];

(*generates the two zygote genotypes (zyg1 and zyg2)
 from two parental genotypes (i and j) for a given mask.*)

ZygoteGenotypes = Function[{i, j, mask},
   For[zyg1 = 0; zyg2 = 0; k = 0, k < Nloci, k++,
    If[BitGet[mask, k] == 0,
     zyg1 = BitOr[zyg1, (BitAnd[i, (2^k)])];
     zyg2 = BitOr[zyg2, (BitAnd[j, (2^k)])],
     zyg1 = BitOr[zyg1, (BitAnd[j, (2^k)])];
     zyg2 = BitOr[zyg2, (BitAnd[i, (2^k)])]]]];

(*Produce a 3-dimentional matrix with the cells representing
  the probability of producing a zygote genotype (k) from parental
  genotypes i (mother) and j (father). Multiplying this matrix with
  the mating table that gives the proportions of different male-
 female genotype mating combinations will give the
  frequency of zygote genotypes in the new generation
  (after summing across parental genotypes).*)

RecTable = Table[0, {i, Geno}, {j, Geno}, {k, Geno}];
For[i = 0, i < Geno, i++,
 For[j = 0, j < Geno, j++,
  For[mask = 0, mask < masklim, mask++,
   MaskProb[mask, Nloci];
   zygoteProb = (1 / 2) * prob;
   ZygoteGenotypes[i, j, mask];
   RecTable[[(i + 1), (j + 1), (zyg1 + 1)]] += zygoteProb;
   RecTable[[(i + 1), (j + 1), (zyg2 + 1)]] += zygoteProb;]]]

(*uncomment this line to view the recombination table*)
(*MatrixForm[RecTable]*)
```

```
Output = {{1, 2, 3, 4, 5, 6, 7, 8, 9, 10, 11, 12, 13, 14, 15, 16, 17, 18},
   Flatten[{genotypes, 0, 0}]};

loop = 0;

(*Uncommenting the following two lines and the section indicated below will
 add a random perturbation to the system when it reaches euilibrium the
 first time and then continue the simulation until it settles on equilibrium
 again. This was done in our numeric simulations through parameter space for
 Figure 3 and Figure S2.1 to avoid slow convergence on local optima. Since
 the system always returns to nearly identical equilibrium values
 following perturbation for the parameter set used to produce Figure 2,
the perturbation was omitted in that case for aesthetic purposes*)

(*perturb=0;
(Label[start];*)


(*begin the generation loop. The model will iterate until the genotype
 frequencies and linkages are constant to seven decimal places*)

While[
  SetAccuracy[ Last[Output], 7] ≠ SetAccuracy[Output⟦(Length[Output] - 1)⟧, 7],

  (*make a vector that will perform Natural Selection on males,
  affecting gene frequencies before mating.*)

  msel = Table[i, {i, 1, 8}];
  u = Total[genotypes⟦{1, 5}⟧] + (1 - st) * Total[genotypes⟦{2, 6}⟧] + (1 - sb) *
     Total[genotypes⟦{3, 7}⟧] + (1 - sb) * (1 - st) * Total[genotypes⟦{4, 8}⟧];
  msel⟦{1, 5}⟧ = Table[genotypes⟦i⟧ / u, {i, {1, 5}}];
  msel⟦{2, 6}⟧ = Table[((1 - st) * genotypes⟦i⟧) / u, {i, {2, 6}}];
  msel⟦{3, 7}⟧ = Table[((1 - sb) * genotypes⟦i⟧) / u, {i, {3, 7}}];
  msel⟦{4, 8}⟧ = Table[((1 - sb) * (1 - st) * genotypes⟦i⟧) / u, {i, {4, 8}}];

  zb = Total[msel⟦{1, 2, 5, 6}⟧] + (ab + 1) * Total[msel⟦{3, 4, 7, 8}⟧];

  z1 = Total[msel⟦{1, 5}⟧] * (c + (1 - c) * (1 / (at + 2))) +
    Total[msel⟦{2, 6}⟧] * (c + (1 - c) * ((at + 1) / (at + 2))) +
    Total[msel⟦{3, 7}⟧] * (c * (1 - γ) + (1 - c * (1 - γ)) * (1 / (at + 2))) +
    Total[msel⟦{4, 8}⟧] * (c * (1 - γ) + (1 - c * (1 - γ)) * ((at + 1) / (at + 2)));

  z2 = (Total[msel⟦{1, 5}⟧] / zb) * (c + (1 - c) * (1 / (at + 2))) +
    (Total[msel⟦{2, 6}⟧] / zb) * (c + (1 - c) * ((at + 1) / (at + 2))) +
    Total[msel⟦{3, 7}⟧] * ((1 + ab) / zb) * (c * (1 - γ) + (1 - c * (1 - γ)) * (1 / (at + 2))) +
    Total[msel⟦{4, 8}⟧] * ((1 + ab) / zb) *
```

```
   (c * (1 - γ) + (1 - c * (1 - γ)) * ((at + 1) / (at + 2)));

(*Selection on females resulting
 from cost of the female bower preference, R2*)

fsel = Table[i, {i, 1, 8}];
v = Total[genotypes[[1 ;; 4]]] + (1 - sr) * Total[genotypes[[5 ;; 8]]];
fsel[[1 ;; 4]] = Table[genotypes[[i]] / v, {i, 1, 4}];
fsel[[5 ;; 8]] = Table[((1 - sr) * genotypes[[i]]) / v, {i, 5, 8}];


(*mating probability matrix-what proportion of matings will
  be from each pairing strictly based on genotype frequency,
female choice, coercion, and bower type.*)
(*note that the mating matrix in the code here
 is the transpose of the arrangement presented in Table 2,
with female genotypes as the "rows" and male genotypes as the
 "columns." This is purely organizational; they are exactly equivalent*)

Mating = Table[Table[i, {i, 1, 8}], {j, 1, 8}];

(*for mating with R1 females*)

(*T1B1 males*)
    Mating[[1 ;; 4, {1, 5}]] =
 Table[Table[fsel[[j]] (msel[[i]] (c + (1-c)/(at+2))) / z1, {i, {1, 5}}], {j, 1, 4}];
(*T2B1 males*)
    Mating[[1 ;; 4, {2, 6}]] =
 Table[Table[fsel[[j]] (msel[[i]] (c + ((1-c) (at+1))/(at+2))) / z1, {i, {2, 6}}], {j, 1, 4}];
(*T1B2 males*)
    Mating[[1 ;; 4, {3, 7}]] =
 Table[Table[fsel[[j]] (msel[[i]] (c (1 - γ) + (1-c (1-γ))/(at+2))) / z1, {i, {3, 7}}], {j, 1, 4}];
(*T2B2 males*)
    Mating[[1 ;; 4, {4, 8}]] = Table[
  Table[fsel[[j]] (msel[[i]] (c (1 - γ) + ((1-c (1-γ)) (at+1))/(at+2))) / z1, {i, {4, 8}}], {j, 1, 4}];


(*for mating with R2 females*)

(*T1B1 males*)
```

```
    Mating[[5 ;; 8, {1, 5}]] =
   Table[Table[fsel[[j]] (msel[[i]] (c + (1-c)/(at+2)))/(zb z2), {i, {1, 5}}], {j, 5, 8}];
(*T2B1 males*)
    Mating[[5 ;; 8, {2, 6}]] =
   Table[Table[fsel[[j]] (msel[[i]] (c + ((1-c) (at+1))/(at+2)))/(zb z2), {i, {2, 6}}], {j, 5, 8}];
(*T1B2 males*)
    Mating[[5 ;; 8, {3, 7}]] = Table[
    Table[fsel[[j]] (msel[[i]] (1 + ab) (c (1 - γ) + (1-c (1-γ))/(at+2)))/(zb z2), {i, {3, 7}}], {j, 5, 8}];
(*T2B2 males*)
    Mating[[5 ;; 8, {4, 8}]] = Table[Table[
    fsel[[j]] (msel[[i]] (1 + ab) (c (1 - γ) + ((1-c (1-γ)) (at+1))/(at+2)))/(zb z2), {i, {4, 8}}], {j, 5, 8}];

(*here we sample the mating frequencies
 in order to calculate the propportion of T2 males the
 different female genotypes are mating with each generation*)

r2prop = Total[Total[Mating[[5 ;; 8, {2, 4, 6, 8}]]]] /
   (Total[Total[Mating[[5 ;; 8, {2, 4, 6, 8}]]]] +
     Total[Total[Mating[[5 ;; 8, {1, 3, 5, 7}]]]]);

r1prop = Total[Total[Mating[[1 ;; 4, {2, 4, 6, 8}]]]] /
   (Total[Total[Mating[[1 ;; 4, {2, 4, 6, 8}]]]] +
     Total[Total[Mating[[1 ;; 4, {1, 3, 5, 7}]]]]);

(*proportion of T2 matings overall, before recombination and mutation*)

t2prop = Total[Total[Mating[[1 ;; 8, {2, 4, 6, 8}]]]];

(*Multiply the mating table by the recombination
 matrix to get the contributions to each zygote genotype
 weighted by the proportions of each mate pairing*)

materec = RecTable * Mating;

(*sum across each element of each 8-tuple for the mate pairings
   across the new matrix to get the final genotype frequencies.*)
genotypes[[1 ;; 8]] =
 Table[Simplify[Total[Flatten[materec[[1 ;; 8, 1 ;; 8, i]]]]], {i, 1, 8}];
```

```
  (*once genotypes are assigned, some frequency from T2
   alleles are shifted over to T1 alleles due to biased mutation*)

  mgenotypes = {1, 2, 3, 4, 5, 6, 7, 8};
  mgenotypes[[{2, 4, 6, 8}]] = (1 - m) * genotypes[[{2, 4, 6, 8}]];
  mgenotypes[[{1, 3, 5, 7}]] = m * genotypes[[{2, 4, 6, 8}]] + genotypes[[{1, 3, 5, 7}]];

  T21 = Total[mgenotypes[[{2, 4, 6, 8}]]];
  B21 = Total[mgenotypes[[{3, 4, 7, 8}]]];
  R21 = Total[mgenotypes[[5 ;; 8]]];
  DTB1 = Total[mgenotypes[[{4, 8}]]] - T21 * B21;
  DTR1 = Total[mgenotypes[[{6, 8}]]] - T21 * R21;
  DBR1 = Total[mgenotypes[[{7, 8}]]] - B21 * R21;
  DTBR1 = mgenotypes[[8]] - T21 * DBR1 - B21 * DTR1 - R21 * DTB1 - T21 * B21 * R21;

  genotypes[[1 ;; 8]] = mgenotypes[[1 ;; 8]];
  genotypes[[9 ;; 15]] = Flatten[{ T21, B21, R21, DTB1, DTR1, DBR1, DTBR1}];

  Output = Append[Output, Flatten[{genotypes, r2prop, r1prop, t2prop}]];
  loop = loop + 1; If[loop > 1 000 000, Break[]];];

(*Uncomment following code to add the random perturbation*)

(*If[perturb==0,
    tempx1=Table[1,{i,8}];
    index=Flatten[Position[genotypes[[1;;8]],Max[genotypes[[1;;8]]]]];
    (*Find the position number of the genotype
     in the genotypes vector that is largest*)
    tempx1[[index]] = genotypes[[index]]*0.9 (*take 99% of the genotype frequency
     for the genotype of interest, this will be the new value*);
    perttot1 = (genotypes[[index]]-tempx1[[index]]);
    (*the total frequency taken from the original genotype*)
    Clear[t1];eight=Table[i,{i,8}];
    randomset=Delete[eight,index];
    Do[t1[k] = RandomReal[], {k,randomset}];
    (*creates random real numbers
     between zero and one and indexes them as t1[1], t1[2],
    t1[3].. skipping over the focal genotype that has lost frequency*)
    sumrand1 = Sum[t1[k], {k,randomset}];
    (*the sum of the random numbers
     generated associated with each other genotype*)
    Do[tempx1[[k]] = genotypes[[k]] + t1[k]*perttot1/sumrand1, {k,randomset}];
    (*divides and normalizes the random
     number and then distributes it to the other genotypes,
    indexed in the temporary vector as tempx1[[2]], tempx1[[3]], etc. basically
     each genotype receives a random portion of the frequency taken*)
    Do[genotypes[[k]] = tempx1[[k]], {k,8}];
```

```
(*last line assigns the new randomly redistributed
 genotypes back to the genotype vector used in the model loop*)
genotypes=Flatten[genotypes];

perturb=1;

T21=Total[genotypes⟦{2,4,6,8}⟧];
B21=Total[genotypes⟦{3,4,7,8}⟧];
R21=Total[genotypes⟦5;;8⟧];
DTB1=Total[genotypes⟦{4,8}⟧]-T21*B21;
DTR1=Total[genotypes⟦{6,8}⟧]-T21*R21;
DBR1=Total[genotypes⟦{7,8}⟧]-B21*R21;
DTBR1=genotypes⟦8⟧-T21*DBR1-B21*DTR1-R21*DTB1-T21*B21*R21;


genotypes⟦9;;15⟧=Flatten[{ T21,B21,R21,DTB1,DTR1,DBR1,DTBR1}];

Output=Append[Output,Flatten[{genotypes,r2prop,r1prop,t2prop}]];

Goto[start];];);*)


(*make sure to do the plots in Mathematica 10 or higher*)
```

In[ ]:=
```
(*plot of the frequencies of T, B, and R,
all together on one graph. T is black, B is blue, R is red*)

ListPlot[{Output⟦2 ;; Length[Output], 9⟧, Output⟦2 ;; Length[Output], 10⟧,
  Output⟦2 ;; Length[Output], 11⟧}, PlotRange → {0, 1.01}, Joined → True,
 PlotStyle → {{Thick, Black, Dashing[Small]}, {Thick, ColorData[1, 1]},
   {Thick, Dashing[{0.025, 0.01}], ColorData[1, 2]}},
 ImageSize → 576, DataRange → {0, Length[Output] - 2}, Frame → True,
 PlotRangePadding → {{0, 3}, {0, 0.01}}, FrameStyle →
  {{{18, Black, FontFamily → "Times"}, {18, Black, FontFamily → "Times"}},
   {{18, Black, FontFamily → "Times"}, {18, Black, FontFamily → "Times"}}},
 FrameLabel → {Text[Style["Generation", FontFamily → "Times", FontSize → 22]],
   Text[Style["Allele Frequency", FontFamily → "Times", FontSize → 22]]},
 ImagePadding → {{90, 20}, {55, 35}}, AspectRatio → 1 / (GoldenRatio)]
```

Out[ ]=

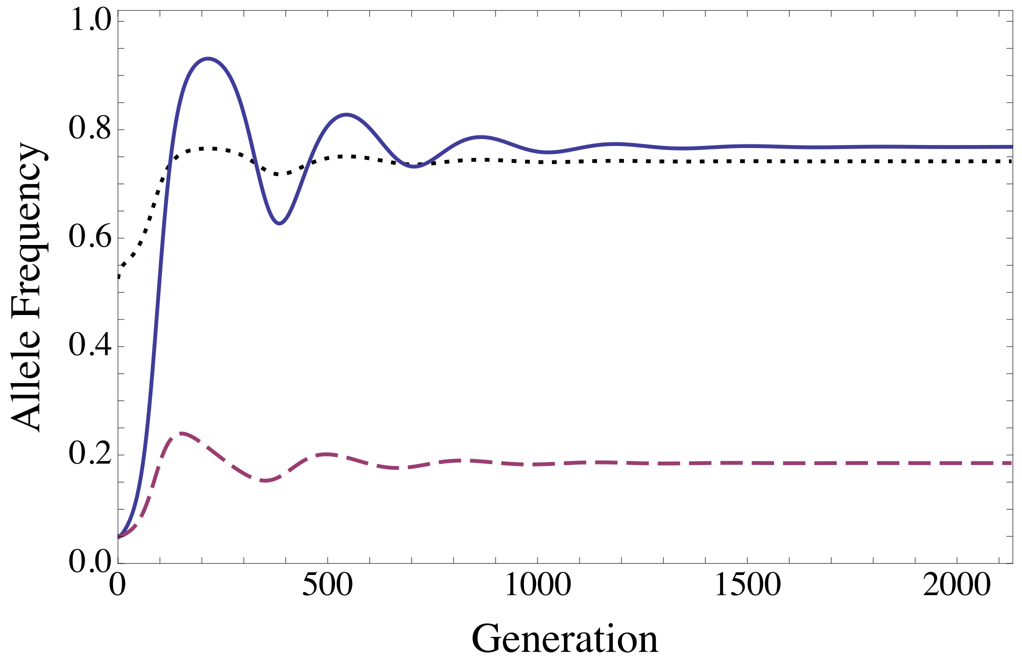

In[ ]:=
```
(*plot of the proportion of T2 males mated with by R2 and R1 females;
R1 is blue and R2 is orange,
T2 is green. The dotted line represents full autonomy*)
autonomy =
 ListPlot[{Output⟦3 ;; Length[Output], 17⟧, Output⟦3 ;; Length[Output], 16⟧,
   Output⟦3 ;; Length[Output], 18⟧, Table[t2propaut, {i, Length[Output] - 3}],
   Table[t2propeq, {i, Length[Output] - 3}]},
  PlotRange → {0.4, 1.01}, Frame → True, FrameStyle →
   {{{18, Black, FontFamily → "Times"}, {18, Black, FontFamily → "Times"}},
    {{18, Black, FontFamily → "Times"}, {18, Black, FontFamily → "Times"}}},
  FrameLabel → { Text[Style["Generation", FontFamily → "Times", FontSize → 22]],
    Pane[Text[Style[Row[{"Proportion Matings" , "    with ",
         Subscript["T", "2"], " Males"}], FontFamily → "Times"]],
     {170, All}, Alignment → Center]}, ImagePadding → {{90, 20}, {60, 10}},
  PlotRangePadding → {{0, 3}, {0, 0}}, PlotStyle → {{Thick, ColorData[97, 1]},
    {Thick, ColorData[97, 2]}, {Thick, Dashed, ColorData[97, 3]},
    {Black, Dotted, Thickness[0.002]}, {Black, Dotted, Thickness[0.002]}},
  Joined → True, DataRange → {1, Length[Output] - 2}, ImageSize → 576,
  AspectRatio → 1 / (2 * GoldenRatio),
  PlotLegends → Placed[LineLegend[{Row[{Subscript["R", "1"] , "♀"}],
      Row[{Subscript["R", "2"] , "♀"}], "overall"}, LabelStyle →
      {18, FontFamily → "Times"}, LegendLayout → "Row"], {0.66, 0.15}]]
```

Out[ ]=

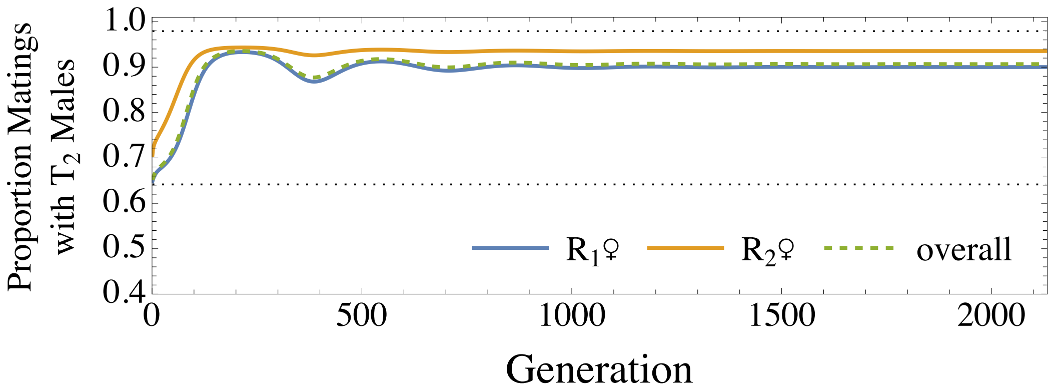


```
(*Plot of the frequecies of R2 (x axis) and B2 (y axis)
```

In[ ]:=
```
ListPlot[Table[{Output⟦i, 11⟧, Output⟦i, 10⟧}, {i, 2, Length[Output]}],
 PlotRange → Full, Joined → True, AspectRatio → 1]
```

Out[ ]=
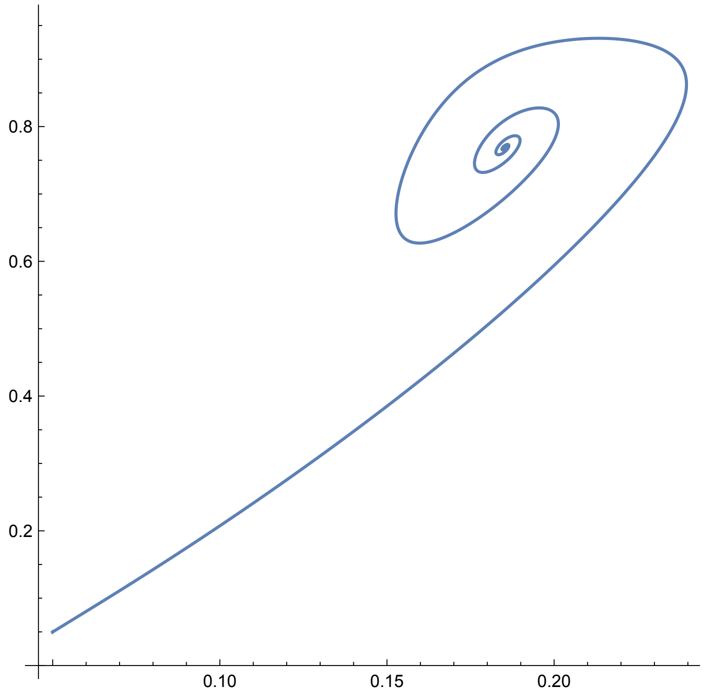

In[ ]:=
```
(*plots of all two- and three-way linkages: *)
linklabels = {"DTB", "DTR", "DBR", "DTBR"};
Table[ListPlot[Output⟦2 ;; Length[Output], i⟧,
  PlotRange → {-0.02, 0.05}, Joined → True, Filling → Axis,
  PlotLabel → linklabels⟦i - 11⟧, ImageSize → Medium], {i, 12, 15}]
```

Out[ ]= {
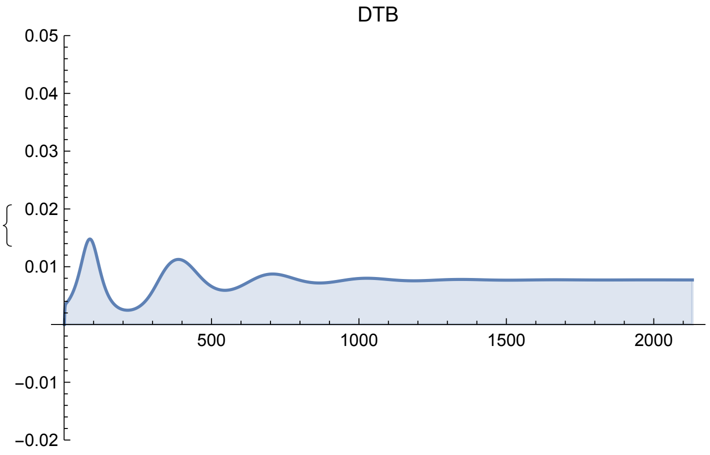

,
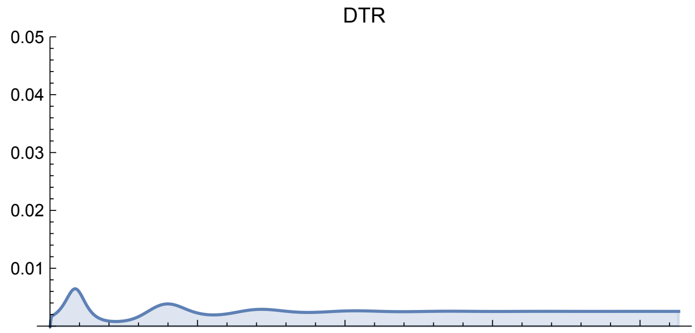

,

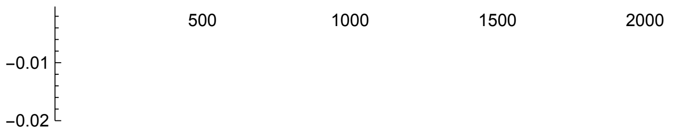
500
1000
1500
2000
−0.01
−0.02

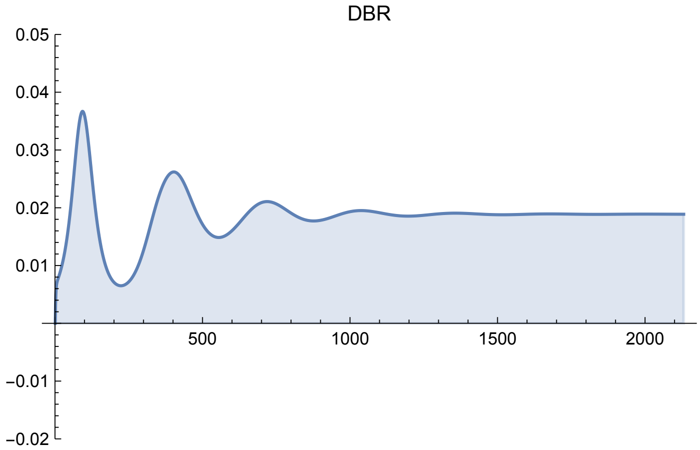
DBR
0.05
0.04
0.03
0.02
0.01
500
1000
1500
2000
−0.01
−0.02

,

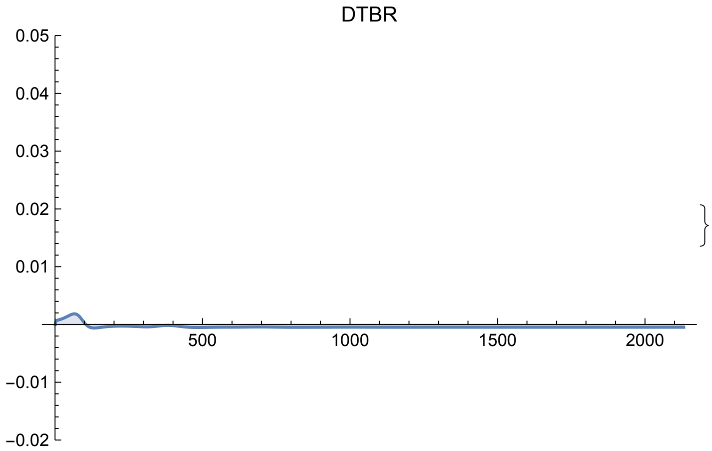
DTBR
0.05
0.04
0.03
0.02
0.01
500
1000
1500
2000
−0.01
−0.02

}

Supporting Information File S2

Mathematica code for: Remodeling and the Evolution of Sexual Autonomy by Mate Choice

Samuel S. Snow[1,2*] and Richard O. Prum[2]
*Corresponding author: samuel.s.snow@gmail.com

[1]Université Toulouse 1 Capitole and Institute for Advanced Studies in Toulouse (IAST), Toulouse, France

[2]Department of Ecology and Evolutionary Biology, Yale University, New Haven, Connecticut, 06511

We extended the classic Kirkpatrick (1982) two–locus sexual selection model to include sexually coercive male behavior (c) and mutation bias at the male display trait locus (T). We analytically solve the model for the case where the female preference allele, P2, is equal to one. This yields an expression for stable equilibria for T2 between zero and one that represents a balance of female preference, coercive behavior, viability selection, and biased mutation. We use this to calculate the starting frequency for T2 in the full model.

See Table 1 for definitions of alleles and key parameters.

*Note on parametrization: in the main text and Appendices S1–S3, we refer to the mutation parameter as "*u*" to avoid confusion with the subscript m used to denote male genotype frequencies. In the code below, the mutation parameter is denoted "m" and has been left as such to avoid the introduction of errors.

In[ ]:=

```
ClearAll[genotypes, st, at, T1P1, T1P2, T2P1, T2P2,
 postsel, Mating, u, z, init1, zygotes, r, LD, Trait, Pref,
 Thing, T2, P2, DTP, m, sp, v, b, Teq, Teq1, Teq2, c, new, recomb]
```

In[∘]:=
```
init1 = Solve[{T1P1 + T1P2 + T2P1 + T2P2 == 1, T1P2 + T2P2 == P2,
    T2P1 + T2P2 == T2, DTP == T2P2 - T2 * P2}, {T1P1, T1P2, T2P1, T2P2}];
T1P1 = T1P1 /. init1;
T1P2 = T1P2 /. init1;
T2P1 = T2P1 /. init1;
T2P2 = T2P2 /. init1;
r = 1 / 2;

new = Table[Table[i, {i, 4}], {j, 16}];
recomb = {{1, 0, 0, 0}, {1 / 2, 1 / 2, 0, 0}, {1 / 2, 0, 1 / 2, 0},
   {(1 - r) / 2, r / 2, r / 2, (1 - r) / 2}, {1 / 2, 1 / 2, 0, 0},
   {0, 1, 0, 0}, {r / 2, (1 - r) / 2, (1 - r) / 2, r / 2}, {0, 1 / 2, 0, 1 / 2},
   {1 / 2, 0, 1 / 2, 0}, {r / 2, (1 - r) / 2, (1 - r) / 2, r / 2},
   {0, 0, 1, 0}, {0, 0, 1 / 2, 1 / 2}, {(1 - r) / 2, r / 2, r / 2, (1 - r) / 2},
   {0, 1 / 2, 0, 1 / 2}, {0, 0, 1 / 2, 1 / 2}, {0, 0, 0, 1}};

genotypes = Flatten[{T1P1, T1P2, T2P1, T2P2, (T1P1 * T2P2 - T1P2 * T2P1)}];
(*the fifth element is for keeping track of LD*)

genotypes
```

Out[∘]=
```
{1 + DTP - P2 - T2 + P2 T2, -DTP + P2 - P2 T2, -DTP + T2 - P2 T2, DTP + P2 T2,
 - (-DTP + P2 - P2 T2) (-DTP + T2 - P2 T2) + (DTP + P2 T2) (1 + DTP - P2 - T2 + P2 T2)}
```

```
In[ ]:= (*make a vector that will perform Natural Selection on males,
affecting gene frequencies before mating*)

postsel = Table[i, {i, 1, 4}];
u = Total[genotypes[[1 ;; 2]]] + (1 - st) * Total[genotypes[[3 ;; 4]]];
postsel[[1 ;; 2]] = Table[genotypes[[i]] / u, {i, 1, 2}];
postsel[[3 ;; 4]] = Table[((1 - st) * genotypes[[i]]) / u, {i, 3, 4}];

z = Total[postsel[[1 ;; 2]]] * (c + (1 - c) * (1 / (at + 2))) +
   Total[postsel[[3 ;; 4]]] * (c + (1 - c) * ((at + 1) / (at + 2)));

Mating = Table[Table[i, {i, 1, 4}], {j, 1, 4}];

Table[
  Mating[[j, 1 ;; 4]] = Table[(genotypes[[j]] * postsel[[i]]), {i, 1, 4}], {j, {1, 3}}];

Table[Mating[[j, 1 ;; 2]] =
   Table[(genotypes[[j]] * postsel[[i]] * (c + (1 - c) * (1 / (at + 2)))) / z, {i, 1, 2}], {j,
   {2, 4}}];

Table[Mating[[j, 3 ;; 4]] =
   Table[(genotypes[[j]] * postsel[[i]] * (c + (1 - c) * ((at + 1) / (at + 2)))) / z,
    {i, 3, 4}], {j, {2, 4}}];

s = 0;
Do[
  Do[
   s = s + 1;
   new[[s, 1 ;;]] = Mating[[h, k]] * recomb[[s, 1 ;;]], {k, 4}], {h, 4}];

genotypes[[1 ;; 4]] = Table[Total[new[[1 ;;, i]]], {i, 4}];

(*once genotype frequencies are assigned, adjust due to biased mutation*)

mgenotypes = {1, 2, 3, 4, 5};
mgenotypes[[3 ;; 4]] = (1 - m) * genotypes[[3 ;; 4]];
mgenotypes[[1 ;; 2]] = m * genotypes[[3 ;; 4]] + genotypes[[1 ;; 2]];
mgenotypes[[5]] = mgenotypes[[1]] * mgenotypes[[4]] - mgenotypes[[2]] * mgenotypes[[3]];

genotypes = mgenotypes;

In[ ]:= (*get the recursion equations*)
ΔT2 = Simplify[(genotypes[[3]] + genotypes[[4]]) - T2];
ΔP2 = Simplify[(genotypes[[2]] + genotypes[[4]]) - P2];
ΔLD = Simplify[genotypes[[5]] - DTP];
```

In[]:=
```
(*look at the expression for ΔT2 when preference is fixed (P2=1)*)
ΔT2 = FullSimplify[ΔT2 /. {P2 → 1, DTP → 0}]
```

Out[]=
$$(T2\,(at\,(-(m+1)\,T2\,(c+st-1)+c\,m+c-m\,st+m+st-1)-(c+1)\,(m\,(st\,T2+st-2)+st\,(T2-1)))))\,/\,(2\,T2\,(at\,(c+st-1)+c\,st+st)-2\,(at\,c+c+1))$$

In[]:=
```
ΔT2noc = FullSimplify[ΔT2 /. {P2 → 1, DTP → 0, c → 0}]
```

Out[]=
$$\frac{T2\,(2\,m+st-at\,(-1+st)\,(-1+m+T2+m\,T2)-st\,(m+T2+m\,T2))}{-2+2\,(at\,(-1+st)+st)\,T2}$$

```
(*Confirm that it is the same as Kirkpatrick 1982 with mutation bias when c=0*)
```

In[]:=
```
Simplify[-((T2 (st (-1 + T2) + a (-1 + st) (-1 + m + T2 + m T2) + m (-2 + st + st T2))) /
    (2 (-1 + a (-1 + st) T2 + st T2))) == ΔT2noc /. at → a]
```

Out[]= True

```
(*Solve for Equilibria when P2 is fixed at 1*)
```

In[]:=
```
Simplify[ΔP2 /. {P2 → 1, DTP → 0}]
```

Out[]= 0

In[]:=
```
Simplify[ΔLD /. {P2 → 1, DTP → 0}]
```

Out[]= 0

In[]:=
```
Simplify[Solve[ΔT2 == 0, T2]]
```

Out[]=
$$\left\{\{T2\to 0\},\left\{T2\to \frac{-(1+c)\,(m\,(-2+st)-st)+at\,(-1+c+m+c\,m+st-m\,st)}{(1+m)\,((1+c)\,st+at\,(-1+c+st))}\right\}\right\}$$

In[]:=
```
Teq1 = FullSimplify[(-(1 + c) (m (-2 + st) - st) + at (-1 + c + m + c m + st - m st)) / ((1 + m) ((1 + c) st + at (-1 + c + st)))]
```

Out[]=
$$\frac{1-m+\frac{2\,(1+c+at\,c)\,m}{st+c\,st+at\,(-1+c+st)}}{1+m}$$

Test the stability of the equilibria by looking at the sign of the second derivative at the equilibrium points, given the constraints on the magnitudes of st, m, and c

In[]:=
```
sec1 = D[ΔT2, T2] /. T2 → Teq1;
sec0 = D[ΔT2, T2] /. T2 → 0;
```

In[]:=
```
Reduce[sec1 < 0 && 0 < m < 1 && 0 < at && 0 < st < 1 && 0 < c < 1, {at, c}]
```

Out[]=
$$0<st<1\,\&\&\,0<m<1\,\&\&\,at>\frac{2\,m+st-m\,st}{1-m-st+m\,st}\,\&\&\,0<c<\frac{at-2\,m-at\,m-st-at\,st+m\,st+at\,m\,st}{at+2\,m+at\,m+st-m\,st}$$

In[•]:=
```
Reduce[sec0 < 0 && 0 < m < 1 && 0 < at && 0 < st < 1 && 0 < c < 1, {at, c}]
```

Out[•]=
```
0 < st < 1 && 0 < m < 1 && ((0 < at ≤ (2 m + st - m st)/(1 - m - st + m st) && 0 < c < 1) ||
  (at > (2 m + st - m st)/(1 - m - st + m st) && (at - 2 m - at m - st - at st + m st + at m st)/(at + 2 m + at m + st - m st) < c < 1))
```

In[•]:=
```
FullSimplify[(2 m + st - m st)/(1 - m - st + m st)]
```

Out[•]=
```
-1 + (1 + m)/((-1 + m) (-1 + st))
```

In[•]:=
```
FullSimplify[(at - 2 m - at m - st - at st + m st + at m st)/(at + 2 m + at m + st - m st)]
```

Out[•]=
```
(at - 2 m - at m + (1 + at) (-1 + m) st)/(at + 2 m + at m + st - m st)
```

```
(*The T2=Teq equilibrium is stable as long as at >
  -1+(1+m)/((-1+m) (-1+st)) and 0 < c <(at-2 m-at m+(1+at) (-1+m) st)/(at+2 m+at m+st-m st).*)
```

In[•]:=
```
Manipulate[{-1 + (1 + m)/((-1 + m) (-1 + st)), (at - 2 m - at m + (1 + at) (-1 + m) st)/(at + 2 m + at m + st - m st)},
  {{m, 0.1}, 0, 1}, {{st, 0.1}, 0, 1}, {{at, 4}, -1 + (1 + m)/((-1 + m) (-1 + st)), 30}]
```

Out[•]=

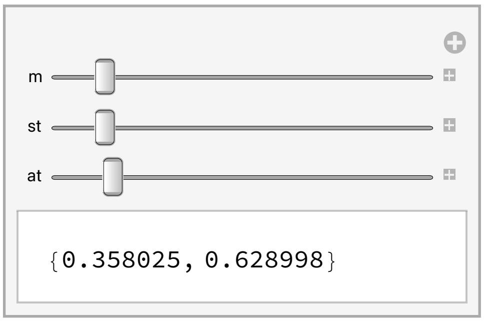

Demonstrate Stability Graphically

In[156]:=
```
fΔT2[T2_, at_, st_, c_, m_] :=
  (T2 (at (-1 + c + m + c m + st - m st - (1 + m) (-1 + c + st) T2) -
       (1 + c) (st (-1 + T2) + m (-2 + st + st T2)))) /
   (-2 (1 + c + at c) + 2 (st + c st + at (-1 + c + st)) T2);
```

In[157]:= Manipulate[{Plot[fΔT2[T2, at, st, c, m], {T2, 0, 1}, PlotRange → Full],

$$-1+\frac{1+m}{(-1+m)\ (-1+st)},\ \frac{at-2\,m-at\,m+(1+at)\ (-1+m)\ st}{at+2\,m+at\,m+st-m\,st}\Big\},$$

{{at, 8}, 0, 20}, {{c, 0.5}, 0, 1}, {{st, 0.1}, 0, 1}, {{m, 0.1}, 0, 1}]

Out[157]=

at

c

st

m

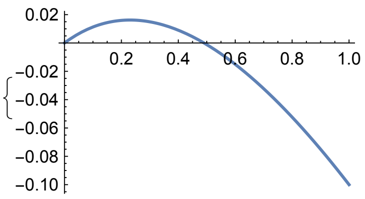

{ , 0.358025, 0.680968}

Use the equilibrium condition to calculate the initial adjusted realized strength of female preference:

(*First, find the value of Teq when c=0*)

In[∘]:= $$\frac{1-m+\frac{2\,(1+c+at\,c)\,m}{st+c\,st+at\,(-1+c+st)}}{1+m}\ /.\ c\to 0$$

Out[∘]= $$\frac{1-m+\frac{2\,m}{at\,(-1+st)+st}}{1+m}$$

(*Then we can ask what value of at in the case of c=
0 would result in the same equilibrium value for T2 with coercion in
the system. This is the "operational" or "realized" magnitude of at*)

In[∘]:= $$\text{Solve}\left[\frac{1-m+\frac{2\,(1+c+at\,c)\,m}{st+c\,st+at\,(-1+c+st)}}{1+m}==\frac{1-m+\frac{2\,m}{atreal\,(-1+st)+st}}{1+m},\ atreal\right]$$

Out[∘]= $$\left\{\left\{atreal\to\frac{at-at\,c}{1+c+at\,c}\right\}\right\}$$

In[∘]:= $$\text{Factor}\left[\frac{at-at\,c}{1+c+at\,c}\right]$$

Out[∘]= $$-\frac{at\,(c-1)}{at\,c+c+1}$$

```
(*We can plot the relationship between c and atreal:
  atreal is always less than at. We can also see that the minimum value for
    atreal coincides with the maximum value for c for a stable initial non-
   zero T2 equilibrium (the x axis range limit) as well as
    with the minimum value for at (the orange line)*)
```

In[•]:=
$$\text{Plot}\left[\left\{-\frac{\text{at}\,(c-1)}{\text{at}\,c+c+1}\;/.\;\text{at}\to 12,\;-1+\frac{1+m}{(-1+m)\,(-1+\text{st})}\;/.\;\{m\to 0.1,\ \text{st}\to 0.1\}\right\},\;\left\{c,\,0,\,\frac{\text{at}-2m-\text{at}\,m+(1+\text{at})\,(-1+m)\,\text{st}}{\text{at}+2m+\text{at}\,m+\text{st}-m\,\text{st}}\;/.\;\{\text{at}\to 12,\ m\to 0.1,\ \text{st}\to 0.1\}\right\},\;\text{PlotRange}\to\{0,\,12.5\}\right]$$

Out[•]=
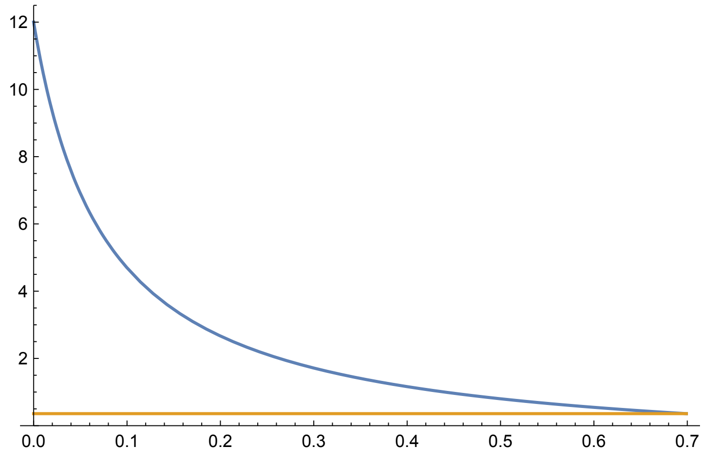

Further, for the equilibrium condition, calculate the proportion of T2 males that females are able to mate with. Then evaluate at c = 0 to get the proportion o f T 2 males they would get if they had full sexual autonomy

In[•]:=
```
ClearAll[a, c, ab, m, st, Teq, Taut, initr2prop,
  T2, P2, DTP, ToPo, ToP, TPo, TP, T1P1, T1P2, T2P1, T2P2];
```

In[•]:=
```
T2 = Teq;
P2 = 1;
DTP = 0;
```

In[•]:=
```
init1 = Solve[{T1P1 + T1P2 + T2P1 + T2P2 == 1, T1P2 + T2P2 == P2,
    T2P1 + T2P2 == T2, DTP == T2P2 - T2 * P2}, {T1P1, T1P2, T2P1, T2P2}];
```

In[•]:=
```
T1P1 = T1P1 /. init1;
T1P2 = T1P2 /. init1;
T2P1 = T2P1 /. init1;
T2P2 = T2P2 /. init1;
```

In[•]:=
```
genotypes = Flatten[{T1P1, T1P2, T2P1, T2P2, (T1P1 * T2P2 - T1P2 * T2P1)}];
```

In[•]:=
```
genotypes
```

Out[•]=
```
{0, 1 - Teq, 0, Teq, 0}
```

In[ ]:=
```
postsel = Table[i, {i, 1, 4}];
u = Total[genotypes⟦1 ;; 2⟧] + (1 - st) * Total[genotypes⟦3 ;; 4⟧];
postsel⟦1 ;; 2⟧ = Table[genotypes⟦i⟧ / u, {i, 1, 2}];
postsel⟦3 ;; 4⟧ = Table[((1 - st) * genotypes⟦i⟧) / u, {i, 3, 4}];
```

In[ ]:=
```
postsel
```

Out[ ]=

$$\left\{0, \frac{1-\text{Teq}}{1-\text{Teq}+(1-\text{st})\,\text{Teq}}, 0, \frac{(1-\text{st})\,\text{Teq}}{1-\text{Teq}+(1-\text{st})\,\text{Teq}}\right\}$$

In[ ]:=
```
z = Total[postsel⟦1 ;; 2⟧] * (c + (1 - c) * (1 / (at + 2))) +
   Total[postsel⟦3 ;; 4⟧] * (c + (1 - c) * ((at + 1) / (at + 2)));

Mating = Table[Table[i, {i, 1, 4}], {j, 1, 4}];

Table[
  Mating⟦j, 1 ;; 4⟧ = Table[(genotypes⟦j⟧ * postsel⟦i⟧), {i, 1, 4}], {j, {1, 3}}];

Table[Mating⟦j, 1 ;; 2⟧ =
   Table[(genotypes⟦j⟧ * postsel⟦i⟧ * (c + (1 - c) * (1 / (at + 2)))) / z, {i, 1, 2}], {j,
   {2, 4}}];

Table[Mating⟦j, 3 ;; 4⟧ =
   Table[(genotypes⟦j⟧ * postsel⟦i⟧ * (c + (1 - c) * ((at + 1) / (at + 2)))) / z,
    {i, 3, 4}], {j, {2, 4}}];

(* male genotypes are the columns and female genotypes are the rows,
in the order T1P1, T1P2, T2P1, T2P2*)
MatrixForm[Mating]
```

Out[ ]//MatrixForm=

$$\begin{pmatrix} 0 & 0 & 0 & 0 \\ 0 & \frac{\left(\frac{1-c}{2+at}+c\right)(1-\text{Teq})^2}{(1-\text{Teq}+(1-\text{st})\,\text{Teq})\left(\frac{\left(\frac{1-c}{2+at}+c\right)(1-\text{Teq})}{1-\text{Teq}+(1-\text{st})\,\text{Teq}}+\frac{\left(\frac{(1+at)(1-c)}{2+at}+c\right)(1-\text{st})\,\text{Teq}}{1-\text{Teq}+(1-\text{st})\,\text{Teq}}\right)} & 0 & \frac{\left(\frac{(1+at)(1-c)}{2+at}+c\right)(1-\text{st})(1-\text{Teq})\,\text{Teq}}{(1-\text{Teq}+(1-\text{st})\,\text{Teq})\left(\frac{\left(\frac{1-c}{2+at}+c\right)(1-\text{Teq})}{1-\text{Teq}+(1-\text{st})\,\text{Teq}}+\frac{\left(\frac{(1+at)(1-c)}{2+at}+c\right)(1-\text{st})\,\text{Teq}}{1-\text{Teq}+(1-\text{st})\,\text{Teq}}\right)} \\ 0 & 0 & 0 & 0 \\ 0 & \frac{\left(\frac{1-c}{2+at}+c\right)(1-\text{Teq})\,\text{Teq}}{(1-\text{Teq}+(1-\text{st})\,\text{Teq})\left(\frac{\left(\frac{1-c}{2+at}+c\right)(1-\text{Teq})}{1-\text{Teq}+(1-\text{st})\,\text{Teq}}+\frac{\left(\frac{(1+at)(1-c)}{2+at}+c\right)(1-\text{st})\,\text{Teq}}{1-\text{Teq}+(1-\text{st})\,\text{Teq}}\right)} & 0 & \frac{\left(\frac{(1+at)(1-c)}{2+at}+c\right)(1-\text{st})\,\text{Teq}^2}{(1-\text{Teq}+(1-\text{st})\,\text{Teq})\left(\frac{\left(\frac{1-c}{2+at}+c\right)(1-\text{Teq})}{1-\text{Teq}+(1-\text{st})\,\text{Teq}}+\frac{\left(\frac{(1+at)(1-c)}{2+at}+c\right)(1-\text{st})\,\text{Teq}}{1-\text{Teq}+(1-\text{st})\,\text{Teq}}\right)} \end{pmatrix}$$

In[ ]:=
```
initr2prop = FullSimplify[(Mating⟦2, 4⟧ + Mating⟦4, 4⟧)]
```

Out[ ]=

$$\frac{(1+at+c)\,(-1+st)\,\text{Teq}}{-1-(1+at)\,c+(st+c\,st+at\,(-1+c+st))\,\text{Teq}}$$

In[ ]:=
```
(*proportion matings with T2 males with coercion*)
t2propeq = FullSimplify[initr2prop /. Teq → Teq1]
```

Out[ ]=

$$1-\frac{2\,(1+c+at\,c)\,m}{(-1+m)\,(st+c\,st+at\,(-1+c+st))}$$

```
(*calculate the proportion of T2 males a female gets when c=
 0. This will be equal to the proportion
   of T2 males females gets when γ=1 and B2=1*)
```

In[ ]:= `t2propaut = FullSimplify[t2propeq /. c → 0]`

Out[ ]= $1 - \frac{2\,m}{(-1 + m)\,(at\,(-1 + st) + st)}$

Supporting Information File S3

Mathematica code for: Remodeling and the Evolution of Sexual Autonomy by Mate Choice

Samuel S. Snow[1,2*] and Richard O. Prum[2]
*Corresponding author: samuel.s.snow@gmail.com

[1]Université Toulouse 1 Capitole and Institute for Advanced Studies in Toulouse (IAST), Toulouse, France

[2]Department of Ecology and Evolutionary Biology, Yale University, New Haven, Connecticut, 06511

Two–preference version of the three–locus population–genetic Remodeling model. In this version, we explore a case where R2 females prefer B2 males, but rather than mate randomly, R1 females have an equally strong preference for B1 males. We start the population at r2=0.5 and b2=0.5 so we can examine the effect of the protectiveness of the B2 trait, γ, in favoring one kind of female preference over another. This code explores the "invasion" of the R2 allele via analysis of the model recursion equations.

See Table 1 for definitions of alleles and key parameters.

*Note on parametrization: in the main text and Appendices S1–S3, we refer to the mutation parameter as "u" to avoid confusion with the subscript m used to denote male genotype frequencies. In the code below, the mutation parameter is denoted "m" and has been left as such to avoid the introduction of errors.

```
ClearAll[st, sb, at, c, γ, m, init, T1B1R1, T2B1R1, T1B2R1, T2B2R1, T1B1R2, T2B1R2,
  T1B2R2, T2B2R2, genotypes, msel, fsel, r, Output, u, ab, z1, z2, zb, T2, B2,
  R2, DTB, DTR, DBR, DTBR, altered, Mating, s, mgenotypes, T21, B21, R21, DTB1,
```

```
  DTR1, DBR1, DTBR1, ΔT2, ΔB2, ΔR2, ΔDTB, ΔDTR, ΔDBR, ΔDTBR, perturb, sr, zb0,
  materec, RecTable, MaskProb, ZygoteGenotypes, prob, zyg1, zyg2, zygoteProb];

(*three-locus popgen simulation of
  Remodeling without direct costs to coercive mating*)

(*this version uses a probabilistic formulation for
 how females visit and mate at male territories. Females may
 either visit territories randomly or choose territories based
 on the kind of remodeling trait (bower) they see. Once there,
the male may either coerce them into mating there without the
 opportunity to evaluate their display, or the female may choose
 the mate with that male (given that he has not already coerced her)
 by some factor related to his attractivenes.*)

(*There is a locus for male display trait T2/T1,
    remodeling trait (protective bower) B2/B1,
    and female preference for the type of Remodeling trait (bower),
    R2/R1. We make two underlying assumptions in this model: 1)
   that all males will coerce if they have the opportunity, and 2)
 that all females have a preference for attractive T2 males*)

(*parameters:
 (1-st) is the fraction by which males having the T2 allele survive relative
  to Males having the T1 allele. (1-sb) is the fraction by which males
  having the B2 allele survive relative to Males having the B1 allele.
   (1-sr) is the fraction by which females having the
  R2 allele survive relative to females having the R1 allele.
   (at+1) is the factor by which attractive T2 allele males
  have a relative advantage in mating success over T1 males.

   **In this version of the model, (ab+1) is the factor by
 which females with the R2 allele prefer to visit B2 bowers,
AND ALSO the factor by which R1 females prefer B1 bowers,
so there is an equally strong preference for
 either. The only difference will be γ affecting the B2 males.

  c is a number between 0 and 1 representing the
 rate of successful forced fertilization of a female,
(or the likelihood of coercive attack) by a
  male without a protective bower (B2).
   γ is a number between 0 and 1 representing the effectiveness of
  protective bowers at reducing a male's ability to coerce. If γ=0,
then there is no effect, and if γ=1, then B2 males are unable to coerce,
and females are free to choose the male based
 on his attractiveness once they are at his territory.
```

Lastly, we make an assumption of biased mutation at the T locus. This is meant as a proxy for a quantitative, multialleleic trait or some other process by which variation is maintained at the T locus, so that sexual selection is ongoing and we can explore the effects of sexual conflict over indirect effects in a simple population-genetic framework*)

(*the equilibrium conditions for a non-zero T2 in the absence of B and R are: 0<st<1&&0<m<1&&at>-1+$\frac{1+m}{(-1+m)\ (-1+st)}$&&0<c<$\frac{at-2\ m-at\ m-st-at\ st+m\ st+at\ m\ st}{at+2\ m+at\ m+st-m\ st}$. See Appendix S2 and Supporting Information File S2 for derivation*)

(*use these conditions to determine appropriate parameter values*)

$$\text{Manipulate}\left[\left\{-1+\frac{1+m}{(-1+m)\ (-1+st)},\ \frac{at-2\,m-at\,m+(1+at)\ (-1+m)\ st}{at+2\,m+at\,m+st-m\,st}\right\},\right.$$
$$\left.\{\{m,0.1\},0,1\},\{\{st,0.1\},0,1\},\left\{\{at,4\},-1+\frac{1+m}{(-1+m)\ (-1+st)},30\right\}\right];$$

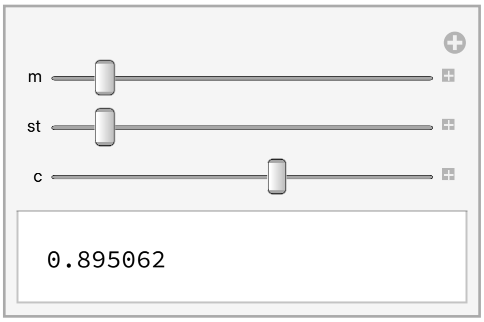

;

(*set parameters*)

```
st = 1 / 10;
sb = 0;
sr = 0;

(*recombination and mutation rate*)
rtb = 1 / 2;
rbr = 1 / 2;
m = 1 / 10;
```

(*The initial frequency for T2 is the equilibrium value for when B2 and R2 equal zero. This is where female preference for T2, mutation bias, and male coercion are in balance;
See Appendix S2 and Supporting Information File S2 for derivation.*)

$$Teq = \frac{1 - m + \frac{2\ (1+c+at\ c)\ m}{st+c\ st+at\ (-1+c+st)}}{1 + m};$$

```
init = Solve[{T1B1R1 + T2B1R1 + T1B2R1 + T2B2R1 + T1B1R2 + T2B1R2 + T1B2R2 + T2B2R2 == 1,
     T2 == T2B1R1 + T2B2R1 + T2B1R2 + T2B2R2, B2 == T1B2R1 + T2B2R1 + T1B2R2 + T2B2R2,
     R2 == T1B1R2 + T2B1R2 + T1B2R2 + T2B2R2, DTB == (T2B2R1 + T2B2R2) - T2 * B2,
     DTR == (T2B1R2 + T2B2R2) - T2 * R2, DBR == (T1B2R2 + T2B2R2) - B2 * R2,
     DTBR == T2B2R2 - T2 * ((T1B2R2 + T2B2R2) - B2 * R2) -
       B2 * ((T2B1R2 + T2B2R2) - T2 * R2) - R2 * ((T2B2R1 + T2B2R2) - T2 * B2) - T2 * B2 * R2},
    {T1B1R1, T2B1R1, T1B2R1, T2B2R1, T1B1R2, T2B1R2, T1B2R2, T2B2R2}];

T1B1R1 = T1B1R1 /. init;
T2B1R1 = T2B1R1 /. init;
T1B2R1 = T1B2R1 /. init;
T2B2R1 = T2B2R1 /. init;
T1B1R2 = T1B1R2 /. init;
T2B1R2 = T2B1R2  /. init;
T1B2R2 = T1B2R2 /. init;
T2B2R2 = T2B2R2 /. init;


(*genotypes vector with initial allele frequencies and linkages*)
genotypes = Flatten[{T1B1R1, T2B1R1, T1B2R1, T2B2R1,
     T1B1R2, T2B1R2, T1B2R2, T2B2R2, T2, B2, R2, DTB, DTR, DBR, DTBR}];

(*set up the table of recombination probabilities that will yield the
 distribution of zygote genotypes resulting from each mated pairing*)

Nloci = 3;
Geno = 2^Nloci;
masklim = 2^(Nloci - 1);
recrate[1] = rtb;
recrate[2] = rbr;

(*Maskprob gives the probability for a particular pattern of
 recombination (a mask),based on given values of recombination
 rates between different loci ("recrate" vector above)*)

MaskProb = Function[{mask, Nloci},
   For[prob = 1; z = 1, z < Nloci, z++,
    If[BitGet[mask, z] == BitGet[mask, z - 1],
     prob *= (1 - recrate[z]),
     prob *= recrate[z]]]];

(*generates the two zygote genotypes (zyg1 and zyg2)
```

```
 from two parental genotypes (i and j) for a given mask.*)

ZygoteGenotypes = Function[{i, j, mask},
   For[zyg1 = 0; zyg2 = 0; k = 0, k < Nloci, k++,
    If[BitGet[mask, k] == 0,
     zyg1 = BitOr[zyg1, (BitAnd[i, (2^k)])];
     zyg2 = BitOr[zyg2, (BitAnd[j, (2^k)])],
     zyg1 = BitOr[zyg1, (BitAnd[j, (2^k)])];
     zyg2 = BitOr[zyg2, (BitAnd[i, (2^k)])]]]];

(*Produce a 3-dimentional matrix with the cells representing
  the probability of producing a zygote genotype (k) from parental
  genotypes i (mother) and j (father). Multiplying this matrix with
  the mating table that gives the proportions of different male-
 female genotype mating combinations will give the
  frequency of zygote genotypes in the new generation
  (after summing across parental genotypes).*)

RecTable = Table[0, {i, Geno}, {j, Geno}, {k, Geno}];
For[i = 0, i < Geno, i++,
 For[j = 0, j < Geno, j++,
  For[mask = 0, mask < masklim, mask++,
   MaskProb[mask, Nloci];
   zygoteProb = (1 / 2) * prob;
   ZygoteGenotypes[i, j, mask];
   RecTable[[(i + 1), (j + 1), (zyg1 + 1)]] += zygoteProb;
   RecTable[[(i + 1), (j + 1), (zyg2 + 1)]] += zygoteProb;]]]

(*uncomment this line to view the recombination table*)
(*MatrixForm[RecTable]*)


(*make a vector that will perform Natural Selection on males,
affecting gene frequencies before mating.*)

msel = Table[i, {i, 1, 8}];
u = Total[genotypes[[{1, 5}]]] + (1 - st) * Total[genotypes[[{2, 6}]]] +
   (1 - sb) * Total[genotypes[[{3, 7}]]] + (1 - sb) * (1 - st) * Total[genotypes[[{4, 8}]]];
msel[[{1, 5}]] = Table[genotypes[[i]] / u, {i, {1, 5}}];
msel[[{2, 6}]] = Table[((1 - st) * genotypes[[i]]) / u, {i, {2, 6}}];
msel[[{3, 7}]] = Table[((1 - sb) * genotypes[[i]]) / u, {i, {3, 7}}];
msel[[{4, 8}]] = Table[((1 - sb) * (1 - st) * genotypes[[i]]) / u, {i, {4, 8}}];

zb1 = (ab + 1) * Total[msel[[{1, 2, 5, 6}]]] + Total[msel[[{3, 4, 7, 8}]]];

zb2 = Total[msel[[{1, 2, 5, 6}]]] + (ab + 1) * Total[msel[[{3, 4, 7, 8}]]];
```

```
z1 = Total[msel⟦{1, 5}⟧] * ((1 + ab) / zb1) * (c + (1 - c) * (1 / (at + 2))) +
   Total[msel⟦{2, 6}⟧] * ((1 + ab) / zb1) * (c + (1 - c) * ((at + 1) / (at + 2))) +
   (Total[msel⟦{3, 7}⟧] / zb1) * (c * (1 - γ) + (1 - c * (1 - γ)) * (1 / (at + 2))) +
   (Total[msel⟦{4, 8}⟧] / zb1) * (c * (1 - γ) + (1 - c * (1 - γ)) * ((at + 1) / (at + 2)));

z2 = (Total[msel⟦{1, 5}⟧] / zb2) * (c + (1 - c) * (1 / (at + 2))) +
   (Total[msel⟦{2, 6}⟧] / zb2) * (c + (1 - c) * ((at + 1) / (at + 2))) +
   Total[msel⟦{3, 7}⟧] * ((1 + ab) / zb2) * (c * (1 - γ) + (1 - c * (1 - γ)) * (1 / (at + 2))) +
   Total[msel⟦{4, 8}⟧] * ((1 + ab) / zb2) *
    (c * (1 - γ) + (1 - c * (1 - γ)) * ((at + 1) / (at + 2)));
(*add a small cost to the bower preference, R2*)

fsel = Table[i, {i, 1, 8}];
v = Total[genotypes⟦1 ;; 4⟧] + (1 - sr) * Total[genotypes⟦5 ;; 8⟧];
fsel⟦1 ;; 4⟧ = Table[genotypes⟦i⟧ / v, {i, 1, 4}];
fsel⟦5 ;; 8⟧ = Table[((1 - sr) * genotypes⟦i⟧) / v, {i, 5, 8}];


(*mating porbability matrix-what proportion of matings
  will be from each pairing strictly based on genotype frequency,
female choice, coercion, and bower type.*)

Mating = Table[Table[i, {i, 1, 8}], {j, 1, 8}];

(*for mating with R1 females*)

(*T1B1 males*)
    Mating⟦1 ;; 4, {1, 5}⟧ =
  Table[Table[fsel⟦j⟧ (msel⟦i⟧ (1 + ab) (c + (1-c)/(at+2))) / (zb1 z1), {i, {1, 5}}], {j, 1, 4}];
(*T2B1 males*)
    Mating⟦1 ;; 4, {2, 6}⟧ =
  Table[Table[fsel⟦j⟧ (msel⟦i⟧ (1 + ab) (c + (1-c) (at+1)/(at+2))) / (zb1 z1), {i, {2, 6}}], {j, 1, 4}];
(*T1B2 males*)
    Mating⟦1 ;; 4, {3, 7}⟧ =
  Table[Table[fsel⟦j⟧ (msel⟦i⟧ (c (1 - γ) + (1-c (1-γ))/(at+2))) / (zb1 z1), {i, {3, 7}}], {j, 1, 4}];
(*T2B2 males*)
    Mating⟦1 ;; 4, {4, 8}⟧ = Table[
   Table[fsel⟦j⟧ (msel⟦i⟧ (c (1 - γ) + (1-c (1-γ)) (at+1)/(at+2))) / (zb1 z1), {i, {4, 8}}], {j, 1, 4}];
```

```
(*for mating with R2 females*)

(*T1B1 males*)
    Mating[[5 ;; 8, {1, 5}]] =
  Table[Table[fsel[[j]] (msel[[i]] (c + (1-c)/(at+2)))/(zb2 z2), {i, {1, 5}}], {j, 5, 8}];
(*T2B1 males*)
    Mating[[5 ;; 8, {2, 6}]] =
  Table[Table[fsel[[j]] (msel[[i]] (c + ((1-c) (at+1))/(at+2)))/(zb2 z2), {i, {2, 6}}], {j, 5, 8}];
(*T1B2 males*)
    Mating[[5 ;; 8, {3, 7}]] = Table[
   Table[fsel[[j]] (msel[[i]] (1 + ab) (c (1 - γ) + (1-c (1-γ))/(at+2)))/(zb2 z2), {i, {3, 7}}], {j, 5, 8}];
(*T2B2 males*)
    Mating[[5 ;; 8, {4, 8}]] = Table[Table[
    fsel[[j]] (msel[[i]] (1 + ab) (c (1 - γ) + ((1-c (1-γ)) (at+1))/(at+2)))/(zb2 z2), {i, {4, 8}}], {j, 5, 8}];

(*Multiply the mating table by the recombination
 matrix to get the contributions to each zygote genotype
 weighted by the proportions of each mate pairing*)

materec = RecTable * Mating;

(*sum across each element of each 8-tuple for the mate pairings
  across the new matrix to get the final genotype frequencies.*)
genotypes[[1 ;; 8]] =
  Table[Simplify[Total[Flatten[materec[[1 ;; 8, 1 ;; 8, i]]]]], {i, 1, 8}];



(*once genotypes are assigned, some frequency from T2
 alleles are shifted over to T1 alleles due to biased mutation*)

mgenotypes = {1, 2, 3, 4, 5, 6, 7, 8};
mgenotypes[[{2, 4, 6, 8}]] = (1 - m) * genotypes[[{2, 4, 6, 8}]];
mgenotypes[[{1, 3, 5, 7}]] = m * genotypes[[{2, 4, 6, 8}]] + genotypes[[{1, 3, 5, 7}]];

T21 = Total[mgenotypes[[{2, 4, 6, 8}]]];
B21 = Total[mgenotypes[[{3, 4, 7, 8}]]];
R21 = Total[mgenotypes[[5 ;; 8]]];
DTB1 = Total[mgenotypes[[{4, 8}]]] - T21 * B21;
DTR1 = Total[mgenotypes[[{6, 8}]]] - T21 * R21;
```

```
DBR1 = Total[mgenotypes⟦{7, 8}⟧] - B21 * R21;
DTBR1 = mgenotypes⟦8⟧ - T21 * DBR1 - B21 * DTR1 - R21 * DTB1 - T21 * B21 * R21;


(*We are interested in evolution by indirect selection,
so we have to look ahead to the second
 generation to see if R2 and B2 will increase*)

(*for the first generation, the D's start at zero,
so the new value will just be the magnitude of the ΔD.*)

(*first, get the recursion equations and solve for the first generation
  Beware- this code cell is computationally intensive and may
  take a very long time to run (about an hour on an intel i7 iMac)*)

ΔT2 = Simplify[T21 - T2];
ΔB2 = Simplify[B21 - B2];
ΔR2 = Simplify[R21 - R2];
ΔDBR = Simplify[DBR1 - DBR];
ΔDTB = Simplify[DTB1 - DTB];
ΔDTR = Simplify[DTR1 - DTR];
ΔDTBR = Simplify[DTBR1 - DTBR];
```

In[ ]:=
```
Tdelt1 =
 Simplify[ΔT2 /. {T2 → Teq, R2 → 1 / 2, B2 → 1 / 2, DBR → 0, DTB → 0, DTR → 0, DTBR → 0}]
```

Out[ ]=
$$-\Big(\Big(at\,(2+at)\,c\,(29\,(1+c)+at\,(-81+110\,c))\,\gamma\,\big(9\,ab^2\,\big((1+c)^2+at^2\,(-9+10\,c)+2\,at\,(-4+c+5\,c^2)\big)+2\,\big(-9\,(1+c)\,(-2+c\,(-2+\gamma))+2\,at^2\,(-81+10\,c\,(9+\gamma))+at\,\big(-144-90\,c^2\,(-2+\gamma)+c\,(36+121\,\gamma)\big)\big)+2\,ab\,\big(-9\,(1+c)\,(-2+c\,(-2+\gamma))+2\,at^2\,(-81+10\,c\,(9+\gamma))+at\,\big(-144-90\,c^2\,(-2+\gamma)+c\,(36+121\,\gamma)\big)\big)\big)\Big)\Big/\Big(2\,(1+c+at\,(-9+10\,c))\,\big(9\,ab\,\big((1+c)^2+at^2\,(-9+10\,c)+2\,at\,(-4+c+5\,c^2)\big)-9\,(1+c)\,(-2+c\,(-2+\gamma))+2\,at^2\,(-81+10\,c\,(9+\gamma))+at\,\big(-144-90\,c^2\,(-2+\gamma)+c\,(36+121\,\gamma)\big)\big)\,\big(-9\,(1+c)\,(-2+c\,(-2+\gamma))+2\,at^2\,(-81+10\,c\,(9+\gamma))+at\,\big(-144-90\,c^2\,(-2+\gamma)+c\,(36+121\,\gamma)\big)+ab\,\big(-9\,(1+c)\,(-1+c\,(-1+\gamma))+at^2\,(-81+10\,c\,(9+2\,\gamma))+at\,\big(-72-90\,c^2\,(-1+\gamma)+c\,(18+121\,\gamma)\big)\big)\big)\Big)\Big)$$

In[ ]:=
```
T2plus = Simplify[Teq + Tdelt1]
```

Out[ ]=

$$\frac{1}{22\,(1+c+at\,(-9+10\,c))}\,(29\,(1+c)+at\,(-81+110\,c))\Big(2-\Big(11\,at\,(2+at)\,c\,\gamma\,\Big(9\,ab^2\,\big((1+c)^2+at^2\,(-9+10\,c)+2\,at\,(-4+c+5\,c^2)\big)+2\,\big(-9\,(1+c)\,(-2+c\,(-2+\gamma))+2\,at^2\,(-81+10\,c\,(9+\gamma))+at\,(-144-90\,c^2\,(-2+\gamma)+c\,(36+121\,\gamma))\big)+2\,ab\,\big(-9\,(1+c)\,(-2+c\,(-2+\gamma))+2\,at^2\,(-81+10\,c\,(9+\gamma))+at\,(-144-90\,c^2\,(-2+\gamma)+c\,(36+121\,\gamma))\big)\Big)\Big)\Big/\Big(\Big(9\,ab\,\big((1+c)^2+at^2\,(-9+10\,c)+2\,at\,(-4+c+5\,c^2)\big)-9\,(1+c)\,(-2+c\,(-2+\gamma))+2\,at^2\,(-81+10\,c\,(9+\gamma))+at\,\big(-144-90\,c^2\,(-2+\gamma)+c\,(36+121\,\gamma)\big)\Big)\Big(-9\,(1+c)\,(-2+c\,(-2+\gamma))+2\,at^2\,(-81+10\,c\,(9+\gamma))+at\,\big(-144-90\,c^2\,(-2+\gamma)+c\,(36+121\,\gamma)\big)+ab\,\big(-9\,(1+c)\,(-1+c\,(-1+\gamma))+at^2\,(-81+10\,c\,(9+2\,\gamma))+at\,(-72-90\,c^2\,(-1+\gamma)+c\,(18+121\,\gamma))\big)\Big)\Big)\Big)$$

In[ ]:=
```
Bdelt1 =
  Simplify[ΔB2 /. {T2 → Teq, R2 → 1 / 2, B2 → 1 / 2, DBR → 0, DTB → 0, DTR → 0, DTBR → 0}]
```

Out[ ]=

$$\Big((1+ab)\,c\,\big(9-121\,at-20\,at^2+9\,c+90\,at\,c\big)\,\gamma\,\big(9\,(1+c)\,(-2+c\,(-2+\gamma))-2\,at^2\,(-81+10\,c\,(9+\gamma))+at\,(144+90\,c^2\,(-2+\gamma)-c\,(36+121\,\gamma))\big)\Big)\Big/\Big(4\,\Big(9\,ab\,\big((1+c)^2+at^2\,(-9+10\,c)+2\,at\,(-4+c+5\,c^2)\big)-9\,(1+c)\,(-2+c\,(-2+\gamma))+2\,at^2\,(-81+10\,c\,(9+\gamma))+at\,\big(-144-90\,c^2\,(-2+\gamma)+c\,(36+121\,\gamma)\big)\Big)\Big(-9\,(1+c)\,(-2+c\,(-2+\gamma))+2\,at^2\,(-81+10\,c\,(9+\gamma))+at\,\big(-144-90\,c^2\,(-2+\gamma)+c\,(36+121\,\gamma)\big)+ab\,\big(-9\,(1+c)\,(-1+c\,(-1+\gamma))+at^2\,(-81+10\,c\,(9+2\,\gamma))+at\,(-72-90\,c^2\,(-1+\gamma)+c\,(18+121\,\gamma))\big)\Big)\Big)$$

In[ ]:=
```
B2plus = Bdelt1 + 1 / 2;
```

In[ ]:=
```
Rdelt1 =
  Simplify[ΔR2 /. {T2 → Teq, R2 → 1 / 2, B2 → 1 / 2, DBR → 0, DTB → 0, DTR → 0, DTBR → 0}]
```

Out[ ]= 0

In[ ]:=
```
R2plus = Rdelt1 + 1 / 2;
```

In[ ]:=
```
DBRplus =
  Simplify[ΔDBR /. {T2 → Teq, R2 → 1 / 2, B2 → 1 / 2, DBR → 0, DTB → 0, DTR → 0, DTBR → 0}]
```

Out[ ]=

$$\Big(9\,ab\,(2+ab)\,\big((1+c)^2+at^2\,(-9+10\,c)+2\,at\,(-4+c+5\,c^2)\big)\,\big(-9\,(1+c)\,(-1+c\,(-1+\gamma))+at^2\,(-81+10\,c\,(9+2\,\gamma))+at\,(-72-90\,c^2\,(-1+\gamma)+c\,(18+121\,\gamma))\big)\Big)\Big/\Big(16\,\Big(9\,ab\,\big((1+c)^2+at^2\,(-9+10\,c)+2\,at\,(-4+c+5\,c^2)\big)-9\,(1+c)\,(-2+c\,(-2+\gamma))+2\,at^2\,(-81+10\,c\,(9+\gamma))+at\,\big(-144-90\,c^2\,(-2+\gamma)+c\,(36+121\,\gamma)\big)\Big)\Big(-9\,(1+c)\,(-2+c\,(-2+\gamma))+2\,at^2\,(-81+10\,c\,(9+\gamma))+at\,\big(-144-90\,c^2\,(-2+\gamma)+c\,(36+121\,\gamma)\big)+ab\,\big(-9\,(1+c)\,(-1+c\,(-1+\gamma))+at^2\,(-81+10\,c\,(9+2\,\gamma))+at\,(-72-90\,c^2\,(-1+\gamma)+c\,(18+121\,\gamma))\big)\Big)\Big)$$

In[ ]:= **DTBplus =**
Simplify[ΔDTB /. {T2 → Teq, R2 → 1 / 2, B2 → 1 / 2, DBR → 0, DTB → 0, DTR → 0, DTBR → 0}]

Out[ ]= $-\Big(\Big(9\,at\,(2+at)\,c\,(1+at+c)\,(29\,(1+c)+at\,(-81+110\,c))\,\gamma\,\Big(9\,ab^4\,\big((1+c)^2+at^2\,(-9+10\,c)+2\,at\,(-4+c+5\,c^2)\big)\,\big(-9\,(1+c)\,(-1+c\,(-1+\gamma))+at^2\,(-81+10\,c\,(9+2\,\gamma))+at\,(-72-90\,c^2\,(-1+\gamma)+c\,(18+121\,\gamma))\big)+4\,\big(-9\,(1+c)\,(-2+c\,(-2+\gamma))+2\,at^2\,(-81+10\,c\,(9+\gamma))+at\,(-144-90\,c^2\,(-2+\gamma)+c\,(36+121\,\gamma))\big)^2+8\,ab\,\big(-9\,(1+c)\,(-2+c\,(-2+\gamma))+2\,at^2\,(-81+10\,c\,(9+\gamma))+at\,(-144-90\,c^2\,(-2+\gamma)+c\,(36+121\,\gamma))\big)^2+6\,ab^2\,\big(-9\,(1+c)\,(-2+c\,(-2+\gamma))+2\,at^2\,(-81+10\,c\,(9+\gamma))+at\,(-144-90\,c^2\,(-2+\gamma)+c\,(36+121\,\gamma))\big)^2+2\,ab^3\,\big(-9\,(1+c)\,(-2+c\,(-2+\gamma))+2\,at^2\,(-81+10\,c\,(9+\gamma))+at\,(-144-90\,c^2\,(-2+\gamma)+c\,(36+121\,\gamma))\big)^2\Big)\Big)\Big/\Big(8\,\big(9\,ab\,\big((1+c)^2+at^2\,(-9+10\,c)+2\,at\,(-4+c+5\,c^2)\big)-9\,(1+c)\,(-2+c\,(-2+\gamma))+2\,at^2\,(-81+10\,c\,(9+\gamma))+at\,(-144-90\,c^2\,(-2+\gamma)+c\,(36+121\,\gamma))\big)^2\,\big(-9\,(1+c)\,(-2+c\,(-2+\gamma))+2\,at^2\,(-81+10\,c\,(9+\gamma))+at\,(-144-90\,c^2\,(-2+\gamma)+c\,(36+121\,\gamma))+ab\,\big(-9\,(1+c)\,(-1+c\,(-1+\gamma))+at^2\,(-81+10\,c\,(9+2\,\gamma))+at\,(-72-90\,c^2\,(-1+\gamma)+c\,(18+121\,\gamma))\big)\big)^2\Big)\Big)$

In[ ]:= **DTRplus =**
Simplify[ΔDTR /. {T2 → Teq, R2 → 1 / 2, B2 → 1 / 2, DBR → 0, DTB → 0, DTR → 0, DTBR → 0}]

Out[ ]= $-\Big((9\,ab\,(2+ab)\,at\,(2+at)\,c\,(1+at+c)\,(29\,(1+c)+at\,(-81+110\,c))\,\gamma)\Big/\Big(8\,\big(9\,ab\,\big((1+c)^2+at^2\,(-9+10\,c)+2\,at\,(-4+c+5\,c^2)\big)-9\,(1+c)\,(-2+c\,(-2+\gamma))+2\,at^2\,(-81+10\,c\,(9+\gamma))+at\,(-144-90\,c^2\,(-2+\gamma)+c\,(36+121\,\gamma))\big)\,\big(-9\,(1+c)\,(-2+c\,(-2+\gamma))+2\,at^2\,(-81+10\,c\,(9+\gamma))+at\,(-144-90\,c^2\,(-2+\gamma)+c\,(36+121\,\gamma))+ab\,\big(-9\,(1+c)\,(-1+c\,(-1+\gamma))+at^2\,(-81+10\,c\,(9+2\,\gamma))+at\,(-72-90\,c^2\,(-1+\gamma)+c\,(18+121\,\gamma))\big)\big)\Big)\Big)$

In[ ]:= **DTBRplus = Simplify[**
ΔDTBR /. {T2 → Teq, R2 → 1 / 2, B2 → 1 / 2, DBR → 0, DTB → 0, DTR → 0, DTBR → 0}]

Out[ ]= $\Big(9\,ab\,\big(2+3\,ab+ab^2\big)\,at\,(2+at)\,c^2\,(29\,(1+c)+at\,(-81+110\,c))\,\big(20\,at^3+at^2\,(141-70\,c)-9\,(1+c)^2+at\,(112+22\,c-90\,c^2)\big)\,\gamma^2\,\big(-9\,(1+c)\,(-2+c\,(-2+\gamma))+2\,at^2\,(-81+10\,c\,(9+\gamma))+at\,(-144-90\,c^2\,(-2+\gamma)+c\,(36+121\,\gamma))\big)\Big)\Big/\Big(16\,\big(9\,ab\,\big((1+c)^2+at^2\,(-9+10\,c)+2\,at\,(-4+c+5\,c^2)\big)-9\,(1+c)\,(-2+c\,(-2+\gamma))+2\,at^2\,(-81+10\,c\,(9+\gamma))+at\,(-144-90\,c^2\,(-2+\gamma)+c\,(36+121\,\gamma))\big)^2\,\big(-9\,(1+c)\,(-2+c\,(-2+\gamma))+2\,at^2\,(-81+10\,c\,(9+\gamma))+at\,(-144-90\,c^2\,(-2+\gamma)+c\,(36+121\,\gamma))+ab\,\big(-9\,(1+c)\,(-1+c\,(-1+\gamma))+at^2\,(-81+10\,c\,(9+2\,\gamma))+at\,(-72-90\,c^2\,(-1+\gamma)+c\,(18+121\,\gamma))\big)\big)^2\Big)$

(*now figure out the ΔR for the next generation.*)

In[ ]:=
```
Rdelt2 = Simplify[ΔR2 /. {T2 → T2plus, R2 → R2plus, B2 → B2plus,
      DBR → DBRplus, DTB → DTBplus, DTR → DTRplus, DTBR → DTBRplus}];
```

In[158]:=
```
(*The cell below is the same expression for Rdelt2 as a function of c,
at, ab, and γ. Need to have it written out or MMA won'
 t define the function properly. Expand cell to view.*)


(*Explore Graphically;
R2 is favored in this scenario as long as ab is strong enough*)
```

In[ ]:=
```
Manipulate[Plot[Rdelt2func[c, at, ab, γ], {γ, 0, 1}, PlotRange → Full],
 {c, 0, (at - 2 m - at m + (1 + at) (-1 + m) st)/(at + 2 m + at m + st - m st)},
 {{at, 4}, -1 + (1 + m)/((-1 + m) (-1 + st)), 16}, {ab, 0, 20}]
```

Out[ ]=

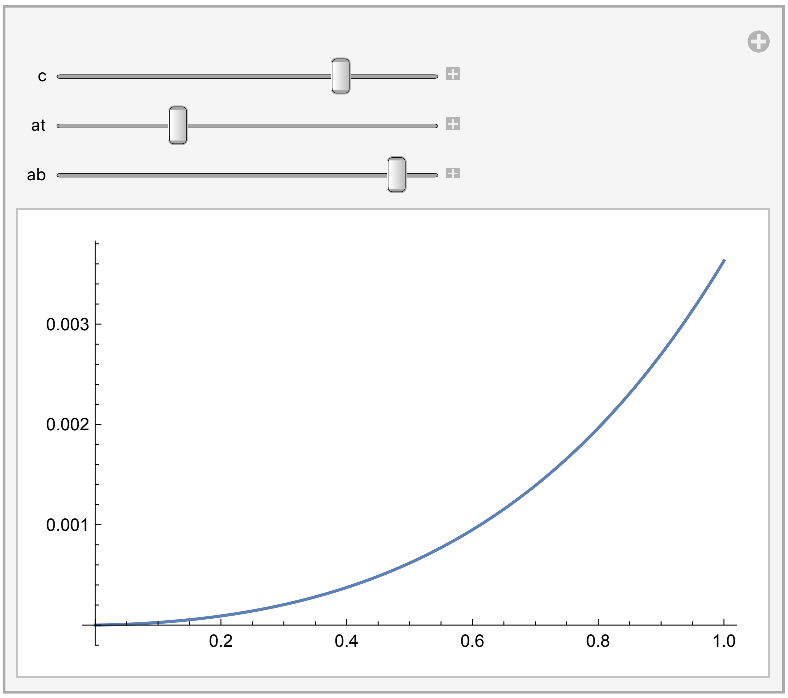

In[ ]:=
```
fig4nofill = Plot[{Rdelt2func[0.45, 5, 16, γ], Rdelt2func[0.45, 5, 12, γ],
    Rdelt2func[0.45, 5, 8, γ], Rdelt2func[0.45, 5, 4, γ]},
   {γ, 0, 1}, PlotStyle → {{ColorData["GrayTones", 8.5 / 10]},
     {ColorData["GrayTones", 6.5 / 10]}, {ColorData["GrayTones", 3.5 / 10]},
     {ColorData["GrayTones", 0 / 10]}}, Frame → True, FrameStyle →
    {{{18, Black, FontFamily → "Times"}, {18, Black, FontFamily → "Times"}},
     {{18, Black, FontFamily → "Times"}, {18, Black, FontFamily → "Times"}}},
   FrameLabel → {Text[Style["Effectiveness of protection, γ",
       FontFamily → "Times", FontSize → 22]],
     Rotate[Text[Style[Row[{"Δ", Subscript["R", "2"]}], FontFamily → "Times",
        FontSize → 22]], -π / 2]}, ImageSize → 576, PlotLegends →
    Placed[LineLegend[{Row[{Style[Subscript["a", "b"], Italic], "=16" }],
       Row[{Style[Subscript["a", "b"], Italic], "=12" }],
       Row[{Style[Subscript["a", "b"], Italic], "=8" }],
       Row[{Style[Subscript["a", "b"], Italic], "=4" }]},
      LabelStyle → {18, FontFamily → "Times"}, LegendLayout → "Column"],
     {0.15, 0.7}], ImagePadding → {{120, 30}, {60, 30}}]
```

Out[ ]=

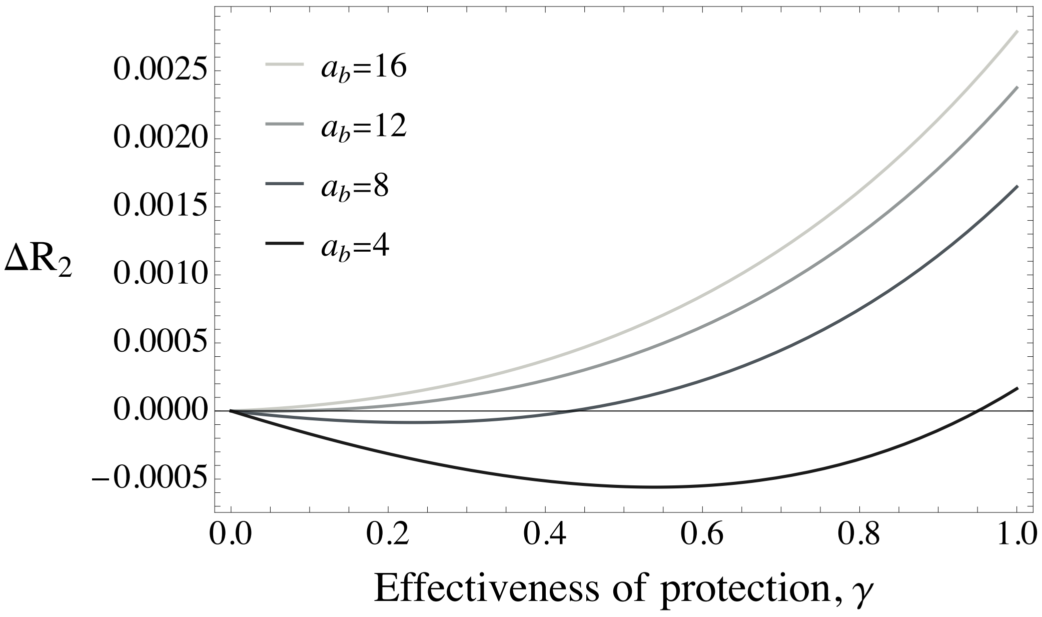

SupportingInformationFile S4

Mathematicacode for: Remodelingand the Evolutionof SexualAutonomyby Mate Choice

Samuel S. Snow[1,2*] and Richard O. Prum[2]
*Corresponding author: samuel.s.snow@gmail.com

[1]Université Toulouse 1 Capitole and Institute for Advanced Studies in Toulouse (IAST), Toulouse, France

[2]Department of Ecology and Evolutionary Biology, Yale University, New Haven, Connecticut, 06511

This code presents a weak selection approximation analysis of the three–locus population–genetic model presented in the main text.

See Table 1 for definitions of alleles and key parameters.

*Note on parametrization: in the main text and Appendices S1–S3, we refer to the mutation parameter as "*u*" to avoid confusion with the subscript m used to denote male genotype frequencies. In the code below, the mutation parameter is denoted "m" and has been left as such to avoid the introduction of errors.

---

1. Develop the recursion equations for the model; Expand this cell to evaluate

In[ ]:=

```
ClearAll[st, sb, at, c, γ, m, init, T1B1R1, T2B1R1, T1B2R1, T2B2R1, T1B1R2,
  T2B1R2, T1B2R2, T2B2R2, genotypes, r, msel, fsel, u, ab, z1, z2, zb, T2, B2,
  R2, DTB, DTR, DBR, DTBR, Mating, s, mgenotypes, T21, B21, R21, DTB1, DTR1,
  DBR1, DTBR1, ΔT2, ΔB2, ΔR2, ΔDTB, ΔDTR, ΔDBR, ΔDTBR, perturb, sr, materec,
  RecTable, MaskProb, ZygoteGenotypes, prob, zyg1, zyg2, zygoteProb];

(*three-
```

```
 locus popgen model of Remodeling without direct costs to coercive mating*)

(*this model uses a probabilistic formulation for how
 females visit and mate at male territories. Females may either
 visit territories randomly or choose territories based on
 the kind of remodeling trait (bower) they see. Once there,
the male may either coerce them into mating there without the
 opportunity to evaluate their display, or the female may choose
 the mate with that male (given that he has not already coerced her)
 by some factor related to his attractivenes.*)

(*There is a locus for male display trait T2/T1,
    remodeling trait (protective bower) B2/B1,
    and female preference for the type of Remodeling trait (bower),
    R2/R1. We make two underlying assumptions in this model: 1)
   that all males will coerce if they have the opportunity, and 2)
 that all females have a preference for attractive T2 males*)

(*parameters:
 (1-st) is the fraction by which males having the T2 allele survive relative
  to Males having the T1 allele. (1-sb) is the fraction by which males
  having the B2 allele survive relative to Males having the B1 allele.
   (1-sr) is the fraction by which females having the
  R2 allele survive relative to females having the R1 allele.
   (a+1) is the factor by which attractive T2 allele males
  have a relative advantage in mating success over T1 males.
   (ab+1) is the factor by which
  females with the R2 allele prefer to visit B2 bowers.
   c is a number between 0 and 1 representing
  the rate of successful forced fertilization of a female,
(or the likelihood of coercive attack) by a male
  without a protective bower (B2).
   γ is a number between 0 and 1 representing the effectiveness of
  protective bowers at reducing a male's ability to coerce. If γ=0,
then there is no effect, and if γ=1, then B2 males are unable to coerce,
and females are free to choose the male based
 on his attractiveness once they are at his territory.

  Lastly, we make an assumption of biased mutation at the T locus. This
 is meant as a proxy for a quantitative, multialleleic trait or
 some other process by which variation is maintained at the T locus,
so that sexual selection is ongoing and we can explore the effects of sexual
  conflict over indirect effects in a simple population-genetic framework*)


(*recombination rate*)
rtb = 1 / 2;
```

```
rbr = 1 / 2;

(*solve for inital genotype frequecies*)

init = Solve[{T1B1R1 + T2B1R1 + T1B2R1 + T2B2R1 + T1B1R2 + T2B1R2 + T1B2R2 + T2B2R2 == 1,
     T2 == T2B1R1 + T2B2R1 + T2B1R2 + T2B2R2, B2 == T1B2R1 + T2B2R1 + T1B2R2 + T2B2R2,
     R2 == T1B1R2 + T2B1R2 + T1B2R2 + T2B2R2, DTB == (T2B2R1 + T2B2R2) - T2 * B2,
     DTR == (T2B1R2 + T2B2R2) - T2 * R2, DBR == (T1B2R2 + T2B2R2) - B2 * R2,
     DTBR == T2B2R2 - T2 * ((T1B2R2 + T2B2R2) - B2 * R2) -
       B2 * ((T2B1R2 + T2B2R2) - T2 * R2) - R2 * ((T2B2R1 + T2B2R2) - T2 * B2) - T2 * B2 * R2},
    {T1B1R1, T2B1R1, T1B2R1, T2B2R1, T1B1R2, T2B1R2, T1B2R2, T2B2R2}];

T1B1R1 = T1B1R1 /. init;
T2B1R1 = T2B1R1 /. init;
T1B2R1 = T1B2R1 /. init;
T2B2R1 = T2B2R1 /. init;
T1B1R2 = T1B1R2 /. init;
T2B1R2 = T2B1R2  /. init;
T1B2R2 = T1B2R2 /. init;
T2B2R2 = T2B2R2 /. init;


(*genotypes vector with initial allele frequencies and linkages*)
genotypes = Flatten[{T1B1R1, T2B1R1, T1B2R1, T2B2R1,
     T1B1R2, T2B1R2, T1B2R2, T2B2R2, T2, B2, R2, DTB, DTR, DBR, DTBR}];

(*set up the table of recombination probabilities that will yield the
 distribution of zygote genotypes resulting from each mated pairing*)

Nloci = 3;
Geno = 2^Nloci;
masklim = 2^(Nloci - 1);
recrate[1] = rtb;
recrate[2] = rbr;

(*Maskprob gives the probability for a particular pattern of
 recombination (a mask),based on given values of recombination
 rates between different loci ("recrate" vector above)*)

MaskProb = Function[{mask, Nloci},
   For[prob = 1; z = 1, z < Nloci, z++,
    If[BitGet[mask, z] == BitGet[mask, z - 1],
     prob *= (1 - recrate[z]),
     prob *= recrate[z]]]];

(*generates the two zygote genotypes (zyg1 and zyg2)
 from two parental genotypes (i and j) for a given mask.*)
```

```
ZygoteGenotypes = Function[{i, j, mask},
   For[zyg1 = 0; zyg2 = 0; k = 0, k < Nloci, k++,
    If[BitGet[mask, k] == 0,
     zyg1 = BitOr[zyg1, (BitAnd[i, (2^k)])];
     zyg2 = BitOr[zyg2, (BitAnd[j, (2^k)])],
     zyg1 = BitOr[zyg1, (BitAnd[j, (2^k)])];
     zyg2 = BitOr[zyg2, (BitAnd[i, (2^k)])]]]];

(*Produce a 3-dimentional matrix with the cells representing
  the probability of producing a zygote genotype (k) from parental
  genotypes i (mother) and j (father). Multiplying this matrix with
  the mating table that gives the proportions of different male-
 female genotype mating combinations will give the
  frequency of zygote genotypes in the new generation
  (after summing across parental genotypes).*)

RecTable = Table[0, {i, Geno}, {j, Geno}, {k, Geno}];
For[i = 0, i < Geno, i++,
 For[j = 0, j < Geno, j++,
  For[mask = 0, mask < masklim, mask++,
   MaskProb[mask, Nloci];
   zygoteProb = (1 / 2) * prob;
   ZygoteGenotypes[i, j, mask];
   RecTable[[(i + 1), (j + 1), (zyg1 + 1)]] += zygoteProb;
   RecTable[[(i + 1), (j + 1), (zyg2 + 1)]] += zygoteProb;]]]

(*uncomment this line to view the recombination table*)
(*MatrixForm[RecTable]*)

(*make a vector that will perform Natural Selection on males,
affecting gene frequencies before mating.*)

msel = Table[i, {i, 1, 8}];
u = Total[genotypes[[{1, 5}]]] + (1 - st) * Total[genotypes[[{2, 6}]]] +
   (1 - sb) * Total[genotypes[[{3, 7}]]] + (1 - sb) * (1 - st) * Total[genotypes[[{4, 8}]]];
msel[[{1, 5}]] = Table[genotypes[[i]] / u, {i, {1, 5}}];
msel[[{2, 6}]] = Table[((1 - st) * genotypes[[i]]) / u, {i, {2, 6}}];
msel[[{3, 7}]] = Table[((1 - sb) * genotypes[[i]]) / u, {i, {3, 7}}];
msel[[{4, 8}]] = Table[((1 - sb) * (1 - st) * genotypes[[i]]) / u, {i, {4, 8}}];

zb = Total[msel[[{1, 2, 5, 6}]]] + (ab + 1) * Total[msel[[{3, 4, 7, 8}]]];

z1 = Total[msel[[{1, 5}]]] * (c + (1 - c) * (1 / (at + 2))) +
   Total[msel[[{2, 6}]]] * (c + (1 - c) * ((at + 1) / (at + 2))) +
   Total[msel[[{3, 7}]]] * (c * (1 - γ) + (1 - c * (1 - γ)) * (1 / (at + 2))) +
   Total[msel[[{4, 8}]]] * (c * (1 - γ) + (1 - c * (1 - γ)) * ((at + 1) / (at + 2)));
```

```
z2 = (Total[msel[[{1, 5}]]] / zb) * (c + (1 - c) * (1 / (at + 2))) +
    (Total[msel[[{2, 6}]]] / zb) * (c + (1 - c) * ((at + 1) / (at + 2))) +
    Total[msel[[{3, 7}]]] * ((1 + ab) / zb) * (c * (1 - γ) + (1 - c * (1 - γ)) * (1 / (at + 2))) +
    Total[msel[[{4, 8}]]] * ((1 + ab) / zb) *
     (c * (1 - γ) + (1 - c * (1 - γ)) * ((at + 1) / (at + 2)));

(*Selection on females resulting from
 cost of the female bower preference, R2*)

fsel = Table[i, {i, 1, 8}];
v = Total[genotypes[[1 ;; 4]]] + (1 - sr) * Total[genotypes[[5 ;; 8]]];
fsel[[1 ;; 4]] = Table[genotypes[[i]] / v, {i, 1, 4}];
fsel[[5 ;; 8]] = Table[((1 - sr) * genotypes[[i]]) / v, {i, 5, 8}];


(*mating probability matrix-what proportion of matings
  will be from each pairing strictly based on genotype frequency,
female choice, coercion, and bower type.*)
(*note that the mating matrix in the code here is
 the transpose of the arrangement presented in Table 2,
with female genotypes as the "rows" and male genotypes as the "columns."
 This is purely organizational; they are exactly equivalent*)

Mating = Table[Table[i, {i, 1, 8}], {j, 1, 8}];

(*for mating with R1 females*)

(*T1B1 males*)
    Mating[[1 ;; 4, {1, 5}]] =
  Table[Table[fsel[[j]] (msel[[i]] (c + (1-c)/(at+2))) / z1, {i, {1, 5}}], {j, 1, 4}];
(*T2B1 males*)
    Mating[[1 ;; 4, {2, 6}]] =
  Table[Table[fsel[[j]] (msel[[i]] (c + ((1-c) (at+1))/(at+2))) / z1, {i, {2, 6}}], {j, 1, 4}];
(*T1B2 males*)
    Mating[[1 ;; 4, {3, 7}]] =
  Table[Table[fsel[[j]] (msel[[i]] (c (1 - γ) + (1-c (1-γ))/(at+2))) / z1, {i, {3, 7}}], {j, 1, 4}];
(*T2B2 males*)
    Mating[[1 ;; 4, {4, 8}]] = Table[
   Table[fsel[[j]] (msel[[i]] (c (1 - γ) + ((1-c (1-γ)) (at+1))/(at+2))) / z1, {i, {4, 8}}], {j, 1, 4}];
```

```
(*for mating with R2 females*)

(*T1B1 males*)
    Mating[[5 ;; 8, {1, 5}]] =
  Table[Table[fsel[[j]] (msel[[i]] (c + (1-c)/(at+2)))/(zb z2), {i, {1, 5}}], {j, 5, 8}];
(*T2B1 males*)
    Mating[[5 ;; 8, {2, 6}]] =
  Table[Table[fsel[[j]] (msel[[i]] (c + ((1-c) (at+1))/(at+2)))/(zb z2), {i, {2, 6}}], {j, 5, 8}];
(*T1B2 males*)
    Mating[[5 ;; 8, {3, 7}]] = Table[
   Table[fsel[[j]] (msel[[i]] (1 + ab) (c (1 - γ) + (1-c (1-γ))/(at+2)))/(zb z2), {i, {3, 7}}], {j, 5, 8}];
(*T2B2 males*)
    Mating[[5 ;; 8, {4, 8}]] = Table[Table[
    fsel[[j]] (msel[[i]] (1 + ab) (c (1 - γ) + ((1-c (1-γ)) (at+1))/(at+2)))/(zb z2), {i, {4, 8}}], {j, 5, 8}];


(*Multiply the mating table by the recombination
 matrix to get the contributions to each zygote genotype
 weighted by the proportions of each mate pairing*)

materec = RecTable * Mating;

(*sum across each element of each 8-tuple for the mate pairings
  across the new matrix to get the final genotype frequencies.*)
genotypes[[1 ;; 8]] =
  Table[Simplify[Total[Flatten[materec[[1 ;; 8, 1 ;; 8, i]]]]], {i, 1, 8}];


(*once genotypes are assigned, some frequency from T2
 alleles are shifted over to T1 alleles due to biased mutation*)

mgenotypes = {1, 2, 3, 4, 5, 6, 7, 8};
mgenotypes[[{2, 4, 6, 8}]] = (1 - m) * genotypes[[{2, 4, 6, 8}]];
mgenotypes[[{1, 3, 5, 7}]] = m * genotypes[[{2, 4, 6, 8}]] + genotypes[[{1, 3, 5, 7}]];

T21 = Total[mgenotypes[[{2, 4, 6, 8}]]];
B21 = Total[mgenotypes[[{3, 4, 7, 8}]]];
R21 = Total[mgenotypes[[5 ;; 8]]];
```

```
DTB1 = Total[mgenotypes[[{4, 8}]]] - T21 * B21;
DTR1 = Total[mgenotypes[[{6, 8}]]] - T21 * R21;
DBR1 = Total[mgenotypes[[{7, 8}]]] - B21 * R21;
DTBR1 = mgenotypes[[8]] - T21 * DBR1 - B21 * DTR1 - R21 * DTB1 - T21 * B21 * R21;
```

In[ ]:=
```
(*get the recursions*)

ΔT2 = T21 - T2;
ΔB2 = B21 - B2;
ΔR2 = R21 - R2;
ΔDTB = DTB1 - DTB;
ΔDTR = DTR1 - DTR;
ΔDBR = DBR1 - DBR;
ΔDTBR = DTBR1 - DTBR;
```

2. Get first-order approximations for the recursions for the allele frequencies

We can approximate the expressions by assuming that the viability costs to the various traits as well as the strength of female preference, male coercion, and mutation are weak. We left the magnitude of γ (the protectiveness of the remodeling trait) unconstrained. We can express those parameters as coefficients of a very small number ζ.

In[ ]:=
```
st = ξ * stapprox;
sb = ξ * sbapprox;
sr = ξ * srapprox;
at =  ξ * atapprox;
ab = ξ * abapprox;
c = ξ * capprox;
m = ξ * mapprox;
```

Get the allele frequency recursions to the first order

In[ ]:=
```
ΔT2approx = Simplify[Normal[Series[ΔT2, {ξ, 0, 1}]]]
ΔB2approx = Simplify[Normal[Series[ΔB2, {ξ, 0, 1}]]]
ΔR2approx = Simplify[Normal[Series[ΔR2, {ξ, 0, 1}]]]
```

Out[ ]=
$$\frac{1}{2}\left(\text{abapprox DTB R2} - \text{DTR srapprox} + \text{atapprox T2} - 2\,\text{mapprox T2} - \text{stapprox T2} - \text{atapprox T2}^2 + \text{stapprox T2}^2 - \text{DTB}\,(\text{sbapprox} + \text{capprox}\,\gamma)\right)\xi$$

Out[ ]= $\frac{1}{2}$ (atapprox DTB – abapprox (–1 + B2) B2 R2 – B2 sbapprox + B2^2 sbapprox – DBR srapprox – DTB stapprox – B2 capprox γ + B2^2 capprox γ) ξ

Out[ ]= $\frac{1}{2}$ (atapprox DTR + abapprox DBR R2 – DBR sbapprox – R2 srapprox + R2^2 srapprox – DTR stapprox – capprox DBR γ) ξ

clean up notation

```
In[ ]:= Clear[at, ab, sr, c, st, sb, m]
Simplify[{ΔT2approx, ΔB2approx, ΔR2approx}] /. {atapprox → at, abapprox → ab,
   srapprox → sr, capprox → c, stapprox → st, sbapprox → sb, mapprox → m, ξ → 1}
```

{$\frac{1}{2}$ (ab DTB R2 – DTR sr + at T2 – 2 m T2 – st T2 – at T2^2 + st T2^2 – DTB (sb + c γ)),
$\frac{1}{2}$ (at DTB – ab (–1 + B2) B2 R2 – B2 sb + B2^2 sb – DBR sr – DTB st – B2 c γ + B2^2 c γ),
$\frac{1}{2}$ (at DTR + ab DBR R2 – DBR sb – R2 sr + R2^2 sr – DTR st – c DBR γ)}

```
(*check simplifications for typesetting*)
```

```
In[ ]:= Simplify[(1/2) ((at - st) (1 - T2) T2 + DTB (ab * R2 - (sb + c * γ)) - DTR * sr - 2 m * T2) ==
   1/2 (ab DTB R2 - DTR sr + at T2 - 2 m T2 - st T2 - at T2^2 + st T2^2 - DTB (sb + c γ))]
```

Out[ ]= True

$$\frac{1}{2}(\mathrm{DTB}\,(\mathrm{ab\,R2}-(c\,\gamma+\mathrm{sb}))+(1-\mathrm{T2})\,\mathrm{T2}\,(\mathrm{at}-\mathrm{st})-\mathrm{DTR\,sr}-2\,m\,\mathrm{T2})$$

```
In[ ]:= Simplify[(1/2) ((ab * R2 - (sb + c * γ)) (1 - B2) B2 + DTB (at - st) - DBR * sr) ==
   1/2 (at DTB - ab (-1 + B2) B2 R2 - B2 sb + B2^2 sb - DBR sr - DTB st - B2 c γ + B2^2 c γ)]
```

Out[ ]= True

$$\frac{1}{2}((1-\mathrm{B2})\,\mathrm{B2}\,(\mathrm{ab\,R2}-(c\,\gamma+\mathrm{sb}))+\mathrm{DTB}\,(\mathrm{at}-\mathrm{st})-\mathrm{DBR\,sr})$$

```
In[ ]:= Simplify[(1/2) (DBR (ab * R2 - (sb + c * γ)) + DTR (at - st) - sr (1 - R2) R2) ==
   1/2 (at DTR + ab DBR R2 - DBR sb - R2 sr + R2^2 sr - DTR st - c DBR γ)]
```

Out[ ]= True

$$\frac{1}{2}(\mathrm{DBR}\,(\mathrm{ab\,R2}-(c\,\gamma+\mathrm{sb}))+\mathrm{DTR}\,(\mathrm{at}-\mathrm{st})-((1-\mathrm{R2})\,\mathrm{R2\,sr}))$$

3. Get approximate solutions for the linkages assuming

weak selection and Quasi-Linkage Equilibrium

Make functions for the recursions of the D' s in terms of a polynomial in a single variable . These will be series of coefficients of a very small number ζ

```
In[]:= fΔDTB = ΔDTB /. {DTB → DTBA0 + DTBA1 * ξ + DTBA2 * ξ^2 + DTBA3 * ξ^3 + DTBA4 * ξ^4,
          DTR → DTRA0 + DTRA1 * ξ + DTRA2 * ξ^2 + DTRA3 * ξ^3 + DTRA4 * ξ^4,
          DBR → DBRA0 + DBRA1 * ξ + DBRA2 * ξ^2 + DBRA3 * ξ^3 + DBRA4 * ξ^4,
          DTBR → DTBRA0 + DTBRA1 * ξ + DTBRA2 * ξ^2 + DTBRA3 * ξ^3 + DTBRA4 * ξ^4};

       fΔDTR = ΔDTR /. {DTB → DTBA0 + DTBA1 * ξ + DTBA2 * ξ^2 + DTBA3 * ξ^3 + DTBA4 * ξ^4,
          DTR → DTRA0 + DTRA1 * ξ + DTRA2 * ξ^2 + DTRA3 * ξ^3 + DTRA4 * ξ^4,
          DBR → DBRA0 + DBRA1 * ξ + DBRA2 * ξ^2 + DBRA3 * ξ^3 + DBRA4 * ξ^4,
          DTBR → DTBRA0 + DTBRA1 * ξ + DTBRA2 * ξ^2 + DTBRA3 * ξ^3 + DTBRA4 * ξ^4};

       fΔDBR = ΔDBR /. {DTB → DTBA0 + DTBA1 * ξ + DTBA2 * ξ^2 + DTBA3 * ξ^3 + DTBA4 * ξ^4,
          DTR → DTRA0 + DTRA1 * ξ + DTRA2 * ξ^2 + DTRA3 * ξ^3 + DTRA4 * ξ^4,
          DBR → DBRA0 + DBRA1 * ξ + DBRA2 * ξ^2 + DBRA3 * ξ^3 + DBRA4 * ξ^4,
          DTBR → DTBRA0 + DTBRA1 * ξ + DTBRA2 * ξ^2 + DTBRA3 * ξ^3 + DTBRA4 * ξ^4};

       fΔDTBR = ΔDTBR /. {DTB → DTBA0 + DTBA1 * ξ + DTBA2 * ξ^2 + DTBA3 * ξ^3 + DTBA4 * ξ^4,
          DTR → DTRA0 + DTRA1 * ξ + DTRA2 * ξ^2 + DTRA3 * ξ^3 + DTRA4 * ξ^4,
          DBR → DBRA0 + DBRA1 * ξ + DBRA2 * ξ^2 + DBRA3 * ξ^3 + DBRA4 * ξ^4,
          DTBR → DTBRA0 + DTBRA1 * ξ + DTBRA2 * ξ^2 + DTBRA3 * ξ^3 + DTBRA4 * ξ^4};
```

again, assume weak selection but leave γ large

```
st = ξ * stapprox;
sb = ξ * sbapprox;
sr = ξ * srapprox;
at =  ξ * atapprox;
ab = ξ * abapprox;
c = ξ * capprox;
m = ξ * mapprox;
```

Get the first (zero-th order) term for each recursion expression

```
In[]:= DTBA0eq = Normal[Simplify[Series[fΔDTB, {ξ, 0, 0}]]]
```

Out[]= $-\frac{\text{DTBA0}}{2}$

```
In[]:= DTRA0eq = Normal[Simplify[Series[fΔDTR, {ξ, 0, 0}]]]
```

Out[]= $-\frac{\text{DTRA0}}{2}$

```
In[]:= DBRA0eq = Normal[Simplify[Series[fΔDBR, {ξ, 0, 0}]]]
```

Out[∘]= $-\frac{\text{DBRA0}}{2}$

```
In[∘]:= DTBRA0eq = Normal[Simplify[Series[fΔDTBR, {ξ, 0, 0}]]]
```

Out[∘]= $-\frac{3\ \text{DTBRA0}}{4}$

```
In[∘]:= Simplify[Solve[{DTBA0eq == 0, DTRA0eq == 0, DBRA0eq == 0, DTBRA0eq == 0},
          {DTBA0, DTRA0, DBRA0, DTBRA0}]]

        {{DTBA0 → 0, DTRA0 → 0, DBRA0 → 0, DTBRA0 → 0}}

        (*all the zeroth order terms are zero!*)
```

Get the second (first-order) terms

```
In[∘]:= DTBA1eq = Normal[Simplify[Series[fΔDTB, {ξ, 0, 1}]]]
```

Out[∘]= $-\frac{\text{DTBA0}}{2}+\frac{1}{4}\big(-2\ \text{DTBA1}-2\ \text{DTBA0 mapprox}+\text{abapprox}\ \big(\text{DBRA0 DTBA0}+\text{B2 DTRA0}-\text{B2}^2\ \text{DTRA0}+\text{DTBA0 R2}-2\ \text{B2 DTBA0 R2}\big)-\text{DTBA0 sbapprox}+2\ \text{B2 DTBA0 sbapprox}-\text{DTBRA0 srapprox}-\text{DTBA0 stapprox}+2\ \text{DTBA0 stapprox T2}+\text{atapprox}\ (\text{DTBA0}-2\ \text{DTBA0 T2})-\text{capprox DTBA0}\ \gamma+2\ \text{B2 capprox DTBA0}\ \gamma\big)\ \xi$

```
In[∘]:= DTRA1eq = Normal[Simplify[Series[fΔDTR, {ξ, 0, 1}]]]
```

Out[∘]= $-\frac{\text{DTRA0}}{2}+\frac{1}{4}\ (\text{abapprox DBRA0 DTRA0}-2\ \text{DTRA1}-2\ \text{DTRA0 mapprox}+\text{abapprox R2}\ (\text{DTBA0}+\text{DTBRA0}-\text{DTBA0 R2})-\text{DTBRA0 sbapprox}-\text{DTRA0 srapprox}+2\ \text{DTRA0 R2 srapprox}-\text{DTRA0 stapprox}+2\ \text{DTRA0 stapprox T2}+\text{atapprox}\ (\text{DTRA0}-2\ \text{DTRA0 T2})-\text{capprox DTBRA0}\ \gamma)\ \xi$

```
In[∘]:= DBRA1eq = Normal[Simplify[Series[fΔDBR, {ξ, 0, 1}]]]
```

Out[∘]= $-\frac{\text{DBRA0}}{2}+\frac{1}{4}\big(-2\ \text{DBRA1}+\text{atapprox DTBRA0}+\text{abapprox}\ \big(\text{DBRA0}^2+(-1+\text{B2})\ \text{B2}\ (-1+\text{R2})\ \text{R2}+\text{DBRA0}\ (\text{R2}-2\ \text{B2 R2})\big)-\text{DBRA0 sbapprox}+2\ \text{B2 DBRA0 sbapprox}-\text{DBRA0 srapprox}+2\ \text{DBRA0 R2 srapprox}-\text{DTBRA0 stapprox}-\text{capprox DBRA0}\ \gamma+2\ \text{B2 capprox DBRA0}\ \gamma\big)\ \xi$

```
In[∘]:= DTBRA1eq = Normal[Simplify[Series[fΔDTBR, {ξ, 0, 1}]]]
```

Out[ ]= $-\frac{3\ \text{DTBRA0}}{4}+\frac{1}{8}\Big(-6\ \text{DTBRA1}-2\ \text{DTBRA0 mapprox}+\text{abapprox}\ \big(\text{B2}^2\ \text{DTRA0}\ (-1+2\ \text{R2})+\text{DBRA0}\ (\text{DTBA0}+2\ \text{DTBRA0}+\text{DTRA0}-2\ \text{B2 DTRA0}-4\ \text{DTBA0 R2})+\text{R2}\ (\text{DTBA0}+\text{DTBRA0}-\text{DTBA0 R2})+\text{B2}\ (\text{DTRA0}-2\ \text{DTRA0 R2}-2\ \text{R2}\ (\text{DTBA0}+\text{DBRA0}-\text{DTBA0 R2}))\big)+2\ \text{DBRA0 DTBA0 sbapprox}-\text{DTBRA0 sbapprox}+2\ \text{B2 DTBRA0 sbapprox}-\text{DTBRA0 srapprox}+2\ \text{DBRA0 DTRA0 srapprox}+2\ \text{DTBRA0 R2 srapprox}-\text{DTBRA0 stapprox}+2\ \text{DTBA0 DTRA0 stapprox}+2\ \text{DTBRA0 stapprox T2}+\text{atapprox}\ (\text{DTBRA0}-2\ \text{DTBA0 DTRA0}-2\ \text{DTBRA0 T2})+2\ \text{capprox DBRA0 DTBA0}\ \gamma-\text{capprox DTBRA0}\ \gamma+2\ \text{B2 capprox DTBRA0}\ \gamma\Big)\ \xi$

Plug in solutions for 0 th order

```
In[ ]:= DTBA1eq = Simplify[DTBA1eq /. {DTBA0 → 0, DTRA0 → 0, DBRA0 → 0, DTBRA0 → 0}]
```

Out[ ]= $-\frac{\text{DTBA1}\ \xi}{2}$

```
In[ ]:= DTRA1eq = Simplify[DTRA1eq /. {DTBA0 → 0, DTRA0 → 0, DBRA0 → 0, DTBRA0 → 0}]
```

Out[ ]= $-\frac{\text{DTRA1}\ \xi}{2}$

```
In[ ]:= DBRA1eq = Simplify[DBRA1eq /. {DTBA0 → 0, DTRA0 → 0, DBRA0 → 0, DTBRA0 → 0}]
```

Out[ ]= $\frac{1}{4}\ (-2\ \text{DBRA1}+\text{abapprox}\ (-1+\text{B2})\ \text{B2}\ (-1+\text{R2})\ \text{R2})\ \xi$

```
In[ ]:= DTBRA1eq = Simplify[DTBRA1eq /. {DTBA0 → 0, DTRA0 → 0, DBRA0 → 0, DTBRA0 → 0}]
```

Out[ ]= $-\frac{3\ \text{DTBRA1}\ \xi}{4}$

Solve for 1 st order terms

```
In[ ]:= Simplify[Solve[{DTBA1eq == 0, DTRA1eq == 0, DBRA1eq == 0, DTBRA1eq == 0},
  {DTBA1, DTRA1, DBRA1, DTBRA1}]]
```

$\left\{\left\{\text{DTBA1}\to 0,\ \text{DTRA1}\to 0,\ \text{DBRA1}\to \frac{1}{2}\ \text{abapprox}\ (-1+\text{B2})\ \text{B2}\ (-1+\text{R2})\ \text{R2},\ \text{DTBRA1}\to 0\right\}\right\}$

```
(*the only first-order term is fisherian sexual selection on the bower trait*)

(*need to keep going to uncover the dynamimcs of interest*)
```

Get the third (second – order) terms

```
In[ ]:= DTBA2eq = Normal[Simplify[Series[fΔDTB, {ξ, 0, 2}]]];

DTRA2eq = Normal[Simplify[Series[fΔDTR, {ξ, 0, 2}]]];

DBRA2eq = Normal[Simplify[Series[fΔDBR, {ξ, 0, 2}]]];

DTBRA2eq = Normal[Simplify[Series[fΔDTBR, {ξ, 0, 2}]]];
```

Plug in solutions for 0 th and first order

```
In[•]:= DTBA2eq = Simplify[DTBA2eq /. {DTBA0 → 0, DTRA0 → 0, DBRA0 → 0, DTBRA0 → 0, DTBA1 → 0,
          DTRA1 → 0, DBRA1 → 1/2 abapprox (-1 + B2) B2 (-1 + R2) R2, DTBRA1 → 0}]
```

Out[•]= $-\frac{1}{2}$ (DTBA2 + atapprox B2 capprox T2 (-1 + B2 + T2 - B2 T2) γ) ξ^2

```
In[•]:= DTRA2eq = Simplify[DTRA2eq /. {DTBA0 → 0, DTRA0 → 0, DBRA0 → 0, DTBRA0 → 0, DTBA1 → 0,
          DTRA1 → 0, DBRA1 → 1/2 abapprox (-1 + B2) B2 (-1 + R2) R2, DTBRA1 → 0}]
```

Out[•]= $-\frac{\text{DTRA2}\ \xi^2}{2}$

```
In[•]:= DBRA2eq = Simplify[DBRA2eq /. {DTBA0 → 0, DTRA0 → 0, DBRA0 → 0, DTBRA0 → 0, DTBA1 → 0,
          DTRA1 → 0, DBRA1 → 1/2 abapprox (-1 + B2) B2 (-1 + R2) R2, DTBRA1 → 0}]
```

Out[•]= $-\frac{1}{8}$ (4 DBRA2 + abapprox (-1 + B2) B2 (-1 + R2) R2 (-abapprox R2 + 2 abapprox B2 (1 + R2) +
3 (sbapprox - 2 B2 sbapprox + srapprox - 2 R2 srapprox + (1 - 2 B2) capprox γ))) ξ^2

```
In[•]:= DTBRA2eq =
        Simplify[DTBRA2eq /. {DTBA0 → 0, DTRA0 → 0, DBRA0 → 0, DTBRA0 → 0, DTBA1 → 0,
          DTRA1 → 0, DBRA1 → 1/2 abapprox (-1 + B2) B2 (-1 + R2) R2, DTBRA1 → 0}]
```

Out[•]= $-\frac{3\ \text{DTBRA2}\ \xi^2}{4}$

Solve for second-order terms

```
In[•]:= Simplify[Solve[{DTBA2eq == 0, DTRA2eq == 0, DBRA2eq == 0, DTBRA2eq == 0},
          {DTBA2, DTRA2, DBRA2, DTBRA2}]]
```

{{DTBA2 → atapprox (-1 + B2) B2 capprox (-1 + T2) T2 γ,
DTRA2 → 0, DBRA2 → $-\frac{1}{4}$ abapprox (-1 + B2) B2 (-1 + R2) R2
(-abapprox R2 + 2 abapprox B2 (1 + R2) + 3 (sbapprox - 2 B2 sbapprox +
srapprox - 2 R2 srapprox + (1 - 2 B2) capprox γ)), DTBRA2 → 0}}

Get the fourth (third-order) terms

```
In[•]:= DTBA3eq = Normal[Simplify[Series[fΔDTB, {ξ, 0, 3}]]];

        DTRA3eq = Normal[Simplify[Series[fΔDTR, {ξ, 0, 3}]]];

        DBRA3eq = Normal[Simplify[Series[fΔDBR, {ξ, 0, 3}]]];
```

```
In[•]:= DTBRA3eq = Normal[Simplify[Series[fΔDTBR, {ξ, 0, 3}]]];
```

Plug in solutions for the 0 th, first and second order

```
In[•]:= DTBA3eq = Simplify[DTBA3eq /. {DTBA0 → 0, DTRA0 → 0, DBRA0 → 0, DTBRA0 → 0,
        DTBA1 → 0, DTRA1 → 0, DBRA1 → 1/2 abapprox (-1 + B2) B2 (-1 + R2) R2,
        DTBRA1 → 0, DTBA2 → atapprox (-1 + B2) B2 capprox (-1 + T2) T2 γ,
        DTRA2 → 0, DBRA2 → -1/4 abapprox (-1 + B2) B2 (-1 + R2) R2
          (-abapprox R2 + 2 abapprox B2 (1 + R2) + 3 (sbapprox - 2 B2 sbapprox +
              srapprox - 2 R2 srapprox + (1 - 2 B2) capprox γ)), DTBRA2 → 0}];

DTRA3eq = Simplify[DTRA3eq /. {DTBA0 → 0, DTRA0 → 0, DBRA0 → 0, DTBRA0 → 0,
        DTBA1 → 0, DTRA1 → 0, DBRA1 → 1/2 abapprox (-1 + B2) B2 (-1 + R2) R2,
        DTBRA1 → 0, DTBA2 → atapprox (-1 + B2) B2 capprox (-1 + T2) T2 γ,
        DTRA2 → 0, DBRA2 → -1/4 abapprox (-1 + B2) B2 (-1 + R2) R2
          (-abapprox R2 + 2 abapprox B2 (1 + R2) + 3 (sbapprox - 2 B2 sbapprox +
              srapprox - 2 R2 srapprox + (1 - 2 B2) capprox γ)), DTBRA2 → 0}];

DBRA3eq = Simplify[DBRA3eq /. {DTBA0 → 0, DTRA0 → 0, DBRA0 → 0, DTBRA0 → 0,
        DTBA1 → 0, DTRA1 → 0, DBRA1 → 1/2 abapprox (-1 + B2) B2 (-1 + R2) R2,
        DTBRA1 → 0, DTBA2 → atapprox (-1 + B2) B2 capprox (-1 + T2) T2 γ,
        DTRA2 → 0, DBRA2 → -1/4 abapprox (-1 + B2) B2 (-1 + R2) R2
          (-abapprox R2 + 2 abapprox B2 (1 + R2) + 3 (sbapprox - 2 B2 sbapprox +
              srapprox - 2 R2 srapprox + (1 - 2 B2) capprox γ)), DTBRA2 → 0}];

DTBRA3eq =
  Simplify[DTBRA3eq /. {DTBA0 → 0, DTRA0 → 0, DBRA0 → 0, DTBRA0 → 0, DTBA1 → 0,
        DTRA1 → 0, DBRA1 → 1/2 abapprox (-1 + B2) B2 (-1 + R2) R2, DTBRA1 → 0,
        DTBA2 → atapprox (-1 + B2) B2 capprox (-1 + T2) T2 γ,
        DTRA2 → 0, DBRA2 → -1/4 abapprox (-1 + B2) B2 (-1 + R2) R2
          (-abapprox R2 + 2 abapprox B2 (1 + R2) + 3 (sbapprox - 2 B2 sbapprox +
              srapprox - 2 R2 srapprox + (1 - 2 B2) capprox γ)), DTBRA2 → 0}];
```

Solve for third-order terms

```
In[•]:= Simplify[Solve[{DTBA3eq == 0, DTRA3eq == 0, DBRA3eq == 0, DTBRA3eq == 0},
   {DTBA3, DTRA3, DBRA3, DTBRA3}]]
```

```
{{DTBA3 → -1/2 atapprox (-1 + B2) B2 capprox (-1 + T2) T2 γ
    (4 mapprox - 3 abapprox R2 + 6 abapprox B2 R2 + 3 sbapprox - 6 B2 sbapprox +
      3 stapprox - 6 stapprox T2 + atapprox (-2 + 6 T2) + capprox (4 + γ - 6 B2 γ)),
  DTRA3 → -2 abapprox atapprox (-1 + B2) B2 capprox (-1 + R2) R2 (-1 + T2) T2 γ,
  DBRA3 → 1/8 abapprox (-1 + B2) B2 (-1 + R2) R2
    (abapprox^2 (R2^2 - B2 R2 (3 + 7 R2) + B2^2 (4 + 7 R2 + 7 R2^2)) +
      3 (1 - 8 B2 + 10 B2^2) sbapprox^2 + 3 srapprox^2 - 24 R2 srapprox^2 + 30 R2^2 srapprox^2 -
      6 atapprox capprox γ + 12 atapprox B2 capprox γ + 6 capprox^2 γ -
      12 B2 capprox^2 γ + 10 capprox srapprox γ - 20 B2 capprox srapprox γ -
      20 capprox R2 srapprox γ + 40 B2 capprox R2 srapprox γ + 12 atapprox capprox T2 γ -
      24 atapprox B2 capprox T2 γ + 3 capprox^2 γ^2 - 24 B2 capprox^2 γ^2 + 30 B2^2 capprox^2 γ^2 +
      2 sbapprox (5 (-1 + 2 B2) (-1 + 2 R2) srapprox + 6 (1 - 5 B2 + 5 B2^2) capprox γ) -
      2 abapprox (2 B2^2 (4 + 7 R2) (sbapprox + capprox γ) +
          R2 (3 sbapprox + (3 - 5 R2) srapprox + 3 capprox γ) -
          B2 ((5 + 14 R2) sbapprox + (3 - 10 R2^2) srapprox + capprox (5 + 14 R2) γ))),
  DTBRA3 → 2/3 abapprox atapprox B2 (1 - 3 B2 + 2 B2^2) capprox (-1 + R2) R2 (-1 + T2) T2 γ}}
```

Bring all the terms together and clean up notation

```
In[•]:= Clear[at, ab, sr, c, st, sb, m, B2, R2, Teq, T2]

In[•]:= DTBapprox = Simplify[atapprox (-1 + B2) B2 capprox (-1 + T2) T2 γ +
      -1/2 atapprox (-1 + B2) B2 capprox (-1 + T2) T2 γ
       (4 mapprox - 3 abapprox R2 + 6 abapprox B2 R2 + 3 sbapprox - 6 B2 sbapprox +
         3 stapprox - 6 stapprox T2 + atapprox (-2 + 6 T2) + capprox (4 + γ - 6 B2 γ)) /.
    {atapprox → at, abapprox → ab, srapprox → sr, capprox → c,
     stapprox → st, sbapprox → sb, mapprox → m}]

Out[•]= -1/2 at (-1 + B2) B2 c (-1 + T2) T2 γ (-2 + 4 m - 3 ab R2 +
    6 ab B2 R2 + 3 sb - 6 B2 sb + 3 st - 6 st T2 + at (-2 + 6 T2) + c (4 + γ - 6 B2 γ))

-1/2 at (-1 + B2) B2 c (-1 + T2) T2 γ (-2 + 4 m - 3 ab R2 +
    6 ab B2 R2 + 3 sb - 6 B2 sb + 3 st - 6 st T2 + at (-2 + 6 T2) + c (4 + γ - 6 B2 γ))

In[•]:= DTRapprox =
  Simplify[-2 abapprox atapprox (-1 + B2) B2 capprox (-1 + R2) R2 (-1 + T2) T2 γ /.
    {atapprox → at, abapprox → ab, srapprox → sr,
     capprox → c, stapprox → st, sbapprox → sb, mapprox → m}]

Out[•]= -2 ab at (-1 + B2) B2 c (-1 + R2) R2 (-1 + T2) T2 γ

-2 ab at (-1 + B2) B2 c (-1 + R2) R2 (-1 + T2) T2 γ
```

```
In[ ]:= DBRapprox =
  Simplify[1/2 abapprox (-1 + B2) B2 (-1 + R2) R2 + -1/4 abapprox (-1 + B2) B2 (-1 + R2)
      R2 (-abapprox R2 + 2 abapprox B2 (1 + R2) + 3 (sbapprox - 2 B2 sbapprox +
           srapprox - 2 R2 srapprox + (1 - 2 B2) capprox γ)) + 1/8 abapprox (-1 + B2)
      B2 (-1 + R2) R2 (abapprox^2 (R2^2 - B2 R2 (3 + 7 R2) + B2^2 (4 + 7 R2 + 7 R2^2)) +
        3 (1 - 8 B2 + 10 B2^2) sbapprox^2 + 3 srapprox^2 - 24 R2 srapprox^2 + 30 R2^2 srapprox^2 -
        6 atapprox capprox γ + 12 atapprox B2 capprox γ + 6 capprox^2 γ - 12 B2 capprox^2 γ +
        10 capprox srapprox γ - 20 B2 capprox srapprox γ - 20 capprox R2 srapprox γ +
        40 B2 capprox R2 srapprox γ + 12 atapprox capprox T2 γ - 24 atapprox B2
         capprox T2 γ + 3 capprox^2 γ^2 - 24 B2 capprox^2 γ^2 + 30 B2^2 capprox^2 γ^2 +
        2 sbapprox (5 (-1 + 2 B2) (-1 + 2 R2) srapprox + 6 (1 - 5 B2 + 5 B2^2) capprox γ) -
        2 abapprox (2 B2^2 (4 + 7 R2) (sbapprox + capprox γ) +
           R2 (3 sbapprox + (3 - 5 R2) srapprox + 3 capprox γ) -
           B2 ((5 + 14 R2) sbapprox + (3 - 10 R2^2) srapprox + capprox (5 + 14 R2) γ))) /.
    {atapprox → at, abapprox → ab, srapprox → sr, capprox → c,
     stapprox → st, sbapprox → sb, mapprox → m}]

Out[ ]= 1/8 ab (-1 + B2) B2 (-1 + R2) R2
   (4 + 2 ab R2 - 4 ab B2 (1 + R2) + ab^2 (R2^2 - B2 R2 (3 + 7 R2) + B2^2 (4 + 7 R2 + 7 R2^2)) +
     3 (1 - 8 B2 + 10 B2^2) sb^2 + 3 sr^2 - 24 R2 sr^2 + 30 R2^2 sr^2 - 6 at c γ +
     12 at B2 c γ + 6 c^2 γ - 12 B2 c^2 γ + 10 c sr γ - 20 B2 c sr γ - 20 c R2 sr γ +
     40 B2 c R2 sr γ + 12 at c T2 γ - 24 at B2 c T2 γ + 3 c^2 γ^2 - 24 B2 c^2 γ^2 +
     30 B2^2 c^2 γ^2 - 6 (sb - 2 B2 sb + sr - 2 R2 sr + (1 - 2 B2) c γ) +
     2 sb (5 (-1 + 2 B2) (-1 + 2 R2) sr + 6 (1 - 5 B2 + 5 B2^2) c γ) -
     2 ab (2 B2^2 (4 + 7 R2) (sb + c γ) + R2 (3 sb + (3 - 5 R2) sr + 3 c γ) -
        B2 ((5 + 14 R2) sb + (3 - 10 R2^2) sr + c (5 + 14 R2) γ)))
```


```
1/8 ab (-1 + B2) B2 (-1 + R2) R2
   (4 + 2 ab R2 - 4 ab B2 (1 + R2) + ab^2 (R2^2 - B2 R2 (3 + 7 R2) + B2^2 (4 + 7 R2 + 7 R2^2)) +
     3 (1 - 8 B2 + 10 B2^2) sb^2 + 3 sr^2 - 24 R2 sr^2 + 30 R2^2 sr^2 - 6 at c γ +
     12 at B2 c γ + 6 c^2 γ - 12 B2 c^2 γ + 10 c sr γ - 20 B2 c sr γ - 20 c R2 sr γ +
     40 B2 c R2 sr γ + 12 at c T2 γ - 24 at B2 c T2 γ + 3 c^2 γ^2 - 24 B2 c^2 γ^2 +
     30 B2^2 c^2 γ^2 - 6 (sb - 2 B2 sb + sr - 2 R2 sr + (1 - 2 B2) c γ) +
     2 sb (5 (-1 + 2 B2) (-1 + 2 R2) sr + 6 (1 - 5 B2 + 5 B2^2) c γ) -
     2 ab (2 B2^2 (4 + 7 R2) (sb + c γ) + R2 (3 sb + (3 - 5 R2) sr + 3 c γ) -
        B2 ((5 + 14 R2) sb + (3 - 10 R2^2) sr + c (5 + 14 R2) γ)))
```


```
In[ ]:= DTBRapprox =
  Simplify[2/3 abapprox atapprox B2 (1 - 3 B2 + 2 B2^2) capprox (-1 + R2) R2 (-1 + T2) T2 γ /.
    {atapprox → at, abapprox → ab, srapprox → sr,
     capprox → c, stapprox → st, sbapprox → sb, mapprox → m}]
```

Out[*]= $\frac{2}{3}$ ab at B2 $(1 - 3\,B2 + 2\,B2^2)$ c (-1 + R2) R2 (-1 + T2) T2 γ

$\frac{2}{3}$ ab at B2 $(1 - 3\,B2 + 2\,B2^2)$ c (-1 + R2) R2 (-1 + T2) T2 γ

```
(*check simplifications for typesetting*)

(*second-order coefficient for DBR*)

Simplify[-1/4 abapprox (-1 + B2) B2 (-1 + R2) R2 (-abapprox R2 + 2 abapprox B2 (1 + R2) +
      3 (sbapprox - 2 B2 sbapprox + srapprox - 2 R2 srapprox + (1 - 2 B2) capprox γ)) /.
   {atapprox → at, abapprox → ab, srapprox → sr, capprox → c,
    stapprox → st, sbapprox → sb, mapprox → m}]
```

Out[*]= $-\frac{1}{4}$ ab (-1 + B2) B2 (-1 + R2) R2
(-ab R2 + 2 ab B2 (1 + R2) + 3 (sb - 2 B2 sb + sr - 2 R2 sr + (1 - 2 B2) c γ))

```
In[*]:= Simplify[-1/4 ab (-1 + B2) B2 (-1 + R2) R2
     (-ab R2 + 2 ab B2 (1 + R2) + 3 (sb - 2 B2 sb + sr - 2 R2 sr + (1 - 2 B2) c γ)) ==
    1/2 ab (-1 + B2) B2 (-1 + R2) R2
     ((1 / 2) (ab (R2 - 2 B2 (1 + R2)) - 3 ((1 - 2 B2) (sb + c * γ) + sr (1 - 2 R2)))) ]
```

Out[*]= True

$$\frac{1}{2}\,(\mathrm{ab}\,(\mathrm{R2} - 2\,\mathrm{B2}\,(\mathrm{R2}+1)) - 3\,((1 - 2\,\mathrm{B2})\,(c\,\gamma + \mathrm{sb}) + (1 - 2\,\mathrm{R2})\,\mathrm{sr}))$$

```
(*pull out the third-order coefficient for DBR*)

In[*]:= Simplify[1/8 abapprox (-1 + B2) B2 (-1 + R2)
     R2 (abapprox^2 (R2^2 - B2 R2 (3 + 7 R2) + B2^2 (4 + 7 R2 + 7 R2^2)) +
        3 (1 - 8 B2 + 10 B2^2) sbapprox^2 + 3 srapprox^2 - 24 R2 srapprox^2 + 30 R2^2 srapprox^2 -
        6 atapprox capprox γ + 12 atapprox B2 capprox γ + 6 capprox^2 γ -
        12 B2 capprox^2 γ + 10 capprox srapprox γ - 20 B2 capprox srapprox γ -
        20 capprox R2 srapprox γ + 40 B2 capprox R2 srapprox γ + 12 atapprox capprox T2 γ -
        24 atapprox B2 capprox T2 γ + 3 capprox^2 γ^2 - 24 B2 capprox^2 γ^2 + 30 B2^2 capprox^2 γ^2 +
        2 sbapprox (5 (-1 + 2 B2) (-1 + 2 R2) srapprox + 6 (1 - 5 B2 + 5 B2^2) capprox γ) -
        2 abapprox (2 B2^2 (4 + 7 R2) (sbapprox + capprox γ) +
           R2 (3 sbapprox + (3 - 5 R2) srapprox + 3 capprox γ) -
           B2 ((5 + 14 R2) sbapprox + (3 - 10 R2^2) srapprox + capprox (5 + 14 R2) γ))) /.
   {atapprox → at, abapprox → ab, srapprox → sr, capprox → c,
    stapprox → st, sbapprox → sb, mapprox → m}]
```

Out[ ]= $\frac{1}{8}$ ab (-1 + B2) B2 (-1 + R2) R2
(ab^2 (R2^2 - B2 R2 (3 + 7 R2) + B2^2 (4 + 7 R2 + 7 R2^2)) + 3 (1 - 8 B2 + 10 B2^2) sb^2 + 3 sr^2 -
24 R2 sr^2 + 30 R2^2 sr^2 - 6 at c γ + 12 at B2 c γ + 6 c^2 γ - 12 B2 c^2 γ + 10 c sr γ -
20 B2 c sr γ - 20 c R2 sr γ + 40 B2 c R2 sr γ + 12 at c T2 γ - 24 at B2 c T2 γ + 3 c^2 γ^2 -
24 B2 c^2 γ^2 + 30 B2^2 c^2 γ^2 + 2 sb (5 (-1 + 2 B2) (-1 + 2 R2) sr + 6 (1 - 5 B2 + 5 B2^2) c γ) -
2 ab (2 B2^2 (4 + 7 R2) (sb + c γ) + R2 (3 sb + (3 - 5 R2) sr + 3 c γ) -
B2 ((5 + 14 R2) sb + (3 - 10 R2^2) sr + c (5 + 14 R2) γ)))

```
In[ ]:= Simplify[(1 / 4) (ab^2 (R2^2 - B2 * R2 (3 + 7 R2) + B2^2 * (4 + 7 * R2 (1 + R2))) +
      3 sb^2 (1 - 2 B2 (4 - 5 B2)) + 3 sr^2 (1 - 2 R2 (4 - 5 R2)) -
      c * γ ((1 - 2 B2) (6 at (1 - 2 T2) - 10 sr (1 - 2 R2) - 6 c) - 3 c * γ (1 - 2 B2 (4 - 5 B2))) +
      2 sb (5 (1 - 2 B2) (1 - 2 R2) sr + 6 c * γ (1 - 5 B2 (1 - B2))) -
      2 ab ((sb + c * γ) (B2 (8 B2 - 5) + R2 (3 - 14 B2 (1 - B2))) +
         sr (B2 (10 R2^2 - 3) + R2 (3 - 5 R2)))) ==
   (1 / 4) (ab^2 (R2^2 - B2 R2 (3 + 7 R2) + B2^2 (4 + 7 R2 + 7 R2^2)) + 3 (1 - 8 B2 + 10 B2^2) sb^2 +
      3 sr^2 - 24 R2 sr^2 + 30 R2^2 sr^2 - 6 at c γ + 12 at B2 c γ + 6 c^2 γ - 12 B2 c^2 γ + 10 c sr γ -
      20 B2 c sr γ - 20 c R2 sr γ + 40 B2 c R2 sr γ + 12 at c T2 γ - 24 at B2 c T2 γ + 3 c^2 γ^2 -
      24 B2 c^2 γ^2 + 30 B2^2 c^2 γ^2 + 2 sb (5 (-1 + 2 B2) (-1 + 2 R2) sr + 6 (1 - 5 B2 + 5 B2^2) c γ) -
      2 ab (2 B2^2 (4 + 7 R2) (sb + c γ) + R2 (3 sb + (3 - 5 R2) sr + 3 c γ) -
         B2 ((5 + 14 R2) sb + (3 - 10 R2^2) sr + c (5 + 14 R2) γ)))]
```

Out[ ]= True

$$\frac{1}{4}\left(ab^2\left(B2^2 (7 (R2+1) R2+4) - B2\, R2 (7 R2+3) + R2^2\right) - 2\, ab\left(((3-14 B2 (1-B2)) R2 + (8 B2-5) B2)(c\gamma + sb) + sr\left(B2\left(10 R2^2-3\right) + R2 (3-5 R2)\right)\right) - c\gamma\left((1-2 B2)(6\, at (1-2 T2) - 6 c - 10 (1-2 R2) sr) - 3 (1-2 B2 (4-5 B2)) c\gamma\right) + 2\, sb (6 (1-5 B2 (1-B2)) c\gamma + 5 (1-2 B2)(1-2 R2) sr) + 3 (1-2 B2 (4-5 B2)) sb^2 + 3 (1-2 R2 (4-5 R2)) sr^2\right)$$

```
(*third-order term for DTB*)
```

```
In[ ]:= Simplify[
   4 m - 3 ab R2 + 6 ab B2 R2 + 3 sb - 6 B2 sb + 3 st - 6 st T2 + at (-2 + 6 T2) + c (4 + γ - 6 B2 γ) ==
    3 ((1 - 2 B2) (sb - ab * R2) + (1 - 2 T2) st) - 2 at (1 - 3 T2) + c (4 + γ (1 - 6 B2)) + 4 m]
```

Out[ ]= True

$$-\frac{1}{2}\left(3 ((1-2 B2)(sb - ab\, R2) + st (1-2 T2)) - 2\, at (1-3 T2) + c ((1-6 B2)\gamma + 4) + 4 m\right)$$

```
(*expression for DTBR*)
```

```
In[ ]:= Simplify[2/3 ab at B2 (1 - 3 B2 + 2 B2^2) c (-1 + R2) R2 (-1 + T2) T2 γ ==
    (2 / 3) * ab * at * c * γ * (1 - T2) T2 (1 - R2) R2 (1 - B2) B2 (1 - 2 B2)]
```

Out[ ]= True

Visual inspection o f the linkage expressions

```
(*for consistency with weak selection assumptions,
find a set of small parameter values that still
 meets the criteria for an internal T2 equilibrium*)

(*The T2=Teq equilibrium is stable as long as at >
  -1+(1+m)/((-1+m) (-1+st)) and 0 < c <(at-2 m-at m+(1+at) (-1+m) st)/(at+2 m+at m+st-m st).*)
```

In[ ]:=
```
Manipulate[{-1 + (1 + m)/((-1 + m) (-1 + st)), (at - 2 m - at m + (1 + at) (-1 + m) st)/(at + 2 m + at m + st - m st)},
 {{m, 0.0001}, 0, 1}, {{st, 0.001}, 0, 1}, {{at, 0.002}, -1 + (1 + m)/((-1 + m) (-1 + st)), 30}]
```

Out[ ]=

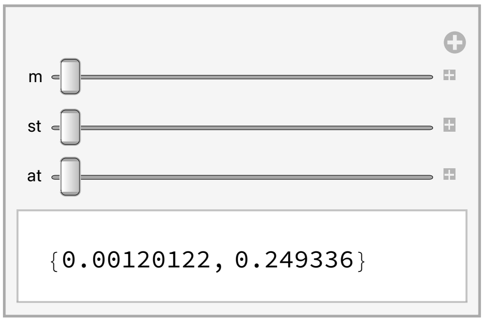

In[160]:=
```
ClearAll[m, st, c, at, ab, sb, sr, T2, R2, B2]
```

In[162]:=
```
Teq = (1 - m + (2 (1+c+at c) m)/(st+c st+at (-1+c+st)))/(1 + m) /. {m → 0.0001, st → 0.001, c → 0.001, at → 0.002}
```

Out[162]=
```
0.798614
```

```
(*look at expression for DBR*)
```

In[ ]:=
```
Manipulate[Plot[(1/8 ab (-1 + B2) B2 (-1 + R2) R2
    (4 + 2 ab R2 - 4 ab B2 (1 + R2) + ab^2 (R2^2 - B2 R2 (3 + 7 R2) + B2^2 (4 + 7 R2 + 7 R2^2)) +
      3 (1 - 8 B2 + 10 B2^2) sb^2 + 3 sr^2 - 24 R2 sr^2 + 30 R2^2 sr^2 - 6 at c γ +
      12 at B2 c γ + 6 c^2 γ - 12 B2 c^2 γ + 10 c sr γ - 20 B2 c sr γ - 20 c R2 sr γ +
      40 B2 c R2 sr γ + 12 at c T2 γ - 24 at B2 c T2 γ + 3 c^2 γ^2 - 24 B2 c^2 γ^2 +
      30 B2^2 c^2 γ^2 - 6 (sb - 2 B2 sb + sr - 2 R2 sr + (1 - 2 B2) c γ) +
      2 sb (5 (-1 + 2 B2) (-1 + 2 R2) sr + 6 (1 - 5 B2 + 5 B2^2) c γ) -
      2 ab (2 B2^2 (4 + 7 R2) (sb + c γ) + R2 (3 sb + (3 - 5 R2) sr + 3 c γ) -
        B2 ((5 + 14 R2) sb + (3 - 10 R2^2) sr + c (5 + 14 R2) γ))) /.
   {sr → 0.00001, st → 0.001, sb → 0.001, m → 0.0001,
    T2 → (1 - m + (2 (1+c+at c) m)/(st+c st+at (-1+c+st)))/(1 + m) /. {m → 0.0001, st → 0.001}}),
  {γ, 0, 1}, PlotRange → Full], {{R2, 0.5},
  0,
  1}, {{B2, 0.5},
  0,
  1}, {c,
  0.001,
  (at - 2 m - at m + (1 + at) (-1 + m) st)/(at + 2 m + at m + st - m st) /.
   {m → 0.0001, st → 0.001}},
 {{at, 0.002}, 0, 16}, {ab, 0, 20}]
```

Out[ ]=

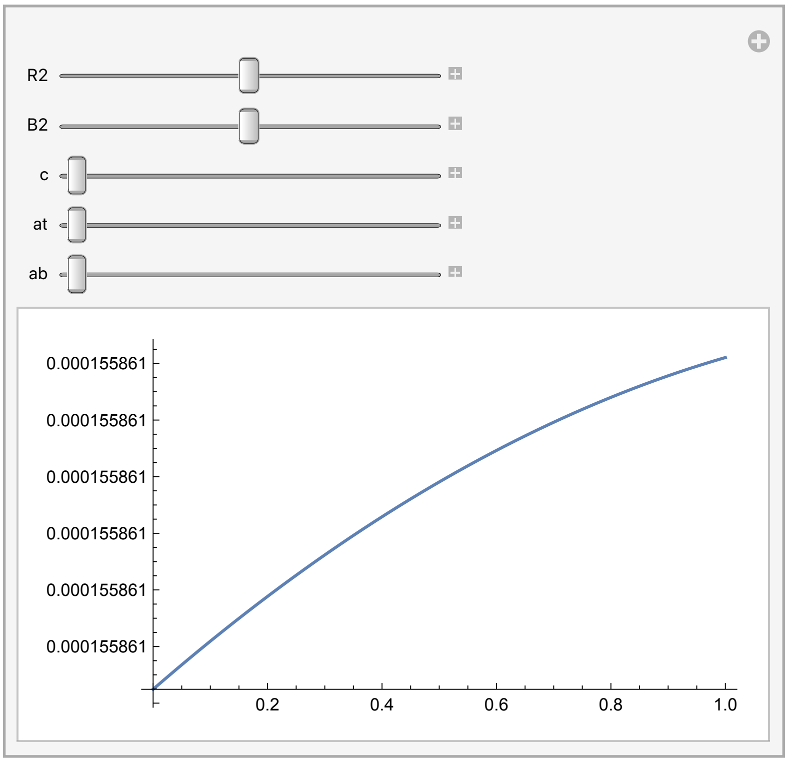

In[ ]:=

```
dbrplot =
  GraphicsRow[Table[Plot[(1/8 ab (-1 + B2) B2 (-1 + R2) R2 (4 + 2 ab R2 - 4 ab B2 (1 + R2) +
            ab^2 (R2^2 - B2 R2 (3 + 7 R2) + B2^2 (4 + 7 R2 + 7 R2^2))) + 3 (1 - 8 B2 + 10 B2^2) sb^2 +
            3 sr^2 - 24 R2 sr^2 + 30 R2^2 sr^2 - 6 at c γ + 12 at B2 c γ + 6 c^2 γ - 12 B2 c^2 γ + 10 c sr
             γ - 20 B2 c sr γ - 20 c R2 sr γ + 40 B2 c R2 sr γ + 12 at c T2 γ - 24 at B2 c T2 γ +
            3 c^2 γ^2 - 24 B2 c^2 γ^2 + 30 B2^2 c^2 γ^2 - 6 (sb - 2 B2 sb + sr - 2 R2 sr + (1 - 2 B2) c γ) +
            2 sb (5 (-1 + 2 B2) (-1 + 2 R2) sr + 6 (1 - 5 B2 + 5 B2^2) c γ) -
            2 ab (2 B2^2 (4 + 7 R2) (sb + c γ) + R2 (3 sb + (3 - 5 R2) sr + 3 c γ) -
               B2 ((5 + 14 R2) sb + (3 - 10 R2^2) sr + c (5 + 14 R2) γ))) /.
        {sr → 0.00001, st → 0.001, sb → 0.001, m → 0.0001, T2 → 0.7986137064685491,
         R2 → 0.5, B2 → 0.5, c → 0.001, at → 0.002}),
      {γ, 0, 1}, PlotRange → Full, PlotStyle → {Thick, Black},
      Frame → True,
      FrameStyle →
       {{{16, Black, FontFamily → "Times"}, {18, Black, FontFamily → "Times"}},
        {{16, Black, FontFamily → "Times"}, {18, Black, FontFamily → "Times"}}},
      FrameLabel → {{Text[Style[ Row[{Subscript["D", "BR"] , " approx"}],
            FontFamily → "Times", FontSize → 22, Black]], None},
         {Text[Style["Effectiveness of Protection, γ", FontFamily → "Times",
            FontSize → 20]], Text[Style[ Row[{Subscript["a", "b"] , "=", ab}],
            FontFamily → "Times", FontSize → 22, Black]]}}],
     {ab, {0.001, 0.003, 0.005}}], ImageSize → 1440]
```

Out[•]=

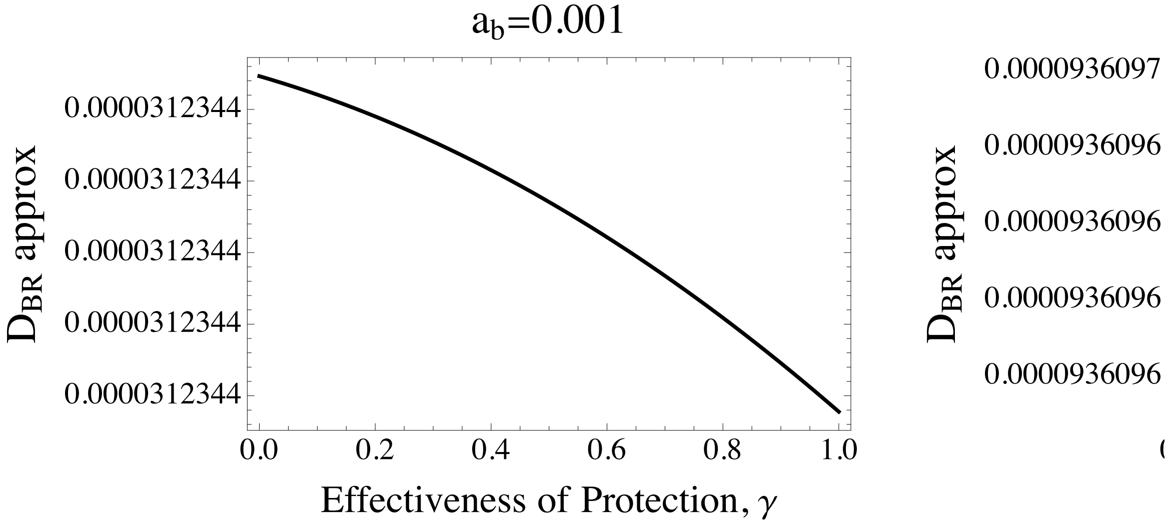

In[•]:=
```
Export["Figure_S2.pdf", dbrplot];
```

In[ ]:=
```
dbrplotc =
 GraphicsRow[Table[Plot[(1/8 ab (-1 + B2) B2 (-1 + R2) R2 (4 + 2 ab R2 - 4 ab B2 (1 + R2) +
         ab^2 (R2^2 - B2 R2 (3 + 7 R2) + B2^2 (4 + 7 R2 + 7 R2^2)) + 3 (1 - 8 B2 + 10 B2^2) sb^2 +
         3 sr^2 - 24 R2 sr^2 + 30 R2^2 sr^2 - 6 at c γ + 12 at B2 c γ + 6 c^2 γ - 12 B2 c^2 γ + 10 c sr
          γ - 20 B2 c sr γ - 20 c R2 sr γ + 40 B2 c R2 sr γ + 12 at c T2 γ - 24 at B2 c T2 γ +
         3 c^2 γ^2 - 24 B2 c^2 γ^2 + 30 B2^2 c^2 γ^2 - 6 (sb - 2 B2 sb + sr - 2 R2 sr + (1 - 2 B2) c γ) +
         2 sb (5 (-1 + 2 B2) (-1 + 2 R2) sr + 6 (1 - 5 B2 + 5 B2^2) c γ) -
         2 ab (2 B2^2 (4 + 7 R2) (sb + c γ) + R2 (3 sb + (3 - 5 R2) sr + 3 c γ) -
            B2 ((5 + 14 R2) sb + (3 - 10 R2^2) sr + c (5 + 14 R2) γ)))) /.
      {sr → 0.00001, st → 0.001, sb → 0.001, m → 0.0001,
       T2 → (1 - m + (2 (1+c+at c) m)/(st+c st+at (-1+c+st)))/(1 + m) /. {m → 0.0001, st → 0.001, at → 0.002},
       R2 → 0.5, B2 → 0.5, ab → 0.005, at → 0.002}),
    {γ, 0, 1}, PlotRange → Full, PlotStyle → {Thick, Black},
    Frame → True,
    FrameStyle →
     {{{16, Black, FontFamily → "Times"}, {18, Black, FontFamily → "Times"}},
      {{16, Black, FontFamily → "Times"}, {18, Black, FontFamily → "Times"}}},
    FrameLabel → {{Text[Style[ Row[{Subscript["D", "BR"] , " approx"}],
          FontFamily → "Times", FontSize → 22, Black]], None},
      {Text[Style["Effectiveness of Protection, γ",
          FontFamily → "Times", FontSize → 20]],
       Text[Style[ Row[{Style["c", FontSlant → Italic] , "=", c}],
          FontFamily → "Times", FontSize → 22, Black]]}}],
   {c, {0.001, 0.003, 0.005}}], ImageSize → 1440]
```

Out[ ]=

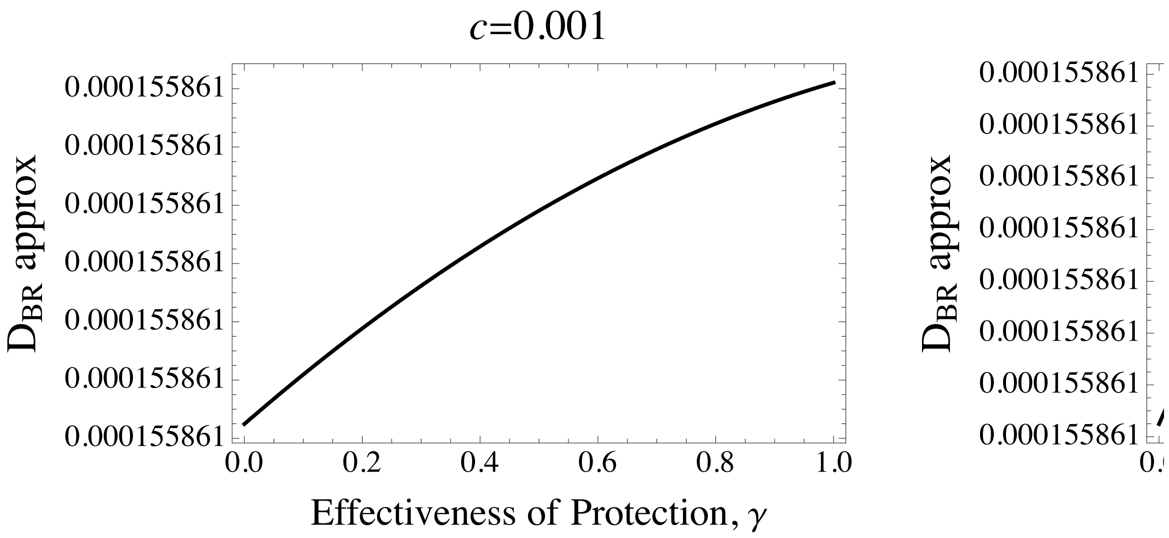

In[ ]:=

```
Export["Figure_S2_b.pdf", dbrplotc];
(*look at expression for DTB*)
Manipulate[
 Plot[-1/2 at (-1 + B2) B2 c (-1 + T2) T2 γ (-2 + 4 m - 3 ab R2 + 6 ab B2 R2 + 3 sb - 6 B2 sb +
      3 st - 6 st T2 + at (-2 + 6 T2) + c (4 + γ - 6 B2 γ)) /.
    {sr → 0.01, st → 0.1, sb → 0.1, m → 0.1, T2 → 0.9}, {γ, 0, 1}, PlotRange → Full],
 {{R2, 0.5}, 0, 1}, {{B2, 0.5}, 0, 1}, {c, 0.2, 0.6},
 {{at, 12}, 4, 16}, {ab, 0, 20}]
```

Out[ ]=

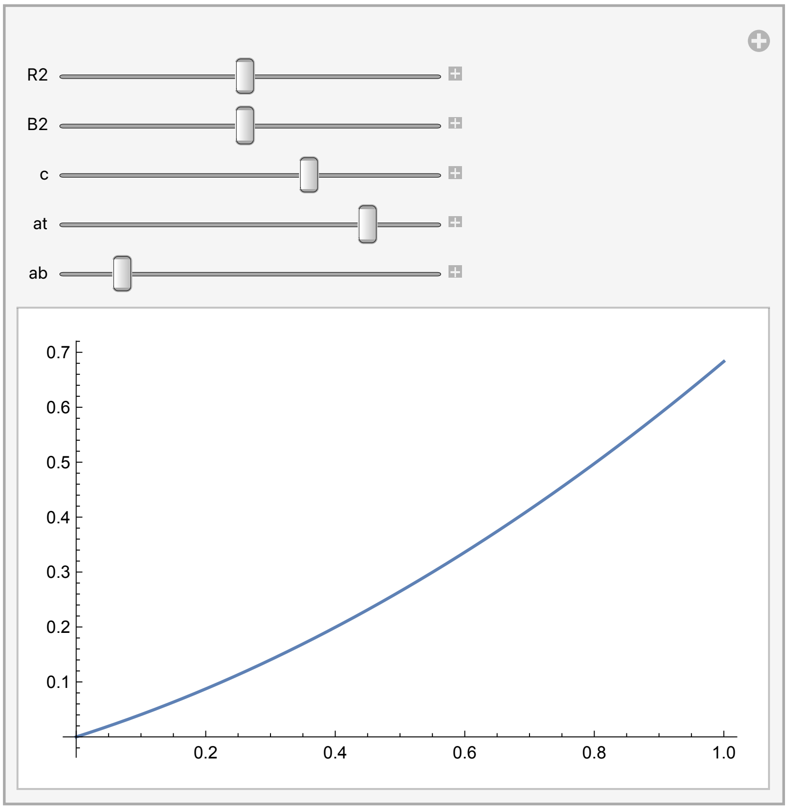

Supplement: qpad074_suppl_Supplementary_Material [file qpad074_suppl_supplementary_material.zip › Remodeling_Supporting_Information_appendicesS1_S3_figs_S1_S5_code_S1_S4.pdf]
